# Preparation of Synthetic and Natural Derivatives of Flavonoids Using Suzuki–Miyaura Cross-Coupling Reaction

**DOI:** 10.3390/molecules27030967

**Published:** 2022-01-31

**Authors:** Martina Hurtová, David Biedermann, Zuzana Osifová, Josef Cvačka, Kateřina Valentová, Vladimír Křen

**Affiliations:** 1 Laboratory of Biotransformation, Institute of Microbiology of the Czech Academy of Sciences, Vídeňská 1083, CZ-14220 Prague, Czech Republic; martina.hurtova@biomed.cas.cz (M.H.); david.biedermann@gmail.com (D.B.); kata.valentova@email.cz (K.V.); 2 Faculty of Food and Biochemical Technology, University of Chemistry and Technology Prague, Technická 5, CZ-16628 Prague, Czech Republic; 3 Institute of Organic Chemistry and Biochemistry of the Czech Academy of Sciences, Flemingovo nám. 542/2, CZ-16000 Prague, Czech Republic; zuzana.osifova@uochb.cas.cz (Z.O.); josef.cvacka@uochb.cas.cz (J.C.)

**Keywords:** flavonoids, Suzuki–Miyaura cross-coupling reaction, prenylation, borylation

## Abstract

Herein, we report the use of the Suzuki–Miyaura cross-coupling reaction for the preparation of a library of synthetic derivatives of flavonoids for biological activity assays. We have investigated the reactivity of halogenated flavonoids with aryl boronates and with boronyl flavonoids. This reaction was used to prepare new synthetic derivatives of flavonoids substituted at C-8 with aryl, heteroaryl, alkyl, and boronate substituents. The formation of flavonoid boronate enabled a cross-coupling reaction with halogenated flavones yielding biflavonoids connected at C-8. This method was used for the preparation of natural compounds including C-8 prenylated compounds, such as sinoflavonoid NB. Flavonoid boronates were used for the preparation of rare C-8 hydroxyflavonoids (natural flavonoids gossypetin and hypolaetin). A series of previously unknown derivatives of quercetin and luteolin were prepared and fully characterized.

## 1. Introduction

**Figure 1:**
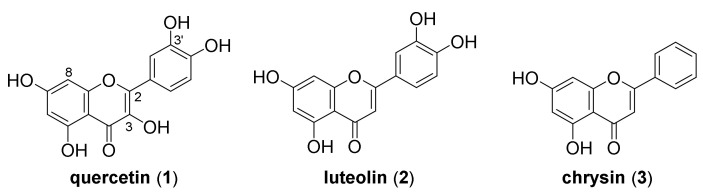
Structure of selected flavonoids.

Flavonoids are a large class of natural polyphenolic secondary metabolites that are a common component of the human diet. Flavonoids generally have low toxicity and possess numerous beneficial biological activities such as antioxidant, anti-inflammatory, hepatoprotective, and anticarcinogenic properties [1]. Quercetin (**1**; 3,3′,4′,5,7-pentahydroxyflavone) is a bioactive flavonol found as a plant pigment mainly in onions, apples, and citrus fruits. It is used as a dietary supplement because it reduces oxidative stress, inhibits low-density lipoprotein oxidation and platelet aggregation, and acts as a vasodilator in blood vessels [2]. Luteolin (**2**; 3′,4′,5,7-tetrahydroxyflavone) is a common flavone found in fruits, vegetables, and medicinal plants. Luteolin (**2**) is known for its anti-allergic and anti-cancer activity [3]. Another flavone, chrysin (**3**; 5,7-dihydroxyflavone), occurs naturally in many plants, honey, and propolis and exhibits antioxidant, anti-inflammatory, anticancer, and antiviral properties (Figure 1) [4].

In this study, we focused on the preparation of new synthetic derivatives of the flavonoids quercetin (**1**), luteolin (**2**), and chrysin (**3**) substituted on the A-ring with alkyl or aryl substituents using palladium-catalyzed cross-coupling reactions.

Cross-coupling reactions are metal-promoted hetero couplings used to make new C–C or C–N bonds. These reactions typically use halide and a coupling partner (organoboronate, organosilicate, or amine) in the presence of a transition metal catalyst (Pd, Ni, or Zn) and a base [5]. Widely used palladium-catalyzed cross-coupling reactions include Heck coupling, Negishi, Stille, and Suzuki–Miyaura cross-coupling [6]. Buchwald–Haartwig amination is a Pd-catalyzed coupling reaction of aryl (pseudo)halides with amines used to form C(sp^2^)-N bonds [7,8].

The Suzuki cross-coupling reaction has been described previously for the preparation of functionalized flavones [9]. Brominated derivatives of flavones were arylated with phenylboronic acid and 4-fluorophenylboronic acid. Secondary aryl amines were prepared using the Buchwald–Hartwig amination reaction [9]. Kimura et al. reported the preparation of arylated derivatives using 8-iodo-3,5,7,3′,4′-penta-*O*-methyl quercetin, and phenyl- or naphthylboronic acid. The prepared derivatives were reduced with LiAlH_4_ affording 8-aryl-3,5,7,3′,4′-penta-*O-*methylcyanidins [10]. Kohari et al. reported the preparation of symmetrical and unsymmetrical biflavones linked via the A-ring using bromoflavone and pinacolatoborylflavone [11]. This reaction was also used for the total synthesis of robustaflavone, a biflavonoid consisting of two 5,7,4′-trihydroxyflavone units linked through a biaryl linkage between the C-6 and C-3′ [12]. The Suzuki–Miyaura cross-coupling reaction was used to prepare 8-(6″-umbelliferyl)-apigenin and its analogs [13]. 8-Iodo-5,7-dimethoxy chrysin was used for the preparation of a boronate, which was later oxidized with NaBO_3_. 4 H_2_O to generate a new hydroxy group at C-8 [14]. This method can be used as an alternative for the preparation of gossypetin (3,5,7,8,3′,4′-hexahydroxyflavone) and hypolaetin (8-hydroxyluteolin). Gossypetin is a C-8-hydroxy derivative of quercetin extracted from the flowers of *Hibiscus sabdariffa* (roselle) and has a potent antibacterial activity [15]. Gossypetin was previously prepared using flavonoid 8-hydroxylase, an enzyme isolated from *Chrysanthemum segetum* [16]. Gossypetin was also prepared by acid hydrolysis of gossypetin glycosides isolated from *Feijoa sellowiana* [17].

Prenylated flavonoids are secondary plant metabolites that are considered to act as phytoalexins and play an important role in defense against pathogenic organisms [18]. Prenylation at C-8 increases the lipophilicity of flavonoids and their affinity for biological membranes [18]. Prenylated derivatives of flavonoids have many beneficial biological effects, such as the ability to inhibit P-glycoprotein (Pgp), which is related to the modulation of multidrug resistance [19]; they were also reported to have strong antioxidant activity [20,21]. Prenylated derivatives of flavonoids are generally prepared by Claisen rearrangement of *O*-prenylated compounds or by direct C-alkylation in alkaline solution [22]. These synthetic methods have many drawbacks, mainly low yields and complicated separation of the products. Suzuki–Miyaura cross-coupling is an interesting alternative for the preparation of prenylated and alkylated derivatives of flavonoids. This reaction has not been previously described for the prenylation of flavonoids. Prenylated benzene was prepared by a Suzuki cross-coupling reaction using benzylboronic acid and prenyl bromide [23]. The prenyl group can also cyclize to form 2,2-dimethyldihydropyranone group, resulting in sinoflavonoids [24].

Biflavonoids are a small subclass of flavonoids, and their occurrence in nature is rare. Biflavonoids are flavonoid dimers consisting of two identical or non-identical flavonoid/flavone units linked by a C–C bond [25]. Biflavonoids have been reported to have anti-amyloidogenic effects by inhibiting aggregation of Aβ plaques and promoting the disaggregation of Aβ fibrils; this effect makes biflavonoids lead compounds in the treatment of Alzheimer’s disease [26]. The preparation of biflavonoids using metal-catalyzed cross-coupling reactions has been previously reported in the literature. Palladium-catalyzed coupling of aryl triflates with organostannanes yielded biflavonoids linked via C-7 [27]. Suzuki cross-coupling reaction of boronate and iodo-flavonoid yielded biflavonoid connected via C-8 (ring A) and C-3’(ring B) [28]. Kohari et al. reported the preparation of biflavones by Suzuki cross-coupling of boronylflavone and bromoflavone (flavones without HO- groups) [11]. Ulmann condensation of halogenated flavonoids was reported for the preparation of ginkgetin, a natural biflavone first isolated from *Ginkgo biloba* [29]. To our knowledge, the preparation of biflavonoids linked via C-8 has not yet been described in the literature.

## 2. Results and Discussion

8-Bromo-3,3′,4′,5,7-penta-*O*-isopropylquercetin (**4**) was prepared using α,β-dibromohydrocinnamic acid [30]. The Suzuki cross-coupling reaction was carried out using 8-bromo derivative (**4**) and 3-thienylboronic acid under various conditions (Table 1). The outcome of this reaction was the desired reaction product **6** (isolated yield ca 8–10%), a product of debromination (3,3′,4′,5,7-penta-*O*-isopropylquercetin, 80%) and unreacted starting material (10%). Partial dehalogenation of the starting material is one of the drawbacks of the Suzuki cross-coupling reaction carried out in aqueous conditions. The outcome of this reaction indicates that the reaction stops after the oxidative addition step of the cross-coupling reaction. Varying the amount of base, boronic acid, or palladium catalyst did not affect the result of this reaction. 

8-Iodo-3,3′,4′,5,7-penta-*O*-isopropylquercetin (**5**), 8-iodo-3′,4′,5,7-tetra-*O*-isopropylluteolin (**12**), and 8-iodo-5,7-di-*O*-isopropyl chrysin were prepared with *N*-iodosuccinimide in excellent yields (90–100%) [13]. 8-Iodo-3,3′,4′,5,7-penta-*O*-isopropylquercetin (**5**) was subsequently used for the Suzuki cross-coupling reaction. The outcome of this reaction was product **6** of the cross-coupling reaction in ca 24% yield (Entry 5, Table 1). Under the same reaction conditions, 50% of the desired product was obtained upon microwave (MW) irradiation (120 °C, 2 h, Entry 6, Table 1). The products of the cross-coupling reaction were isolated in excellent yields after optimization of the reaction conditions (Pd(PPh_3_)_4_, NaOH, DMF, 10% H_2_O, 120 °C, 2 h, MW). The optimization of the reaction conditions with the isolated yields is summarized in Table 1. The optimized reaction conditions were used for the reactions with various boronic acids. In general, the reactions with aromatic boronic acids substituted with electron-donating groups or without substitution proceeded in good yields. In the case of aromatic boronic acids substituted with electron-withdrawing groups, the isolated yields were lower (30%), which was probably due to the low reactivity of the boronic acids, as the decomposition of the starting material was faster than the cross-coupling reaction. Exceptions were the reactions with *tert*-butylphenylboronic acid with a lower isolated yield (25%) and 4-trifluoromethylphenylboronic acid with a good isolated yield (71%). Low yields were also obtained after the cross-coupling reaction with 4-pyridinylboronic acid and pyrimidine-5-boronic acid due to their low reactivity towards cross-coupling reactions. The reaction with 4-mercaptophenylboronic acid and 2-fluoropyridine-4-boronic acid was unsuccessful.

Borylation of 8-bromo-3,3′,4′,5,7-penta-*O*-isopropylquercetin (**4**) and 8-iodo-3,3′,4′,5,7-penta-*O*-isopropylquercetin (**5**) using Miyaura borylation conditions with bis(pinacolato)diboron afforded product of dehalogenation and unreacted starting material. This problem was previously attributed to a strong steric effect of bis(pinacolato)diboron [14]. Borylated quercetin (**7**) was prepared in quantitative yield using pinacolborane (HBpin; 4,4,5,5-tetramethyl-1,3,2-dioxaborolane) and 8-iodo-3,3′,4′,5,7-penta-*O*-isopropylquercetin (**5**) (TEA, THF, 2 h, 70 °C, MW).

**Scheme 1:**
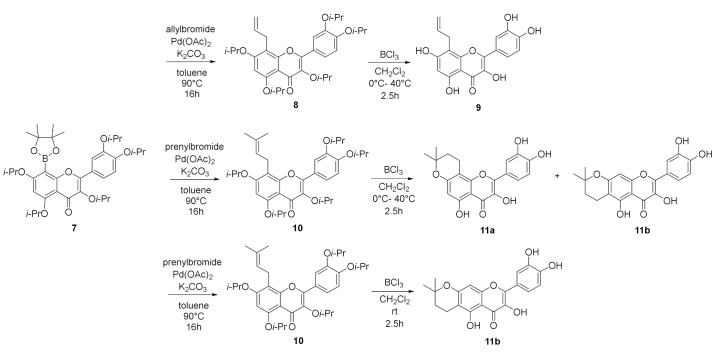
Preparation of quercetin derivatives.

The cross-coupling reaction of 8-iodo-3,3′,4′,5,7-penta-*O*-isopropylquercetin (**5**) with allylboronic acid pinacol ester failed using optimized reaction conditions (Pd(PPh_3_)_4_, NaOH, DMF/H_2_O, 120 °C, 2 h, MW; PEPPSI-*i*Pr, 5 M KOH, THF, reflux, 16 h). The allyl derivative of quercetin (**8**) was prepared using allyl bromide and 8-boronyl-3,3′,4′,5,7-penta-*O*-isopropylquercetin (**5**) (Scheme 1). The reaction of prenyl bromide with 8-boronyl-3,3′,4′,5,7-penta-*O*-isopropylquercetin (**7**) yielded a C-8 prenylated product (**10**) (confirmed by NMR analysis, Figures S29 and S30). After deprotection of the isopropyl ether groups with 1 M boron trichloride in dichloromethane, the C-8 prenyl group cyclized with HO-7 forming 7,8-(2,2-dimethyldihydropyrano)quercetin (sinoflavonoid NB) (**11a**) and 7,6-(2,2-dimethyldihydropyrano)quercetin (**11b**) (Scheme 1). Compound **11a** is a natural compound while compound **11b** is a structural analog of naturally occurring sinoflavonoid NF (3-OMe group). Sinoflavonoids are a group of prenylated flavonoids previously isolated from the fruits of *Sinopodophyllum hexandrum* [24,31]. It was previously reported that sinoflavonoids have anticancer activity towards breast cancer cell lines [31]. Synthetic preparation of sinoflavonoids has not yet been reported in the literature.

8-Boronyl-3,3′,4′,5,7-penta-*O*-benzylquercetin was prepared to avoid cyclization of the prenyl group by using hydrogen for deprotection of the benzyl group. Application of the optimized method for cross-coupling of the boronate with allyl- and prenyl bromide failed. This reaction was probably unsuccessful due to the steric hindrance of the benzyl group and the lower reactivity of alkyl bromides in cross-coupling reactions. Generally, all cross-coupling reactions tested with benzylated flavonoids were slower, and the reaction time had to be prolonged to achieve conversion to product, and microwave irradiation had no effect at all in these reactions. After adjusting the reaction conditions (Pd(PPh_3_)_4_, CsF, THF, 70 °C, 3 days), the reaction of 8-iodo-3,3′,4′,5,7-penta-*O*-benzylquercetin (**5**) with allylboronic acid pinacol ester or prenylboronic acid pinacol ester succeeded in 60% and 50% yield, respectively. Both products were isolated but the deprotection using H_2_ on Pd/C and also Pd(OH)_2_ was unsuccessful. We tried deprotection of 3,3′,4′,5,7-penta-*O*-benzylquercetin, and this reaction afforded deprotected quercetin. It appears that the C-8 substitution is in some cases preventing the deprotection. The problem with deprotection was also in the case of **26** where the HO-7 isopropyl group was not deprotected after repeated treatment with 1 M BCl_3_.

**Scheme 2:**
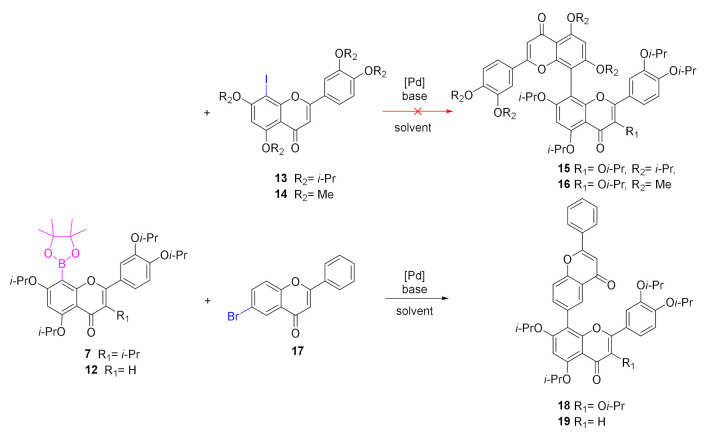
Preparation of biflavonoids connected via C-8.

The preparation of biflavonoids by cross-coupling reaction between 8-iodo-3′,4′,5,7-tetra-*O*-isopropylluteolin (**13**) and 8-boronyl-3′,4′,3,5,7-penta-*O*-isopropylquercetin (**7**) failed under various reaction conditions. The products of these reactions were products of dehalogenation and deborylation of the starting materials. This reaction was also unsuccessful under other reaction conditions. The reactivity of boronate was studied to determine the effect of *o*-substitution. The reaction of 8-boronyl-3,3′,4′,5,7-penta-*O*-isopropylquercetin (**7**), and 6-bromoflavone (**17**) gave the product in 70% yield. This reaction proved that the low reactivity of iodo- and boronyl-flavonoids in the formation of biflavonoids is related to steric hindrance of the protecting group in the *o*-position and that the reaction proceeds when no substituent is present on the A-ring of the coupling partner. Pan et al. reported the synthesis of 8-(6″-umbelliferyl)-quercetin cross-coupling reaction of 8-iodo-3′,4′,3,5,7-penta-*O*-isopropylquercetin and 7-methoxy-6-boronylcoumarin in 73% yield, suggesting that the cross-coupling should work for substrates protected in the *o*-position [13]. To determine whether the reaction would also work with a sterically less demanding protecting group, cross-coupling of 8-boronyl-3,3′,4′,5,7-penta-*O*-isopropylquercetin (**7**) and 8-iodo-3′,4′,5,7-tetra-*O*-methylluteolin (**14**) was performed but was also unsuccessful. This reaction confirmed that the low reactivity of flavonoids in our experiments to form C-8-linked biflavonoids was related to the steric hindrance of protecting groups on the A-rings of coupling partners (Scheme 2).

**Figure 2:**
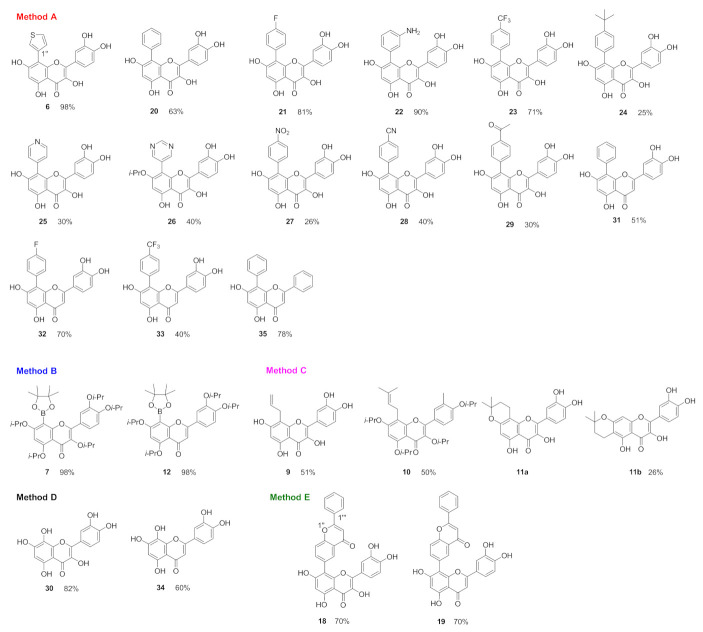
Prepared derivatives and isolated yields. Method A: 8-iodoflavonoid, boronic acid, Pd(PPh_3_)_4_, NaOH, DMF/H_2_O, MW, 120 °C, 2 h; Method B: 8-iodoflavonoid, Pd(OAc)_2_, P(*o*-Tol)_3_, NEt_3_, HBpin, THF, MW, 70 °C, 2 h; Method C: 8-boronylflavonoid, K_2_CO_3_, Pd(OAc)_2_, alkyl bromide, toluene, 90 °C, 48 h; Method D: 8-boronylflavonoid, sodium perborate tetrahydrate, THF/H_2_O, rt, 3 h; Method E: 6-bromoflavone, 8-boronylflavonoid, Pd(PPh_3_)_4_, Cs_2_CO_3_, dioxane, 100 °C, 3 days. All products were deprotected using BCl_3_ in dichloromethane.

The main goal of our work was to create a library of compounds for biological activity testing. According to our preliminary experiments, the ether derivatives of flavonoids are highly cytotoxic to the tested cell lines. For further biological assays, all prepared products were deprotected with 1 M BCl_3_ in dichloromethane and fully characterized by ^1^H, ^13^C, COSY, HMBC, and HSQC NMR. All derivatives prepared and isolated yields are summarized in Figure 2.

## 3. Materials and Methods

### 3.1. General Information

Procedures involving oxygen- or moisture-sensitive materials were performed with anhydrous solvents (*vide infra*) under a nitrogen atmosphere in flame-dried flasks, using standard Schlenk techniques. Analytical TLC was performed on Al plates (Silica Gel 60 F_254_; Merck, Prague, Czech Republic). Purification was performed using the preparative HPLC system (Shimadzu, Kyoto, Japan). Preparative HPLC separations were performed using an ASAHIPAK GS-310 20F column (5 mL/min. MeOH isocratic; Shodex, Munich, Germany). All analytical HPLC separations were performed with the Shimadzu Prominence System (Shimadzu, Kyoto, Japan). An ARION Polar C18 HPLC column (3 µm, 100 × 3 mm i.d.; Chromservis, Prague, Czech Republic) coupled an ARION guard column (5 × 4 mm, Chromservis, Prague, Czech Republic) was used. Mobile Phase A CH_3_CN/H_2_O/HCOOH (5:95:0.1), and Phase B CH_3_CN/H_2_O/HCOOH (80:20:0.1), were employed in the analyses. Mobile Phase A CH_3_CN/H_2_O/HCOOH (5:95:0.1), and Phase B CH_3_CN/HCOOH (100:0.1), were used for the analyses of protected flavonoids. The NMR analyses were carried out on spectrometers Bruker Avance III^TM^ HD 600 MHz equipped with a cryoprobe (^1^H 600 and ^13^C 151 MHz), Bruker Avance III^TM^ HD 500 MHz equipped with a cryoprobe (^1^H 500, ^13^C 126, and ^19^F 470 MHz), Bruker Avance II^TM^ 500 MHz (^1^H 500 and ^13^C 126 MHz), and Bruker Avance III^TM^ 400 MHz (^1^H 400, ^13^C 100, and ^19^F 376 MHz) instrument (Bruker, Karlsruhe, Germany) in DMSO-*d*_6_, at 25 °C. The signal in DMSO-*d*_6_ was used as a reference (*δ*_H_ 2.499, *δ*_C_ 39.46).The ^1^H-NMR spectra and ^13^C-NMR spectra of the compounds **6**, **20**, **21**, **22**, **23**, **24**, **25**, **26**, **27**, **28**, **29**, **7**, **9**, **10**, **11**, **30**, **31**, **32**, **33**, **12**, **34**, **35**, **18**, and **19** can be found in the Supplementary Materials (Figures S1–S52). Mass spectra were measured using LTQ Orbitrap XL hybrid mass spectrometer (Thermo Fisher Scientific, Waltham, MA, USA) equipped with an electrospray ion source and operated at the resolution of 100,000. The samples were loop injected into methanol/water (4:1), a flow rate of 100 µL/min. Reactions under microwave irradiation were carried out in microwave reactor Biotage Initiator + (Biotage, Uppsala, Sweden) using Biotage microwave reaction vials (borosilicate glass, 0.2–20 mL) equipped with aluminium cap and Teflon septa. Commercially available reagents and dried solvents were purchased from Sigma Aldrich (Darmstadt, Germany), Alfa Aesar (Ward Hill, MA, USA), Acros Organics (Morris Plains, NJ, USA), and TCI chemicals (Gurugram, India) and used without further purification unless otherwise stated. Infrared spectra were recorded on an FT-IR spectrometer Nicolet 6700 (Thermo Scientific, Waltham, MA, USA) using a standard MIR source, KBr beamsplitter, and DTGS detector, which was purged with nitrogen. For measurements, only transmission mode (64 scans, 2 cm^–1^ spectral resolution, Happ–Genzel apodization function) was used. The sample was dissolved in methanol, chloroform, or KBr pellet was prepared (diameter 4 mm). The FT-IR spectra of the compounds **6**, **20**, **21**, **22**, **23**, **24**, **25**, **27**, **28**, **29**, **7**, **9**, **30**, **31**, **32**, **33**, **12**, **34**, **18**, and **19** can be found in the Supplementary Materials (Figures S53–S72).

### 3.2. Experimental Procedures

#### 3.2.1. General Procedure A for Suzuki Cross-Coupling Reaction

8-Iodo flavonoid (1 eq) and the corresponding boronic acid (2 eq) were dissolved in a DMF/H_2_O mixture (9:1) in a microwave vial. The reaction mixture was degassed with nitrogen for 15 min. Pd(PPh_3_)_4_ (3 mol%) and NaOH (4 eq) were added. The reaction mixture was irradiated in a microwave reactor at 120 °C for 2 h. After the reaction mixture cooled to ambient temperature, it was filtered through a microfilter (PTFE, 0.45 µm). The reaction mixture was poured into water and extracted with dichloromethane (3 × 10 mL). The combined organic layers were washed with water (3 × 10 mL), brine (3 × 10 mL), dried over Na_2_SO_4,_ and evaporated to dryness in vacuo. The residue was dissolved in dry dichloromethane (5 mL) at −10 °C. A solution of boron trichloride (10 eq, 1 M solution in dichloromethane) was added to the reaction mixture, which was then stirred at −10 °C for 30 min. The reaction mixture was then heated to 40 °C and stirred for 2 h, then cooled to 0 °C, and an excess of methanol was added. The reaction mixture was evaporated in vacuo, and the residue was purified by preparative HPLC chromatography (ASAHIPAK, 5 mL/min, MeOH isocratic) yielding the corresponding product.

*8-Thienyl quercetin* (**6**): General method A was followed using 8-iodo-3,3′,4′,5,7-penta-*O*-isopropylquercetin (**5**), and 3-thienylboronic acid, yielding **6** as a green solid (119 mg, 98%). HRMS (ESI, *m*/*z*): calcd for C_19_H_11_O_7_S [M-H]^−^: 383.02310, found: 383.02236. ^1^H NMR (500 MHz, DMSO-*d*_6_) δ 12.71 (s, 1H, HO-5), 10.84 (s, 1H, HO-7), 9.63 (s, 1H, HO-4′), 9.41 (s, 1H, HO-3), 9.15 (s, 1H, HO-3′), 7.64 (dd, *J* = 2.9, 1.2 Hz, 1H, H-2″), 7.61 (m, 2H, H-2′, 4″), 7.31 (dd, *J* = 4.9, 1.3 Hz, 1H, H-5″), 7.26 (dd, *J* = 8.5, 2.2 Hz, 1H, H-6′), 6.76 (d, *J* = 8.5 Hz, 1H, H-5′), 6.39 (s, 1H, H-6) ppm. ^13^C NMR (126 MHz, DMSO-*d*_6_) δ 176.1 (C-4), 161.1 (C-7), 159.3 (C-5), 153.1 (C-9), 147.7 (C-4′), 147.2 (C-2), 144.9 (C-3′), 135.6 (C-3), 130.9 (C-1″), 130.2 (C-5″), 125.2 (C-4″), 124.3 (C-2″), 122.1 (C-1′), 119.7 (C-6′), 115.7 (C-2′), 115.3 (C-5′), 103.2 (C-10), 102.9 (C-8), 98.0 (C-6) ppm. IR (MeOH film): 1648 (s), 1602 (s), 1559 (s), 1507 (s), 1363 (m), 1203 (s), 1170 (s), 1109 (m), 1009 (m) cm^−1^. *8-Phenyl quercetin* (**20**): General method A was followed using 8-iodo-3,3′,4′,5,7-penta-*O*-isopropylquercetin (**5**) and phenylboronic acid, affording **20** as a yellow solid (70 mg, 63%). HRMS (ESI, *m*/*z*): calcd for C_21_H_13_O_7_ [M-H]^−^: 377.06668, found: 377.06616. ^1^H NMR (400 MHz, DMSO-*d*_6_) δ 12.68 (s, 1H, HO-5), 10.72 (s, 1H, HO-7), 9.60 (s, 1H, HO-4′), 9.40 (s, 1H, HO-3), 9.08 (s, 1H, HO-3′), 7.55 (d, *J* = 2.2 Hz, 1H, H-2′), 7.50–7.35 (m, 5H, H-2″, 3″,4″, 5″, 6″), 7.09 (dd, *J* = 8.5, 2.2 Hz, 1H, H-6′), 6.69 (d, *J* = 8.5 Hz, 1H, H-5′), 6.41 (s, 1H, H-6) ppm. ^13^C NMR (100 MHz, DMSO-*d*_6_) δ 176.1 (C-4), 160.8 (C-7), 159.4 (C-5), 153.0 (C-9), 147.6 (C-4′), 147.0 (C-2), 144.9 (C-3′), 135.6 (C-3), 132.0 (C-1″), 131.1 (C-3″, 5″), 127.8 (C-2″, 6″), 127.1 (C-4″), 122.1 (C-1′), 119.5 (C-6′), 115.7 (C-2′), 115.2 (C-5′), 107.6 (C-8), 103.1 (C-10), 98.0 (C-6) ppm. IR (MeOH film): 1650 (m), 1624 (sh), 1598 (m), 1560 (m), 1541 (m), 1490 (m), 1193 (s), 1022 (m), 999 (s), 701 (m), 686 (m) cm^−1^.

*8-(4-Fluorophenyl) quercetin* (**21**): General method A was followed using 8-iodo-3,3′,4′,5,7-penta-*O*-isopropylquercetin (**5**) and 4-fluorophenylboronic acid, affording **21** as a green solid (99.5 mg, 81%). HRMS (ESI, *m*/*z*): calcd for C_21_H_12_O_7_F [M-H]^−^: 395.05725, found: 395.05661. ^1^H NMR (400 MHz, DMSO-*d*_6_) δ 12.69 (s, 1H, HO-5), 10.77 (s, 1H, HO-7), 9.60 (s, 1H, HO-4′), 9.40 (s, 1H, HO-3), 9.11 (s, 1H, HO-3′), 7.49 (m, 3H, H-2″, 6″, 2′), 7.30 (m, 2H, H-3″, 5″), 7.13 (dd, *J* = 8.5, 2.2 Hz, 1H, H-6′), 6.73 (d, *J* = 8.5 Hz, 1H, H-5′), 6.40 (s, 1H, H-6) ppm. ^13^C NMR (100 MHz, DMSO-*d*_6_) δ 176.1 (C-4), 161.28 (d, *J* = 243.5 Hz, C-4″), 160.8 (C-7), 159.5 (C-5), 153.0 (C-9), 147.6 (C-4′), 147.1 (C-2), 144.9 (C-3′), 135.6 (C-3), 133.1 (d, *J* = 8.1 Hz, C-2″, 6″), 128.2 (d, *J* = 3.0 Hz, C-1″), 122.1 (C-1′), 119.6 (C-6′), 115.6 (C-2′), 115.3 (C-5′), 114.9 (d, *J* = 21.2 Hz*,* C-3″, 5″), 106.5 (C-8), 103.1 (C-10), 98.0 (C-6) ppm. ^19^F NMR (376 MHz, DMSO-*d*_6_) δ-115.15 ppm. IR (MeOH film): 1652 (m), 1620 (sh), 1598 (m), 1551 (m), 1506 (s), 1192 (s), 1160 (m), 1022 (m), 1000 (s), 763 (m) cm^−1^.

*8-(3-Aminophenyl) quercetin* (**22**): General method A was followed using 8-iodo-3,3′,4′,5,7-penta-*O*-isopropylquercetin (**5**) and 3-aminophenylboronic acid, yielding **22** as a green solid (111 mg, 90%). HRMS (ESI, *m*/*z*): calcd for C_21_H_14_O_7_N [M-H]^−^: 392.07758, found: 392.07751. ^1^H NMR (500 MHz, DMSO-*d*_6_) δ 12.71 (bs, 1H, HO-5), 11.01 (s, 1H, HO-7), 9.95 (bs, 2H, NH_2_), 9.66 (bs, 1H, HO-4′), 9.44 (bs, 1H, HO-3), 9.15 (bs, 1H, HO-3′), 7.61–7.53 (m, 2H, H-5″, 2′), 7.44 (d, *J* = 7.6 Hz, 1H, H-6″), 7.37 (s, 1H, H-2″), 7.32 (d, *J* = 7.9 Hz, 1H, H-4″), 7.07 (dd, *J* = 8.4, 2.2 Hz, 1H, H-6′), 6.75 (d, *J* = 8.5 Hz, 1H, H-5′), 6.47 (s, 1H, H-6) ppm. ^13^C NMR (126 MHz, DMSO-*d*_6_) δ 176.1 (C-4), 160.8 (C-7), 159.8 (C-5), 152.9 (C-9), 147.7 (C-4′), 147.1 (C-2), 144.9 (C-3′), 135.7 (C-3), 133.6 (C-1″), 129.2 (C-5″, 6″), 124.7 (C-2″), 121.1 (C-4″), 122.0 (C-1′), 119.5 (C-6′), 115.7 (C-2′), 115.5 (C-5′), 106.3 (C-8), 103.1 (C-10), 98.0 (C-6) ppm. IR (KBr pellet): 1653 (s), 1624 (s), 1601 (s), 1559 (m), 1515 (s), 1491 (m), 1203 (s), 1003 (m), 999 (s), 759 (m), 698 (m) cm^−1^.

*8-(4-Trifluoromethylphenyl) quercetin* (**23**): General method A was followed using 8-iodo-3,3′,4′,5,7-penta-*O*-isopropylquercetin (**5**) and 4-(trifluoromethyl)phenylboronic acid, affording **23** as a yellow solid (144.3 mg, 71%). HRMS (ESI, *m*/*z*): calcd for C_22_H_14_O_7_F_3_ [M + H]^+^: 447.06861, found: 447.06877. ^1^H NMR (500 MHz, DMSO-*d*_6_) δ 12.74 (s, 1H, HO-5), 10.95 (s, 1H, HO-7), 9.59 (s, 1H, HO-4′), 9.45 (s, 1H, HO-3), 9.10 (s, 1H, HO-3′), 7.86–7.79 (m, 2H, H-3″, 5″), 7.74–7.69 (m, 2H, H-2″, 6″), 7.49 (d, *J* = 2.2 Hz, 1H, H-2′), 7.07 (dd, *J* = 8.5, 2.2 Hz, 1H, H-6′), 6.70 (d, *J* = 8.5 Hz, 1H, H-5′), 6.42 (s, 1H, H-6) ppm. ^13^C NMR (126 MHz, DMSO-*d*_6_) δ 176.1 (C-4), 160.8 (C-7), 160.1 (C-5), 153.0 (C-9), 147.7 (C-4′), 147.2 (C-2), 144.9 (C-3′), 136.6 (C-1″), 135.7 (C-3), 132.0 (C-2″, 6″), 127.5 (q, *J* = 31.6 Hz, C-4″), 124.7 (q, *J* = 3.8 Hz, C-3″, 5″), 124.5 (q, J = 272.5 Hz, CF_3_), 122.0 (C-1′), 119.5 (C-6′), 115.6 (C-2′), 115.2 (C-5′), 106.1 (C-8), 103.1 (C-10), 98.0 (C-6) ppm. ^19^F NMR (470 MHz, DMSO-*d*_6_) δ-60.84 ppm. IR (MeOH film): 1651 (m), 1624 (sh), 1605 (m), 1563 (m), 1511 (m), 1326 (s), 1189 (m), 1009 (m), 1002 (m) cm^−1^.

*8-(4-*tert*-Butylphenyl) quercetin* (**24**): General method A was followed using 8-iodo-3,3′,4′,5,7-penta-*O*-isopropylquercetin (**5**) and 4-(*tert-*butylphenyl)phenylboronic acid, affording **24** as a yellow solid (33.7 mg, 25%). HRMS (ESI, *m*/*z*): calcd for C_25_H_21_O_7_ [M-H]^−^: 433.12928, found: 433.12852. ^1^H NMR (400 MHz, DMSO-*d*_6_) δ 12.61 (s, 1H, HO-5), 10.69 (s, 1H, HO-7), 9.56 (s, 1H, HO-4′), 9.40 (s, 1H, HO-3), 9.05 (s, 1H, HO-3′), 7.59 (d, *J* = 2.2 Hz, 1H, H-2′), 7.50 (d, *J* = 8.4 Hz, 1H, H-3″, 5″), 7.37 (d, *J* = 8.3 Hz, 1H, H-2″, 6″), 7.05 (dd, *J* = 8.5, 2.2 Hz, 1H, H-6′), 6.66 (d, *J* = 8.5 Hz, 1H, H-5′), 6.40 (s, 1H, H-6), 1.36 (s, 9H, CH_3_ -*t*Bu) ppm. ^13^C NMR (100 MHz, DMSO-*d*_6_) δ 176.1 (C-4), 160.8 (C-7), 159.3 (C-5), 153.1 (C-9), 149.4 (C-1″), 147.6 (C-4′), 146.9 (C-2), 144.9 (C-3′), 135.6 (C-3), 130.8 (C-2″, 6″), 129.0 (C-4″), 124.6 (C-3″, 5″), 122.1 (C-1′), 119.5 (C-6′), 115.9 (C-2′), 115.1 (C-5′), 107.5 (C-8), 103.1 (C-10), 98.0 (C-6), 34.4 (C*_q_*-*t*Bu), 31.2 (CH_3_ -*t*Bu) ppm. IR (MeOH film): 1650 (m), 1624 (sh), 1602 (m), 1555 (m), 1512 (s), 1391 (m),1367 (m), 1191 (s), 1022 (m), 999 (s) cm^−1^.

*8-(4-Pyridinyl) quercetin* (**25**): General method A was followed using 8-iodo-3,3′,4′,5,7-penta-*O*-isopropylquercetin (**5**) and 4-pyridinylboronic acid, affording **25** as a yellow solid (35 mg, 30%). HRMS (ESI, *m*/*z*): calcd for C_20_H_14_O_7_N [M + H]^+^: 380.07648, found: 380.07682. ^1^H NMR (500 MHz, DMSO-*d*_6_) δ 12.74 (s, 1H, HO-5), 11.43 (s, 1H, HO-7), 9.83 (s, 1H, HO-4′), 9.43 (s, 1H, HO-3), 9.18 (s, 1H, HO-3′), 8.69–8.63 (m, 2H, H-3″, 5″), 7.54–7.46 (m, 3H, H-2″, 6″, 2′), 7.15 (dd, *J* = 8.5, 2.2 Hz, 1H, H-6′), 6.79 (d, *J* = 8.5 Hz, 1H, H-5′), 6.63 (s, 1H, H-6) ppm. ^13^C NMR (126 MHz, DMSO-*d*_6_) δ 176.0 (C-4), 161.1 (C-7), 160.3 (C-5), 152.8 (C-9), 149.2 (C-3″, 5″), 147.8 (C-4′), 147.2 (C-2), 144.9 (C-3′), 140.4 (C-1″), 135.7 (C-3), 126.2 (C-2″, 6″), 121.9 (C-1′), 119.6 (C-6′), 115.7 (C-2′), 115.5 (C-5′), 104.8 (C-8), 103.0 (C-10), 98.2 (C-6) ppm. IR (KBr pellet): 1635 (m), 1598 (m), 1560 (m), 1490 (m), 1200 (s),1133 (m), 1002 (m) cm^−1^.

*8-(5-Pyrimidino)-7-*O*-isopropyl quercetin* (**26**): General method A was followed using 8-iodo-3,3′,4′,5,7-penta-*O*-isopropylquercetin (**5**) and pyrimidine-5-boronic acid, yielding **26** as a yellow solid (50 mg, 40%). HRMS (ESI, *m*/*z*): calcd for C_22_H_17_O_7_N_2_ [M-H]^−^: 421.10412, found: 421.10372. ^1^H NMR (500 MHz, DMSO-*d*_6_) δ 12.91 (s, 1H, HO-5), 9.67 (s, 1H, HO-4′), 9.57 (s, 1H, HO-3), 9.19 (s, 1H, HO-3′), 9.17 (s, 1H, H-4″), 8.93 (s, 2H, H-2″, 6″), 7.47 (d, *J* = 2.2 Hz, 1H, H-2′), 7.08 (dd, *J* = 8.5, 2.2 Hz, 1H, H-6′), 6.74 (d, *J* = 8.5 Hz, 1H, H-5′), 6.72 (s, 1H, H-6), 4.85 (hept, *J* = 6.0 Hz, 1H, -CH), 1.24 (d, *J* = 6.0 Hz, 6H, -CH_3_) ppm. ^13^C NMR (126 MHz, DMSO-*d*_6_) δ 176.2 (C-4), 161.5 (C-7), 160.1 (C-5), 158.4 (C-2″, 6″), 156.9 (C-2″), 152.7 (C-9), 147.8 (C-4′), 147.7 (C-2), 145.0 (C-3′), 135.9 (C-3), 126.4 (C-1″), 121.8 (C-1′), 119.5 (C-6′), 115.6 (C-2′), 115.4 (C-5′), 103.6 (C-8), 102.3 (C-10), 96.1 (C-6), 71.4 (CH), 21.6 (CH_3_) ppm.

*8-(4-Nitrophenyl) quercetin* (**27**): General method A was followed using 8-iodo-3,3′,4′,5,7-penta-*O*-isopropylquercetin (**5**) and 4-nitrophenylboronic acid, affording **27** as a green solid (34 mg, 26%). HRMS (ESI, *m*/*z*): calcd for C_21_H_12_O_9_N [M-H]^−^: 422.05175, found: 422.05107. ^1^H NMR (400 MHz, DMSO-*d*_6_) δ 12.81 (s, 1H, HO-5), 11.09 (s, 1H, HO-7), 9.57 (s, 1H, HO-4′), 9.47 (s, 1H, HO-3), 9.15 (s, 1H, HO-4′), 8.33 (d, *J* = 8.8 Hz, 2H, H-3″, 5″), 7.80 (d, *J* = 8.8 Hz, 2H, H-2″, 6″), 7.42 (d, *J* = 2.2 Hz, 1H, H-2′), 7.19 (dd, *J* = 8.4, 2.2 Hz, 1H, H-6′), 6.75 (d, *J* = 8.5 Hz, 1H, H-5′), 6.43 (s, 1H, H-6) ppm. ^13^C NMR (100 MHz, DMSO-*d*_6_) δ 176.1 (C-4), 160.8 (C-7), 160.5 (C-5), 152.9 (C-9), 147.7 (C-4′), 147.3 (C-2), 146.3 (C-4″), 144.9 (C-3′), 139.6 (C-1″), 135.8 (C-3), 132.5 (C-2″, 6″), 123.0 (C-3″, 5″), 122.0 (C-1′), 119.9 (C-6′), 115.4 (C-2′), 115.3 (C-5′), 105.5 (C-8), 103.2 (C-10), 98.03 (C-6) ppm. IR (CHCl_3_ film): 1653 (m), 1595 (m), 1558 (m), 1514 (m), 1345 (s), 1196 (m), 1022 (m), 999 (s), 699 (m), 680 (m) cm^−1^.

*8-(4-Cyanophenyl) quercetin* (**28**): General method A was followed using 8-iodo-3,3′,4′,5,7-penta-*O*-isopropylquercetin (**5**) and 4-cyanophenylboronic acid, affording **28** as a green solid (50 mg, 40%). HRMS (ESI, *m*/*z*): calcd for C_22_H_12_O_7_N [M-H]^−^: 402.06192, found: 402.06151. ^1^H NMR (500 MHz, DMSO-*d*_6_) δ 12.78 (s, 1H, HO-5), 11.01 (s, 1H, HO-7), 9.60 (s, 1H, HO-4′), 9.46 (s, 1H, HO-3), 9.15 (s, 1H, HO-3′), 7.93 (m, 2H, H-3″, 5″), 7.70 (m, 2H, H-2″, 6″), 7.45 (d, *J* = 2.2 Hz, 1H, H-2′), 7.14 (dd, *J* = 8.5, 2.2 Hz, 1H, H-6′), 6.75 (d, *J* = 8.5 Hz, 1H, H-5′), 6.41 (s, 1H, H-6) ppm. ^13^C NMR (126 MHz, DMSO-*d*_6_) δ 176.1 (C-4), 160.7 (C-7), 160.3 (C-5), 152.9 (C-9), 147.7 (C-4′), 147.3 (C-2), 144.9 (C-3′), 137.5 (C-1″), 135.8 (C-3), 132.2 (C-3″, 5″), 131.7 (C-2″, 6″), 122.0 (C-1′), 119.7 (C-6′), 119.1 (CN), 115.4 (C-2′), 115.4 (C-5′), 109.6 (C-4″), 105.9 (C-8), 103.2 (C-10), 98.0 (C-6) ppm. IR (CHCl_3_ film): 2222 (m), 1655 (m), 1614 (sh), 1594 (s), 1545 (m), 1514 (m), 1354 (s), 1192 (s), 1002 (m) cm^−1^.

*8-(4-Acetylphenyl) quercetin* (**29**): General method A was followed using 8-iodo-3,3′,4′,5,7-penta-*O*-isopropylquercetin (**5**) and 4-acetylphenylboronic acid, affording **29** as a yellow solid (37 mg, 30%). HRMS (ESI, *m*/*z*): calcd for C_23_H_15_O_8_ [M-H]^−^: 419.07724, found: 419.07688. ^1^H NMR (500 MHz, DMSO-*d*_6_) δ 12.76 (s, 1H, HO-5), 10.94 (s, 1H, HO-7), 9.61 (s, 1H, HO-4′), 9.47 (s, 1H, HO-3), 9.14 (s, 1H, HO-3′), 8.09–8.03 (m, 2H, H-3″,5″), 7.67–7.61 (m, 2H, H-2″, 6″), 7.50 (d, *J* = 2.2 Hz, 1H, H-2′), 7.13 (dd, *J* = 8.5, 2.2 Hz, 1H, H-6′), 6.72 (d, *J* = 8.5 Hz, 1H, H-5′), 6.42 (s, 1H, H-6), 2.64 (s, 3H, -CH_3_) ppm. ^13^C NMR (126 MHz, DMSO-*d*_6_) δ 197.8 (C=O), 176.1 (C-4), 160.8 (C-7), 160.0 (C-5), 153.0 (C-9), 147.7 (C-4′), 147.3 (C-2), 145.1 (C-3′), 137.3 (C-4″), 135.8 (C-3), 135.4 (C-1″), 131.5 (C-2″,6″), 127.8 (C-3″, 5″), 122.0 (C-1′), 119.7 (C-6′), 115.7 (C-2′), 115.4 (C-5′), 106.5 (C-8), 103.2 (C-10), 98.1 (C-6), 26.9 (CH_3_). IR (MeOH film): 1671 (sh), 1650 (m), 1624 (sh), 1602 (s), 1560 (m), 1519 (m), 1195 (s), 1022 (m), 838 (m) cm^−1^.

*8-Phenyl luteolin* (**31**): General method A was followed using 8-iodo-3′,4′,5,7-tetra-*O*-isopropylluteolin (**13**) and phenylboronic acid, yielding **31** as a yellow solid (40 mg, 51%). HRMS (ESI, *m*/*z*): calcd for C_21_H_13_O_6_ [M-H]^−^: 361.07176, found: 361.07149. ^1^H NMR (500 MHz, DMSO-*d*_6_) δ 13.19 (s, 1H, HO-5), 10.82 (s, 1H, HO-7), 10.03 (s, 1H, HO-4′), 9.16 (s, 1H, HO-3′), 7.53–7.42 (m, 4H, H-2″, 3″, 5″, 6″), 7.40 (m, 1H, H-4″), 7.09 (d, *J* = 2.3 Hz, 1H, H-2′), 7.06 (dd, *J* = 8.3, 2.3 Hz, 1H, H-6′), 6.74 (d, *J* = 8.3 Hz, 1H, H-5′), 6.68 (s, 1H, H-3), 6.40 (s, 1H, H-6) ppm. ^13^C NMR (126 MHz, DMSO-*d*_6_) δ 182.1 (C-4), 164.2 (C-2), 161.2 (C-7), 160.3 (C-5), 154.1 (C-9), 149.7 (C-4′), 145.6 (C-3′), 132.0 (C-1″), 131.1 (C-2″, 6″), 127.9 (3″, 5″), 121.7 (C-1′), 118.4 (C-6′), 115.6 (C-5′), 113.8 (C-2′), 108.0 (C-8), 103.7 (C-10), 102.6 (C-3), 98.7 (C-6) ppm. IR (MeOH film): 1651 (s), 1610 (m), 1583 (s), 1560 (m), 1520 (m), 836 (s), 701 (m), 686 (m) cm^−1^.

*8-(4-Fluorophenyl) luteolin* (**32**): General method A was followed using 8-iodo-3′,4′,5,7-tetra-*O*-isopropylluteolin (**13**) and 4-fluorophenylboronic acid, yielding **32** as a green solid (70 mg, 70%). HRMS (ESI, *m*/*z*): calcd for C_21_H_14_O_6_F [M + H]^+^: 381.07689, found: 381.07652. ^1^H NMR (500 MHz, DMSO-*d*_6_) δ 13.19 (s, 1H, HO-5), 10.87 (s, 1H, HO-7), 10.01 (s, 1H, HO-4′), 9.22 (s, 1H, HO-3′), 7.54–7.47 (m, 2H, H-2″, 6″), 7.35–7.27 (m, 2H, H-3″, 5″), 7.09 (dd, *J* = 8.3, 2.3 Hz, 1H, H-6′), 7.06 (d, *J* = 2.3 Hz, 1H, H-2′), 6.77 (d, *J* = 8.2 Hz, 1H, H-5′), 6.68 (s, 1H, H-3), 6.40 (s, 1H, H-6) ppm. ^13^C NMR (126 MHz, DMSO-*d*_6_) δ 182.1 (C-4), 164.1 (C-2), 161.35 (d, *J* = 243.7 Hz, C-4″), 161.2 (C-7), 160.4 (C-5), 154.1 (C-9), 149.7 (C-4′), 145.6 (C-3′), 133.1 (d, *J* = 8.3 Hz, C-2″, 6″), 128.2 (C-1″), 121.7 (C-1′), 118.9 (C-6′), 115.7 (C-5′), 114.9 (d, *J* = 21.2 Hz, C-3″, 5″), 113.7 (C-2′), 107.0 (C-8), 103.7 (C-10), 102.7 (C-3), 98.6 (C-6) ppm. IR (MeOH film): 1648 (s), 1601 (m), 1558 (m), 1511 (s), 1228 (m), 1158 (m), 1008 (m), 835 (s), 686 (m) cm^−1^.

*8-(4-Trifluoromethylphenyl) luteolin* (**33**): General method A was followed using 8-iodo-3′,4′,5,7-tetra-*O*-isopropylluteolin (**13**) and 4-(trifluoromethyl)phenylboronic acid, yielding **33** as a green solid (70 mg, 85%). HRMS (ESI, *m*/*z*): calcd for C_22_H_14_O_6_F_3_ [M + H]^+^: 431.07370, found: 431.07386. ^1^H NMR (500 MHz, DMSO-*d*_6_) δ 13.24 (s, 1H, HO-5), 11.02 (s, 1H, HO-7), 9.98 (s, 1H, HO-4′), 9.16 (s, 1H, HO-3′), 7.85 (d, *J* = 7.9 Hz, 1H, H-3″, 5″), 7.73 (d, *J* = 8.6 Hz, 2H, H-2″, 6″), 7.10–7.02 (m, 2H, H-2′, 6′), 6.78–6.72 (m, 1H, H-5′), 6.69 (s, 1H, H-3), 6.42 (s, 1H, H-6) ppm. ^13^C NMR (126 MHz, DMSO-*d*_6_) δ 182.0 (C-4), 164.2 (C-2), 161.1 (C-7), 160.8 (C-5), 154.1 (C-9), 149.7 (C-4′), 145.6 (C-3′), 136.5 (C-1″), 132.0 (C-2″, 6″), 127.6 (q, *J* = 31.8 Hz, C-4″), 124.7 (q, *J* = 3.8 Hz, C-3″, 5″), 124.5 (q, *J*= 271.6Hz, CF_3_), 121.6, 118.7 (C-6′), 115.7 (C-5′), 113.8 (C-2′), 106.5 (C-8), 103.7 (C-10), 102.8 (C-3), 98.6 (C-6) ppm. ^19^F NMR (470 MHz, DMSO-*d*_6_) δ-60.81 ppm. IR (MeOH film): 1649 (s), 1615 (m), 1582 (m), 1556 (m), 1522 (m), 1328 (s), 1193 (s), 1278 (m), 1131 (m), 1009 (m), 835 (s) cm^−1^.

*8-Phenyl chrysin* (**35**): General method A was followed using 8-iodo-5,7-di-*O*-isopropylchrysin and phenylboronic acid, yielding **35** as a white solid (124 mg, 78%). HRMS (ESI, *m*/*z*): calcd for C_21_H_13_O_4_ [M-H]^−^: 329.08193, found: 329.08200. ^1^H NMR (500 MHz, DMSO-*d*_6_) δ 13.02 (s, 1H, HO-5), 10.90 (s, 1H, HO-7), 7.77–7.70 (m, 2H, H-2′, 6′), 7.54–7.38 (m, 8H, H-3′, 4′, 5′, 2″, 3″, 4″, 5″, 6″), 7.01 (s, 1H, H-3), 6.45 (s, 1H, H-6) ppm. ^13^C NMR (126 MHz, DMSO-*d*_6_) δ 182.4 (C-4), 163.1 (C-2), 161.5 (C-7), 160.2 (C-5), 154.1 (C-9), 132.0 (C-4′), 131.9 (C-1″), 131.1 (C-2″, 6″ or C-3″, 5″), 130.8 (C-1′), 129.0 (C-3′, 5′), 127.9 (C-3″, 5″ or C-2″, 6″), 127.2 (C-4″), 126.2 (C-2′, 6′), 108.2 (C-8), 104.8 (C-3), 104.0 (C-10), 98.8 (C-6) ppm.

#### 3.2.2. General Procedure B for Miyaura Borylation

8-Iodo flavonoid (1 eq), Pd(OAc)_2_ (5 mol%) and P(*o*-Tol)_3_ (10 mol%), and triethylamine (3 eq) were dissolved in dry THF in a microwave vessel equipped with a stirring bar. The reaction mixture was degassed with nitrogen and cooled to 0 °C. Pinacolborane (1.1 eq) was added dropwise under an inert atmosphere. The reaction mixture was irradiated in a microwave reactor at 70 °C for 2 h. The reaction mixture was cooled to ambient temperature and evaporated to dryness in vacuo. The residue was purified by FCC (petroleum ether/EtOAc, 3:1) to give the corresponding product.

*8-Boronyl-3,3′,4′,5,7-penta-*O*-isopropylquercetin* (**7**): General method B was followed using 8-iodo-3,3′,4′,5,7-penta-*O*-isopropylquercetin (**5**), yielding **7** as a white solid (489 mg, 98%). HRMS (ESI, *m*/*z*): calcd for C_36_H_52_O_9_B [M + H]^+^: 639.36989, found: 639.36950. ^1^H NMR (400 MHz, DMSO-*d*_6_) δ 7.73 (d, *J* = 2.2 Hz, 1H, H-2′), 7.68 (dd, *J* = 8.6, 2.2 Hz, 1H, H-6′), 7.09 (d, *J* = 8.8 Hz, 1H, H-5′), 6.58 (s, 1H, H-6), 4.80 (m, 2H, CH-*i*Pr), 4.67 (m, 1H, CH-*i*Pr), 4.58 (m, 1H, CH-*i*Pr), 4.53–4.42 (m, 1H, CH-*i*Pr), 1.36–1.24 (m, 36H, CH3- *i*Pr, boronate), 1.09 (d, *J* = 6.3 Hz, 6H, CH_3_-*i*Pr) ppm. ^13^C NMR (100 MHz, DMSO-*d*_6_) δ 172.6 (C-4), 165.1 (C-7), 160.6 (C-5), 160.2 (C-2), 152.2 (C-9), 150.6 (C-4′), 146.8 (C-3′), 137.5 (C-3), 123.4 (C-6′), 122.7 (C-1′), 119.3 (C-2′), 115.0 (C-5′), 109.0 (C-10), 97.0 (C-6), 83.5 (C_q_-boronate), 72.8 (*i*Pr-CH), 72.0 (CH-*i*Pr), 71.4 (CH-*i*Pr), 70.6 (CH-*i*Pr), 70.5 (CH-*i*Pr), 24.7 (CH_3_-boronate), 22.0 (CH_3_-*i*Pr), 22.0 (CH_3_-*i*Pr), 21.8 (CH_3_-*i*Pr), 21.7 (CH_3_-*i*Pr) ppm. IR (CHCl_3_ film): 2977 (s), 2933 (m), 2874 (m),1639 (m), 1624 (sh), 1588 (m), 1566 (m), 1504 (m), 1397 (m), 1381 (m), 1372 (m), 1317 (m), 1194 (s), 1008 (s), 1005 (m), 960 (m) cm^−1^.

*8-Boronyl-3′,4′,5,7-tetra-*O*-isopropylluteolin* (**12**): General method B was followed using 8-iodo-3′,4′,5,7-tetra-*O*-isopropylluteolin (**13**), yielding **12** as a white solid (489 mg, 98%). HRMS (ESI, *m*/*z*): calcd for C_33_H_46_O_8_B [M + H]^+^: 581.32803, found: 581.32714. ^1^H NMR (500 MHz, DMSO-*d*_6_) δ 7.62 (dd, *J* = 8.6, 2.3 Hz, 1H, H-6′), 7.53 (d, *J* = 2.3 Hz, 1H, H-2′), 7.11 (d, *J* = 8.7 Hz, 1H, H-5′), 6.59 (s, 2H, H-3, H-6), 4.82 (m, 1H, CH-*i*Pr), 4.77 (m, 1H, CH-*i*Pr), 4.73–4.65 (m, 1H, CH-*i*Pr), 4.62 (m, 1H, CH-*i*Pr), 1.36 (s, 12H, CH_3_-boronate), 1.32 (d, *J* = 6.0 Hz, 6H, CH_3_-*i*Pr), 1.28 (m, 12H, CH_3_-*i*Pr), 1.23 (d, *J* = 6.1 Hz, 6H, CH_3_-*i*Pr) ppm. ^13^C NMR (126 MHz, DMSO-*d*_6_) δ 175.7 (C-4), 165.2 (C-2), 161.2 (C-7), 160.4 (C-5), 159.6 (C-9), 151.9 (C-4′), 147.8 (C-3′), 123.7 (C-1′), 120.8 (C-6′), 116.9 (C-2′), 115.4 (C-5′), 109.0 (C-10), 107.1 (C-3), 97.6 (C-6), 83.5 (CH-*i*Pr), 73.5 (CH-*i*Pr), 72.2 (CH-*i*Pr), 71.4 (CH-*i*Pr), 24.8 (CH_3_-boronate), 22.0 (CH_3_-*i*Pr), 21.8 (CH_3_-*i*Pr), 21.7 (CH_3_-*i*Pr) ppm. IR (CHCl_3_ film): 2978 (s), 2933 (m), 2871 (m), 1642 (m), 1590 (m), 1566 (m), 1506 (m), 1381 (m), 1373 (m), 1315 (m), 1272 (m), 1006 (s), 1019 (m), 951 (m) cm^−1^.

#### 3.2.3. General Procedure C for Alkylation of Per-Isopropyl Derivatives

8-Boronyl flavonoid (1 eq), K_2_CO_3_ (2 eq), and Pd(OAc)_2_ (5 mol%) were dissolved in dry toluene. Alkyl bromide (1.5 eq) was added under an inert atmosphere. The reaction mixture was heated and stirred at 90 °C for 2 days. The reaction mixture was cooled to ambient temperature and evaporated in vacuo. The residue was dissolved in dry dichloromethane (5 mL) and cooled to −10 °C. Boron trichloride solution (10 eq, 1 M solution in dichloromethane) was added dropwise and the reaction mixture was stirred at −10 °C for 30 min. The reaction mixture was heated to 40 °C and stirred for 2 h. The reaction mixture was cooled to 0 °C and an excess of methanol was added. The reaction mixture was evaporated in vacuo, and the residue was purified by a preparative HPLC system (ASAHIPACK, 5 mL/min isocratic) to give the corresponding product.

*8-Allyl quercetin* (**9**): General method C was followed using 8-boronyl-3,3′,4′,5,7-penta-*O*-isopropylquercetin (**7**) and allyl bromide, affording **9** as a yellow solid (54 mg, 51%). HRMS (ESI, *m*/*z*): calcd for C_18_H_13_O_7_ [M-H]^−^: 341.06668, found: 341.06653. ^1^H NMR (500 MHz, DMSO-*d*_6_) δ 12.44 (s, 1H, HO-5), 10.74 (s, 1H, HO-7), 9.60 (s, 1H, HO-4′), 9.36 (br. s, 2H, HO-3, HO-3′), 7.71 (d, *J* = 2.2 Hz, 1H, H-2′), 7.56 (dd, *J* = 8.5, 2.2 Hz, 1H, H-6′), 6.89 (d, *J* = 8.5 Hz, 1H, H-5′), 6.30 (s, 1H, H-6), 6.00–5.89 (m, 1H, H-2″), 5.11–4.88 (m, 2H, H-3″), 3.49 (d, *J* = 5.9 Hz, 2H, H-1″) ppm. ^13^C NMR (126 MHz, DMSO-*d*_6_) δ 176.1 (C-4), 161.3 (C-7), 158.6 (C-5), 153.5 (C-9), 147.7 (C-4′), 146.7 (C-3), 145.2 (C-3′), 135.9 (C-2″), 135.6 (C-3), 122.3 (C-1′), 119.9 (C-6′), 115.7 (C-5′), 115.0 (C-2′), 114.8 (C-3″), 103.6 (C-8), 103.0 (C-10), 97.7 (C-6), 26.3 (C-1″) ppm. IR (MeOH film): 1653 (s), 1618 (m), 1555 (m), 1518 (m), 1320 (s), 1244 (s), 1159 (m), 1003 (m) cm^−1^.

*8,7-(2,2-Dimethyldihydropyrano) quercetin (sinoflavonoid NB)* (**11a**) and *7,6-(2,2-dimethyldihydropyrano)quercetin* (**11b**): General method C was followed using 8-boronyl-3,3′,4′,5,7-penta-*O*-isopropylquercetin (**7**) and prenyl bromide, affording a mixture of **11a** and **11b** as a yellow solid (22 mg, 22%). HRMS (ESI, *m*/*z*): calcd for C_20_H_17_O_7_ [M-H]^−^: 369.09798, found: 369.09768. ^1^H NMR (500 MHz, DMSO) δ 10.77 (s, 1H, HO-5), 7.77 (d, *J* = 2.2 Hz, 1H, H-2′ (11b)), 7.74 (d, *J* = 2.2 Hz, 1H, H-2′, (11a)), 7.61 (dd, *J* = 8.5, 2.2 Hz, 1H, H-6′, (11a)), 7.56 (dd, *J* = 8.5, 2.2 Hz, 1H, H-6′, (11b)), 6.90 (d, *J* = 8.5 Hz, 1H, H-5′, (11a)), 6.85 (d, *J* = 8.4 Hz, 1H, H-5′, (11b)), 6.31 (s, 1H, H-8, (11b)), 6.13 (s, 1H, H-6, (11a)), 2.96–2.89 (m, 2H, H-1″, (11b)), 2.85 (t, *J* = 6.7 Hz, 2H, H-1″, (11a)), 1.95–1.90 (m, 1H, H-2″, (11b)), 1.87 (t, *J* = 6.7 Hz, 2H, H-2″, (11a)), 1.65 (s, 6H, CH_3_, (11b)), 1.33 (s, 6H, CH_3_, (11a)) ppm. ^13^C NMR (126 MHz, DMSO) δ 176.1 (C-4, (11a)), 176.0 (C-4, (11b)), 161.4 (C-7, (11b)), 159.2 (C-5, (11a)), 158.4 (C-9, (11b)), 158.0 (C-9, (11a)), 153.5 (C-5, (11b)), 153.0 (C-5, (11a)), 147.8 (C-4′, (11a)), 147.7 (C-4′, (11b)), 146.7 (C-2, (11b)), 145.2 (C-3′, (11a)), 145.1 (C-3′, (11b)), 136.1 (C-3, (11a)), 135.6 (C-3, (11b)), 122.3 (C-1′, (11b)), 122.2 (C-1′, (11a)), 120.0 (C-6′, (11a)), 119.6 (C-6′, (11b)), 115.7 (C-2′, (11a)), 115.5 (C-2′, (11b)), 115.4 (C-5′, (11b)), 114.9 (C-5′, (11a)), 105.2 (C-6, (11b)), 103.6 (C-10, (11a)), 103.0 (C-10, (11b)), 99.7 (C-8, (11a)), 98.6 (C-6, (11a)), 97.7 (C-8, (11b)), 76.2 (C-3″′, (11a)), 72.2 (C-3″′, (11b)), 44.4 (C-2″′, (11b)), 32.1 (CH_3_, (11b)), 31.0 (C-2″′, (11a)), 26.3 (CH_3_, (11a)), 18.2 (C-1″′, (11b)), 15.7 (C-1″′, (11a)) ppm.

*6,7-(2,2-Dimethyldihydropyrano) quercetin* (**11b**): General method C was followed using 8-boronyl-3,3′,4′,5,7-penta-*O*-isopropylquercetin (**7**) and prenyl bromide, affording 8-prenyl-3,3′,4′,5,7-penta-*O*-isopropylquercetin quercetin (**10**), which was deprotected using boron trichloride at room temperature yielding **11b** as a yellow solid (42 mg, 26%). HRMS (ESI, *m*/*z*): calcd for C_20_H_17_O_7_ [M-H]^−^: 369.09798, found: 369.09768. ^1^H NMR (500 MHz, DMSO-*d*_6_) δ 7.77 (d, *J* = 2.2 Hz, 1H, H-2′), 7.56 (dd, *J* = 8.5, 2.2 Hz, 1H, H-6′), 6.85 (d, *J* = 8.5 Hz, 1H, H-5′), 6.30 (s, 1H, H-8), 2.96–2.90 (m, 2H, H-1″), 1.95–1.90 (m, 2H, H-2″), 1.66 (s, 6H, CH_3_) ppm. ^13^C NMR (126 MHz, DMSO-*d*_6_) δ 176.1 (C-4), 161.4 (C-7), 158.4 (C-9), 153.5 (C-5), 147.7 (C-4′), 146.7 (C-2), 145.1 (C-3′), 135.5 (C-3), 122.3 (C-1′), 119.6 (C-6′), 115.5 (C-2′), 115.4 (C-5′), 105.2 (C-6), 103.0 (C-10), 97.7 (C-8), 72.2 (C-3″), 44.4 (C-2″), 32.1 (CH_3_), 18.2 (C-1″) ppm.

#### 3.2.4. General Procedure D for the Preparation of Hexahydroxy Flavonoids

8-Boronyl flavonoid (1 eq) and sodium perborate tetrahydrate (5 eq) were dissolved in a THF/H_2_O mixture (1:1). The reaction mixture was stirred at room temperature for 3 h, quenched with water, and extracted with dichloromethane (3 × 10 mL). The combined organic layers were washed with water (3 × 10 mL) and brine (3 × 10 mL), dried over Na_2_SO_4_, and evaporated to dryness in vacuo. The residue was dissolved in dry dichloromethane (3 mL) and cooled to −10 °C. BCl_3_ solution (10 eq, 1 M in dichloromethane) was added dropwise, and the reaction mixture was stirred at −10 °C for 30 min. The reaction mixture was heated to 40 °C and stirred for 2 h and then cooled to 0 °C, and an excess of methanol was added. The reaction mixture was evaporated in vacuo, and the residue was purified using a preparative HPLC system (ASAHIPACK, 5 mL/min isocratic), affording the desired product.

*8-Hydroxy quercetin (gossypetin)* (**30**): General method D was followed using 8-boronyl-3,3′,4′,5,7-penta-*O*-isopropylquercetin (**7**), yielding **30** as a yellow solid (80 mg, 82%). HRMS (ESI, *m*/*z*): calcd for C_15_H_11_O_8_ [M + H]^+^: 319.04484, found: 319.04480. ^1^H NMR (500 MHz, DMSO-*d*_6_) δ 11.93 (bs, 1H, HO-5), 10.43 (bs, 1H, HO-7), 9.57 (bs, 1H, HO-4′), 9.29 (bs, 2H, H-3, 3′), 8.60 (bs, 1H, HO-8), 7.77 (d, *J* = 2.2 Hz, 1H, H-2′), 7.65 (dd, *J* = 8.5, 2.2 Hz, 1H, H-6′), 6.89 (d, *J* = 8.5 Hz, 1H, H-5′), 6.26 (s, 1H, H-6) ppm. ^13^C NMR (126 MHz, DMSO-*d*_6_) δ 176.1 (C-4), 152.7 (C-7), 152.2 (C-5), 147.7 (C-4′), 146.8 (C-2), 145.2 (C-9), 144.9 (C-3′), 135.8 (C-3), 124.7 (C-8), 122.3 (C-1′), 120.6 (C-6′), 115.5 (C-2′), 115.3 (C-5′), 102.8 (C-10), 98.2 (C-6) ppm. IR (MeOH film): 1656 (m), 1614 (m), 1567 (s), 1523 (s), 1324 (s), 1191 (s), 1130 (m), 1004 (m) cm^−1^.

*8-Hydroxy luteolin (hypolaetin)* (**34**): General method D was followed using 8-boronyl-3′,4′,5,7-tetra-*O*-isopropylluteolin (**12**), affording **34** as a yellow solid (56.1 mg, 60%). HRMS (ESI, *m*/*z*): calcd for C_15_H_11_O_7_ [M + H]^+^: 303.04993, found: 303.05030. ^1^H NMR (500 MHz, DMSO-*d*_6_) δ 12.40 (s, 1H, HO-5), 10.51 (s, 1H, HO-7), 9.89 (s, 1H, HO-4′), 9.42 (s, 1H, HO-3′), 8.72 (s, 1H, HO-8), 7.51 (d, *J* = 2.2 Hz, 1H, H-2′), 7.49 (dd, *J* = 8.3, 2.2 Hz, 1H, H-6′), 6.89 (d, *J* = 8.3 Hz, 1H, H-5′), 6.63 (s, 1H, H-3), 6.26 (s, 1H, H-6) ppm. ^13^C NMR (126 MHz, DMSO-*d*_6_) δ 182.0 (C-4), 163.8 (C-2), 153.3 (C-7), 153.0 (C-5), 149.7 (C-4′), 145.7 (C-9), 145.5 (C-3′), 125.1 (C-8), 121.8 (C-1′), 119.1 (C-6′), 115.9 (C-5′), 113.6 (C-2′), 103.3 (C-10), 102.4 (C-3), 98.6 (C-6) ppm. IR (MeOH film): 1660 (m), 1587 (s), 1519 (s), 1362 (s), 1193 (s), 1123 (m), 1012 (m) cm^−1^.

#### 3.2.5. General Procedure E for the Preparation of Biflavonoids

Halogen flavone (1 eq) and 8-boronyl flavonoid (1.5 eq) were dissolved in dry dioxane and degassed with nitrogen. Pd(PPh_3_)_4_ (10 mol%) and Cs_2_CO_3_ (2 eq) were added under an inert atmosphere. The reaction mixture was heated and stirred at 100 °C for 3 days. The reaction mixture was cooled to ambient temperature and evaporated in vacuo. The residue was dissolved in dry dichloromethane (5 mL) and cooled to −10 °C. BCl_3_ solution (10 eq, 1 M solution in dichloromethane) was added dropwise, and the reaction mixture was stirred at −10 °C for 30 min. The reaction mixture was heated to 40 °C and stirred for 2 h. The reaction mixture was cooled to 0 °C, and an excess of methanol was added. The reaction mixture was evaporated in vacuo, and the residue was purified by a preparative HPLC system (ASAHIPACK, 5 mL/min isocratic), yielding the corresponding product.

*(Flavone)-(8→8)-(3,5,7,*3′,4′-*pentahydroxyflavone)* (**18**): General method E was followed using 8-boronyl-3,3′,4′,5,7-penta-*O*-isopropylquercetin (**7**) and 6-bromoflavone (**17**), yielding **18** as a yellow solid (117 mg, 70%). HRMS (ESI, *m*/*z*): calcd for C_30_H_19_O_9_ [M + H]^+^: 523.10236, found: 523.10250. ^1^H NMR (500 MHz, DMSO-*d*_6_) δ 12.76 (s, 1H, HO-5), 10.96 (s, 1H, HO-7), 9.53 (bs, 1H, HO-4′), 9.46 (s, 1H, HO-3), 9.08 (bs, 1H, HO-3′), 8.17 (m, 3H, H-2″′, 6″′, 5″), 7.97 (dd, *J* = 8.6, 2.2 Hz, 1H, H-7″), 7.90 (d, *J* = 8.6 Hz, 1H, H-8″), 7.68–7.58 (m, 3H, H-3″′, 4″′, 5″′), 7.51 (d, *J* = 2.2 Hz, 1H, H-2′), 7.16 (dd, *J* = 8.5, 2.2 Hz, 1H, H-6′), 7.12 (s, 1H, H-3″), 6.67 (d, *J* = 8.5 Hz, 1H, H-5′), 6.45 (s, 1H, H-6) ppm. ^13^C NMR (126 MHz, DMSO-*d*_6_) δ 177.1 (C-4″), 176.1 (C-4), 162.6 (C-2″), 160.8 (C-7), 159.9 (C-5), 154.8 (C-9″), 153.0 (C-9), 147.6 (C-4′), 147.2 (C-2), 144.8 (C-3′), 137.3 (C-7″), 135.8 (C-3), 131.9 (C-4″′), 131.2 (C-6″), 129.3 (C-1″′), 129.2 (C-3″′, 5″′), 127.1 (C-5″), 126.4 (C-2″′, 6″′), 123.0 (C-10″), 122.0 (C-1′), 119.6 (C-6′), 118.1 (C-8″), 115.5 (C-2′), 115.4 (C-5′), 107.0 (C-3″), 105.8 (C-8), 103.2 (C-10), 98.1 (C-6) ppm. IR (MeOH film): 1660(m), 163 1(s), 1614 (s), 1602 (s), 1563 (s), 1523 (m), 1458 (m), 1273 (s), 1184 (m), 1127 (m), 1001 (m) cm^−1^.

*(Flavone)-(8→8)-(5,7,*3′,4′-*tetraahydroxyflavone)* (**19**): General method E was followed using 8-boronyl-3′,4′,5,7-tetra-*O*-isopropylluteolin (**12**) and 6-bromoflavone (**17**), affording **19** as a yellow solid (121 mg, 70%). HRMS (ESI, *m*/*z*): calcd for C_30_H_19_O_8_ [M + H]^+^: 507.10744, found: 507.10763. ^1^H NMR (500 MHz, DMSO-*d*_6_) δ 13.27 (bs, 1H, HO-5), 11.03 (s, 1H, HO-7), 9.90 (bs, 1H, HO-4′), 9.16 (s, 1H, HO-3′), 8.23–8.12 (m, 3H, H-2″′, 6″′, 5″), 7.98 (dd, *J* = 8.6, 2.2 Hz, 1H, H-7″), 7.92 (d, *J* = 8.6 Hz, 1H, H-8″), 7.68–7.58 (m, 3H, H-3″′, 4″′, 5″′), 7.17–7.10 (m, 2H, H-6′, 3″), 7.06 (d, *J* = 2.3 Hz, 1H, H-2′), 6.73 (d, *J* = 8.4 Hz, 1H, H-5′), 6.71 (s, 1H, H-3), 6.45 (s, 1H, H-6) ppm. ^13^C NMR (126 MHz, DMSO-*d*_6_) δ 182.1 (C-4), 177.1 (C-4″), 164.1 (C-2), 162.6 (C-2″), 161.2 (C-7), 160.7 (C-5), 154.8 (C-9″), 154.1 (C-9), 149.7 (C-4′), 145.6 (C-3′), 137.2 (C-7″), 131.9 (C-4″′), 131.2 (C-6″), 129.22 (C-3″′, 5″′), 129.17 (C-1″′), 127.0 (C-5″), 126.4 (C-2″′, 6″′), 123.0 (C-10″), 121.5 (C-1′), 118.9 (C-6′), 118.2 (C-8″), 115.8 (C-5′), 113.6 (C-2′), 107.0 (C-3″), 106.3 (C-8), 103.8 (C-10), 102.8 (C-3), 98.7 (C-6) ppm. IR (MeOH film): 1656 (m), 1636 (s), 1602 (m), 1563 (m), 1523 (m), 1452 (s), 1279 (s), 1230 (m), 1127 (m), 963 (m) cm^−1^.

## 4. Conclusions

The reactivity of halogen derivatives of flavonoids and their boronic acid pinacol esters in the Suzuki–Miyaura cross-coupling reaction was investigated. The reactivity of these derivatives depends strongly on the substitution of HO-7 and the reactivity of the boronate used. In the case of biflavonoid preparation, the reactivity was affected by the substitution on the A-ring of flavonoid. Optimized reaction conditions were used for the preparation of new synthetic derivatives of quercetin (**1**), luteolin (**2**), and chrysin (**3**), as well as flavonoid boronates. This method was also used for the first reported synthesis of sinoflavonoids (**11a**, **11b**). Unsymmetrical biflavonoids connecting flavonoid-flavone (**18**, **19**) via C-8 were prepared. Boronyl flavonoids are also useful intermediates for the introduction of a new hydroxyl group at C-8, resulting in gossypetin (**30**) and hypolaetin (**34**) (naturally occurring flavonoids). All derivatives prepared have been fully characterized and are currently being tested for their biological activity.

## Figures and Tables

**Table 1:** Optimization of reaction conditions for the reaction of 8-halogen quercetin with 3-thienylboronic acid.

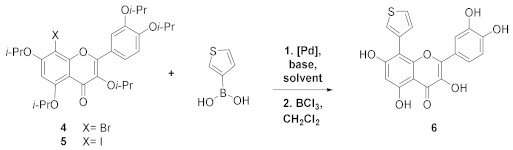							
**Entry**	**Solvent**	**Base**	**Catalyst**	**T [°C]**	**Time [h]**	**X**	**Yield [%]**
1	DMF/H_2_O	NaOH	Pd(PPh_3_)_4_	80	16	Br	18
2	toluene	K_3_PO_4_	Pd(OAc)_2_	80	16	Br	0
3	DMF	K_2_CO_3_	Pd(PPh_3_)_4_	100	16	Br	0
4	dioxane	Cs_2_CO_3_	Pd_2_(dba)_3_ +PPh_3_	80	16	I	10
5	dioxane	Cs_2_CO_3_	Pd_2_(dba)_3_ +P(o-tol)_3_	80	16	I	24
6	dioxane	Cs_2_CO_3_	Pd_2_(dba)_3_ +P(o-tol)_3_	120 ^a^	2	I	50
7	DMF/H_2_O	NaOH	Pd(PPh_3_)_4_	120 ^a^	2	I	98
8	THF	KOH	PEPPSI-iPr	70	16	I	98

^a^ reaction was carried out in a microwave reactor.

## Data Availability

Data archived in the author’s institution.

## Supplementary Materials

The following supporting information can be downloaded online. Experimental procedures, spectral data, ^1^H and ^13^C NMR spectra for products.

## Sample Availability

Samples of the prepared compounds are available from the authors upon request.
