# Supplementary material for: Preparation of Synthetic and Natural Derivatives of Flavonoids Using Suzuki–Miyaura Cross-Coupling Reaction
Source: Molecules. 2022 Jan 31;27(3):967. doi: 10.3390/molecules27030967 (PMC8840526; doi:10.3390/molecules27030967)
Supplement: Supplementary file 1 [file molecules-27-00967-s001.zip › molecules-1574461-supplementary.pdf]

molecules

MDPI

Supplementary Materials

Preparation of Synthetic and Natural Derivatives of Flavonoids Using Suzuki–Miyaura Cross-Coupling Reaction

Martina Hurtová [1,2], David Biedermann [1], Zuzana Osifová [3], Josef Cvačka [3], Kateřina Valentová [1] and Vladimír Křen [1,*]

1 Institute of Microbiology of the Czech Academy of Sciences, Laboratory of Biotransformation, Vídeňská 1083, CZ-14220 Prague, Czech Republic; martina.hurtova@biomed.cas.cz (M.H.); david.biedermann@gmail.com (D.B.); kata.valentova@email.cz (K.V.)
2 Faculty of Food and Biochemical Technology, University of Chemistry and Technology Prague, Technická 5, CZ-16628 Prague, Czech Republic
3 Institute of Organic Chemistry and Biochemistry of the Czech Academy of Sciences, Flemingovo nám. 542/2, CZ-16000 Prague, Czech Republic; zuzana.osifova@uochb.cas.cz (Z.O.); josef.cvacka@uochb.cas.cz (J.C.)
* Correspondence: kren@biomed.cas.cz

Contents

NMR Spectra....4

Figure S1: ^{1}H NMR (500 MHz, DMSO-d_6, 25 °C) spectrum of **6**....4

Figure S2: ^{13}C APT NMR (126 MHz, DMSO-d_6, 25 °C) spectrum of **6**....5

Figure S3: ^{1}H NMR (400 MHz, DMSO-d_6, 25 °C) spectrum of **20**....5

Figure S4: ^{13}C NMR (100 MHz, DMSO-d_6, 25 °C) spectrum of **20**....6

Figure S5: ^{1}H NMR (400 MHz, DMSO-d_6, 25 °C) spectrum of **21**....6

Figure S6: ^{13}C NMR (100 MHz, DMSO-d_6, 25 °C) spectrum of **21**....7

Figure S7: ^{19}F NMR (376 MHz, DMSO-d_6, 25 °C) spectrum of **21**....7

Figure S8: ^{1}H NMR (500 MHz, DMSO-d_6, 25 °C) spectrum of **22**....8

Figure S9: ^{13}C APT NMR (126 MHz, DMSO-d_6, 25 °C) spectrum of **22**....8

Figure S10: ^{1}H NMR (500 MHz, DMSO-d_6, 25 °C) spectrum of **23**....9

Figure S11: ^{13}C APT NMR (126 MHz, DMSO-d_6, 25 °C) spectrum of **23**....9

Figure S12: ^{19}F NMR (470 MHz, DMSO-d_6, 25 °C) spectrum of **23**....10

Figure S13: ^{1}H NMR (400 MHz, DMSO-d_6, 25 °C) spectrum of **24**....10

Figure S14: ^{13}C NMR (101 MHz, DMSO-d_6, 25 °C) spectrum of **24**....11

Figure S15: ^{1}H NMR (500 MHz, DMSO-d_6, 25 °C) spectrum of **25**....11

Figure S16: ^{13}C APT NMR (126 MHz, DMSO-d_6, 25 °C) spectrum of **25**....12

Figure S17: ^{1}H NMR (500 MHz, DMSO-d_6, 25 °C) spectrum of **26**....12

Figure S18: ^{13}C APT NMR (126 MHz, DMSO-d_6, 25 °C) spectrum of **26**....13

Figure S19: ^{1}H NMR (500 MHz, DMSO-d_6, 25 °C) spectrum of **27**....13

Figure S20: ^{13}C NMR (101 MHz, DMSO-d_6, 25 °C) spectrum of **27**.....14
Figure S21: ^{1}H NMR (500 MHz, DMSO-d_6, 25 °C) spectrum of **28**.....14
Figure S22: ^{13}C APT NMR (126 MHz, DMSO-d_6, 25 °C) spectrum of **28**.....15
Figure S23: ^{1}H NMR (500 MHz, DMSO-d_6, 25 °C) spectrum of **29**.....15
Figure S24: ^{13}C APT NMR (126 MHz, DMSO-d_6, 25 °C) spectrum of **29**.....16
Figure S25: ^{1}H NMR (400 MHz, DMSO-d_6, 25 °C) spectrum of **7**.....16
Figure S26: ^{13}C NMR (101 MHz, DMSO-d_6, 25 °C) spectrum of **7**.....17
Figure S27: ^{1}H NMR (500 MHz, DMSO-d_6, 25 °C) spectrum of **9**.....17
Figure S28: ^{13}C APT NMR (126 MHz, DMSO-d_6, 25 °C) spectrum of **9**.....18
Figure S29: ^{1}H NMR (500 MHz, DMSO-d_6, 25 °C) spectrum of **10**.....18
Figure S30: ^{13}C APT NMR (126 MHz, DMSO-d_6, 25 °C) spectrum of **10**.....19
Figure S31: ^{1}H NMR (500 MHz, DMSO-d_6, 25 °C) spectrum of **11b**.....19
Figure S32: ^{13}C NMR (126 MHz, DMSO-d_6, 25 °C) spectrum of **11b**.....20
Figure S33: ^{1}H NMR (500 MHz, DMSO-d_6, 25 °C) spectrum of **30**.....20
Figure S34: ^{13}C APT NMR (126 MHz, DMSO-d_6, 25 °C) spectrum of **30**.....21
Figure S35: ^{1}H NMR (500 MHz, DMSO-d_6, 25 °C) spectrum of **31**.....21
Figure S36: ^{13}C APT NMR (126 MHz, DMSO-d_6, 25 °C) spectrum of **31**.....22
Figure S37: ^{1}H NMR (500 MHz, DMSO-d_6, 25 °C) spectrum of **32**.....22
Figure S38: ^{13}C APT NMR (126 MHz, DMSO-d_6, 25 °C) spectrum of **32**.....23
Figure S39: ^{19}F NMR (470 MHz, DMSO-d_6, 25 °C) spectrum of **32**.....23
Figure S40: ^{1}H NMR (500 MHz, DMSO-d_6, 25 °C) spectrum of **33**.....24
Figure S41: ^{13}C APT NMR (126 MHz, DMSO-d_6, 25 °C) spectrum of **33**.....24
Figure S42: ^{19}F NMR (470 MHz, DMSO-d_6, 25 °C) spectrum of **33**.....25
Figure S43: ^{1}H NMR (500 MHz, DMSO-d_6, 25 °C) spectrum of **12**.....25
Figure S44: ^{13}C APT NMR (126 MHz, DMSO-d_6, 25 °C) spectrum of **12**.....26
Figure S45: ^{1}H NMR (500 MHz, DMSO-d_6, 25 °C) spectrum of **34**.....26
Figure S46: ^{13}C APT NMR (126 MHz, DMSO-d_6, 25 °C) spectrum of **34**.....27
Figure S47: ^{1}H NMR (500 MHz, DMSO-d_6, 25 °C) spectrum of **35**.....27
Figure S48: ^{13}C APT NMR (126 MHz, DMSO-d_6, 25 °C) spectrum of **35**.....28
Figure S49: ^{1}H NMR (500 MHz, DMSO-d_6, 25 °C) spectrum of 1**8**.....28
Figure S50: ^{13}C APT NMR (126 MHz, DMSO-d_6, 25 °C) spectrum of **18**.....29

Figure S51: ^{1}H NMR (500 MHz, DMSO-*d*6, 25 °C) spectrum of **19**. 29
Figure S52: ^{13}C APT NMR (126 MHz, DMSO-*d*6, 25 °C) spectrum of **19**. 30
IR Spectra 30
Figure S53: IR (MeOH film) spectrum of **6**. 30
Figure S54: IR (MeOH film) spectrum of **20**. 31
Figure S55: IR (MeOH film) spectrum of **21**. 31
Figure S56: IR (KBr pellet) spectrum of **22**. 32
Figure S57: IR (MeOH film) spectrum of **23**. 32
Figure S58: IR (MeOH film) spectrum of **24**. 33
Figure S59: IR (KBr pellet) spectrum of **25**. 33
Figure S60: IR ($CHCl_3$ film) spectrum of **27**. 34
Figure S61: IR ($CHCl_3$ film) spectrum of **28**. 34
Figure S62: IR (MeOH film) spectrum of **29**. 35
Figure S63: IR (MeOH film) spectrum of **31**. 35
Figure S64: IR (MeOH film) spectrum of **32**. 36
Figure S65: IR (MeOH film) spectrum of **33**. 36
Figure S66: IR ($CHCl_3$ film) spectrum of **7**. 37
Figure S67: IR ($CHCl_3$ film) spectrum of **12**. 37
Figure S68: IR (MeOH film) spectrum of **9**. 38
Figure S69: IR (MeOH film) spectrum of **30**. 38
Figure S70: IR (MeOH film) spectrum of **34**. 39
Figure S71: IR (MeOH film) spectrum of **18**. 39
Figure S72: IR (MeOH film) spectrum of **19**. 40

NMR Spectra

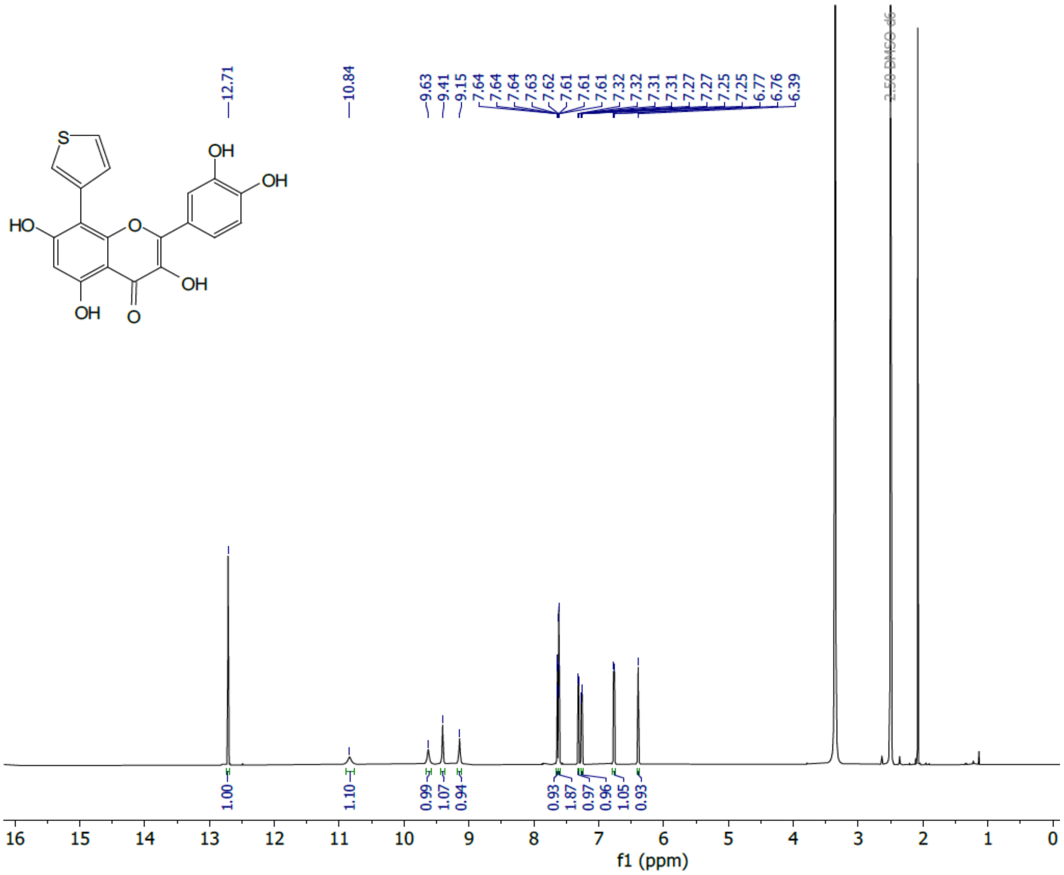

Figure S1: ^{1}H NMR (500 MHz, DMSO-*d*6, 25 °C) spectrum of **6**.

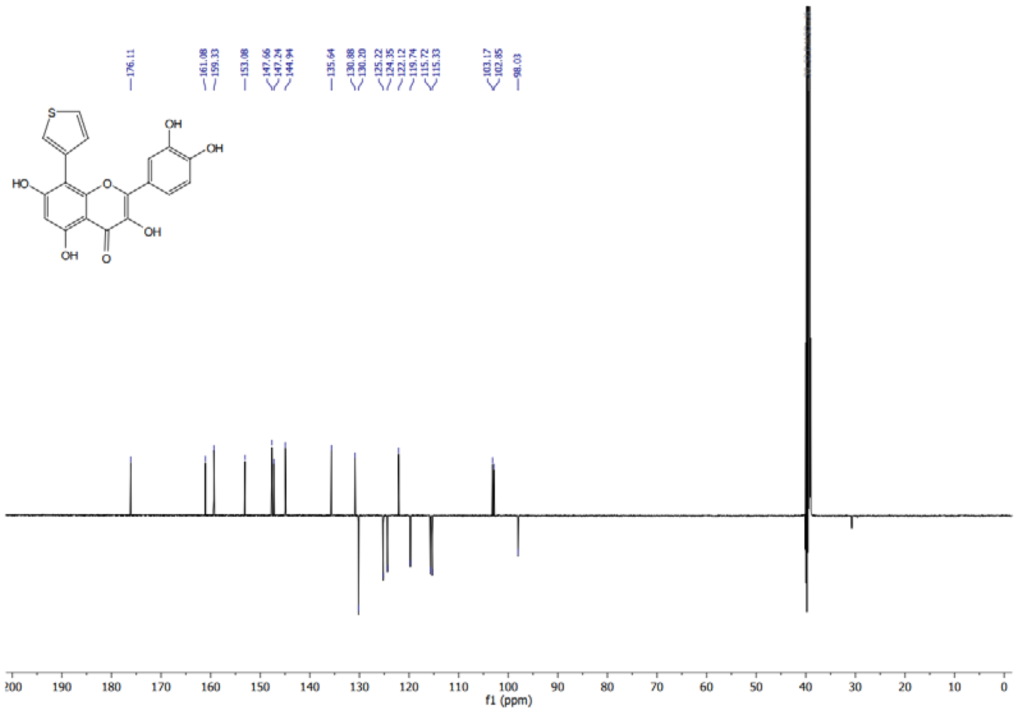

Figure S2: ^{13}C APT NMR (126 MHz, DMSO-*d*$_6$, 25 °C) spectrum of **6**.

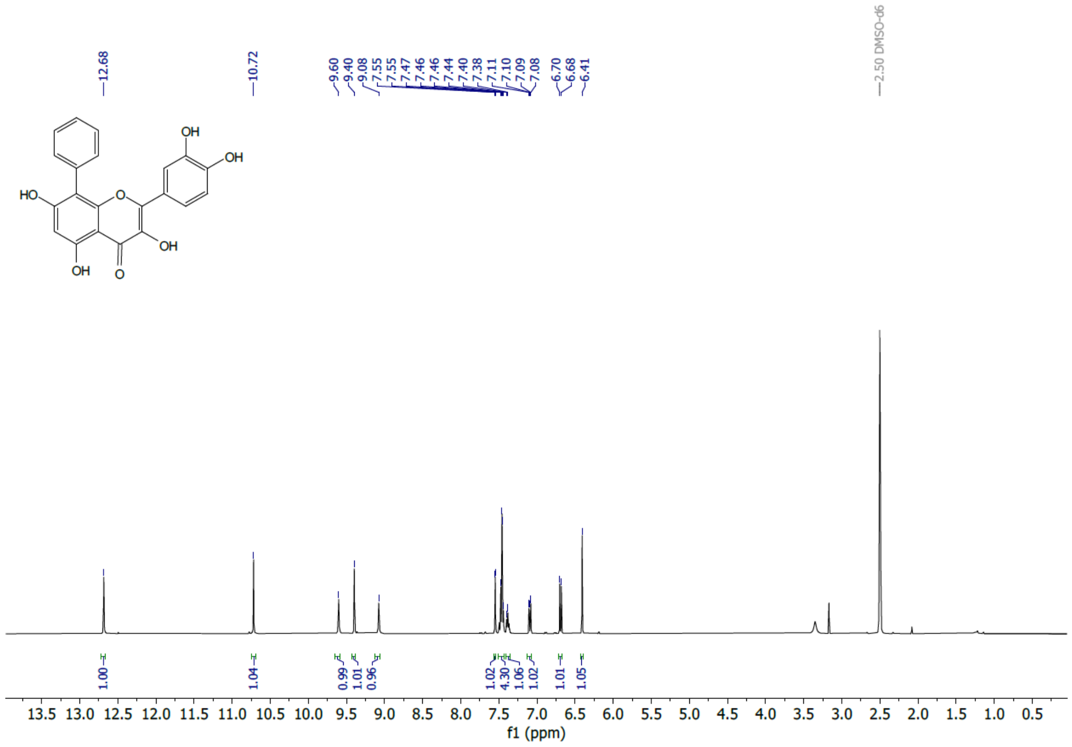

Figure S3: ^{1}H NMR (400 MHz, DMSO-*d*$_6$, 25 °C) spectrum of **20**.

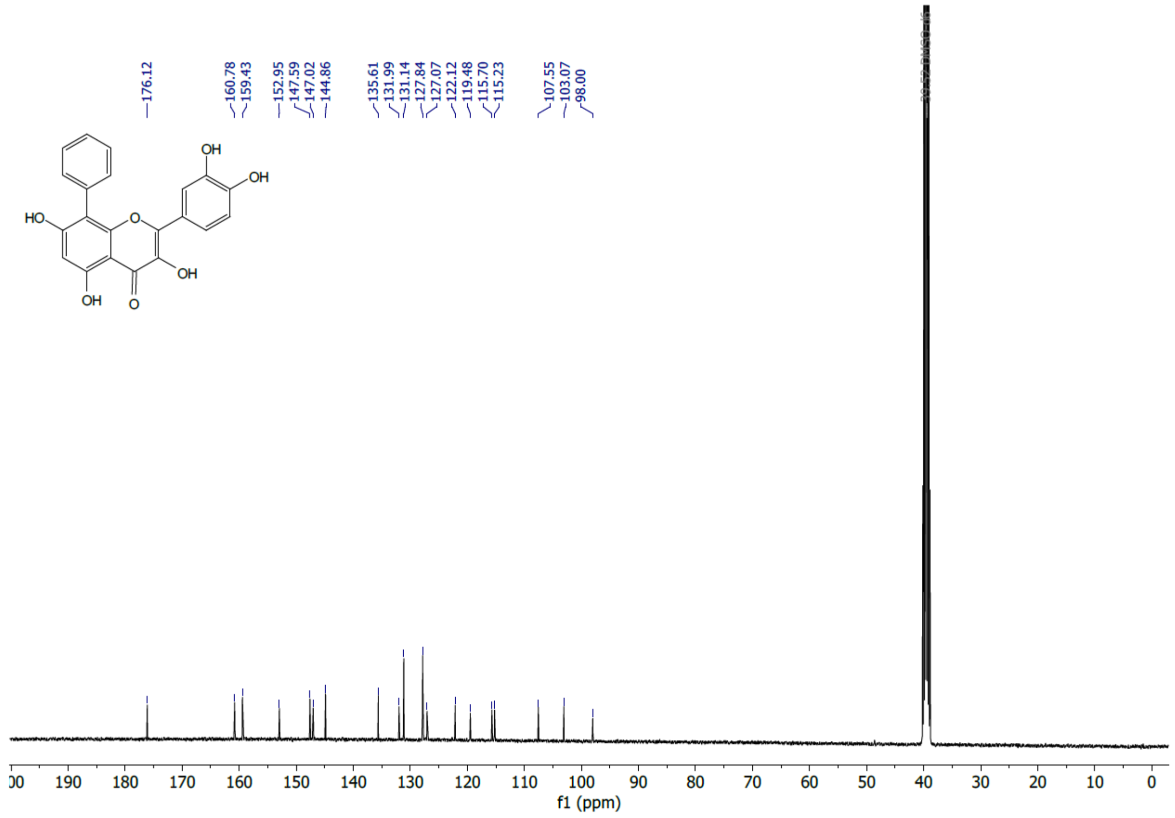

Figure S4: ^{13}C NMR (100 MHz, DMSO-*d*$_6$, 25 °C) spectrum of **20**.

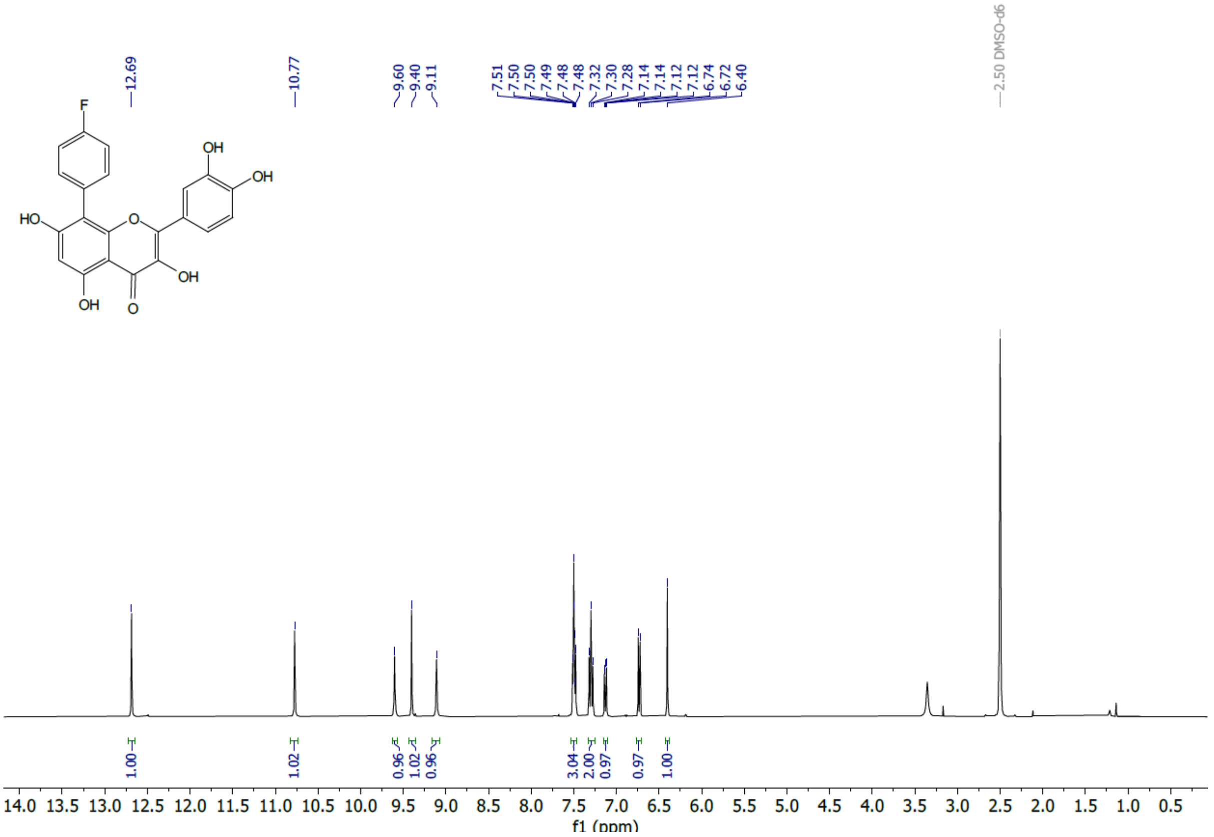

Figure S5: ^{1}H NMR (400 MHz, DMSO-*d*$_6$, 25 °C) spectrum of **21**.

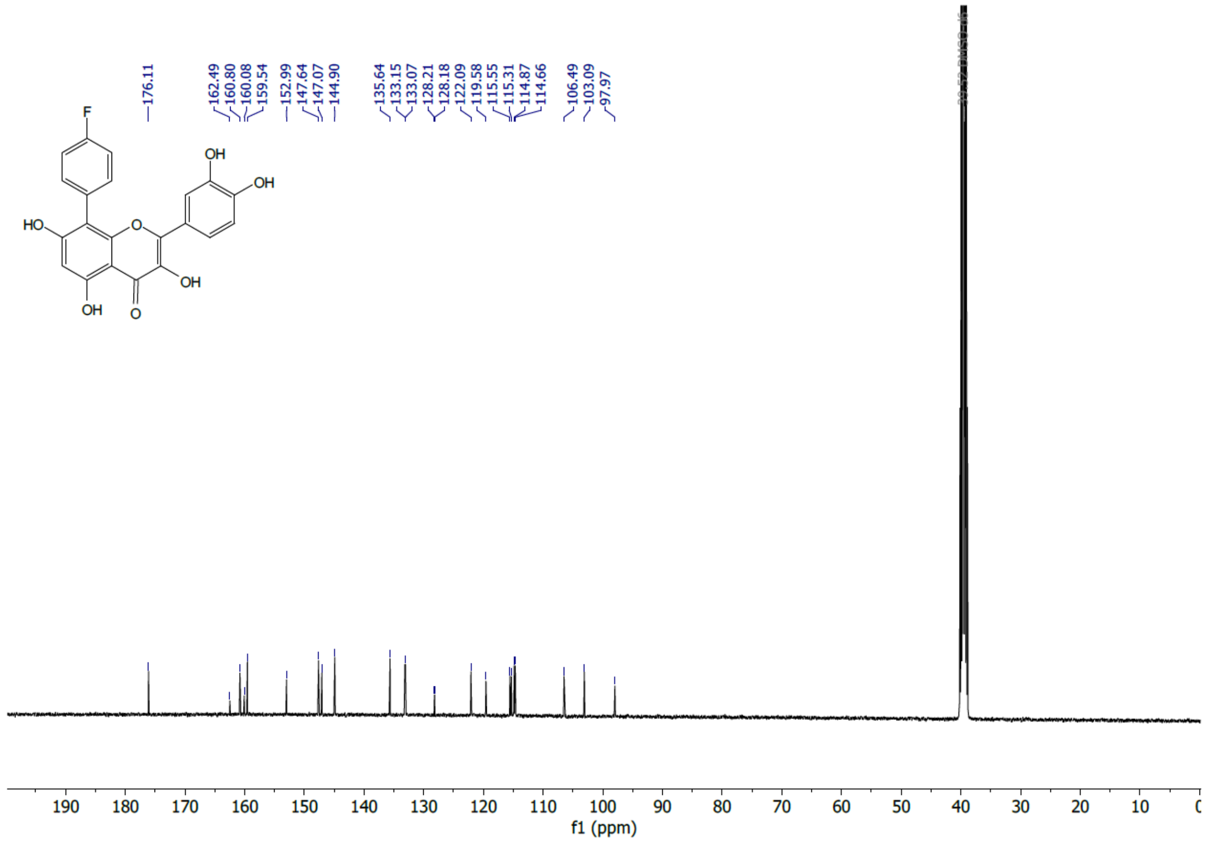

Figure S6: ^{13}C NMR (100 MHz, DMSO-*d*6, 25 °C) spectrum of **21**.

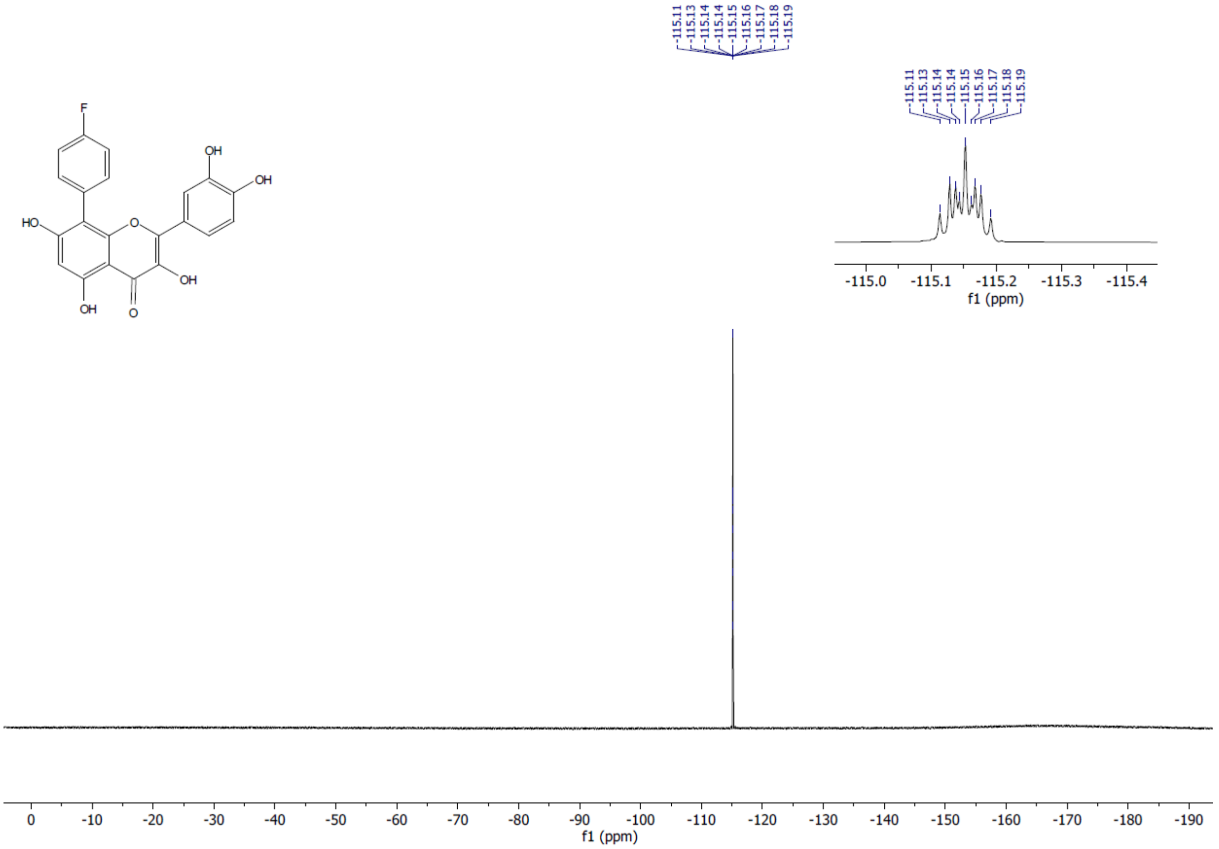

Figure S7: ^{19}F NMR (376 MHz, DMSO-*d*6, 25 °C) spectrum of **21**.

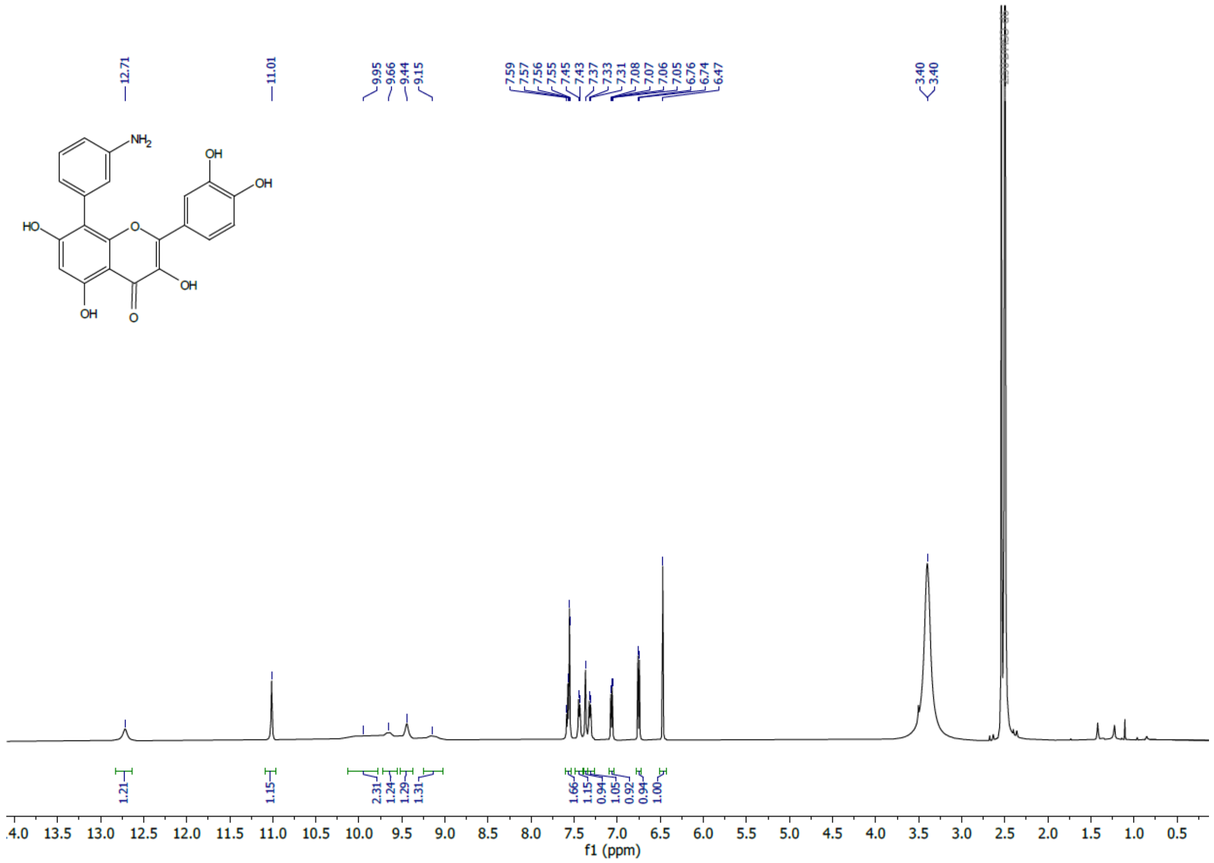

Figure S8: ^{1}H NMR (500 MHz, DMSO-d_6, 25 °C) spectrum of **22**.

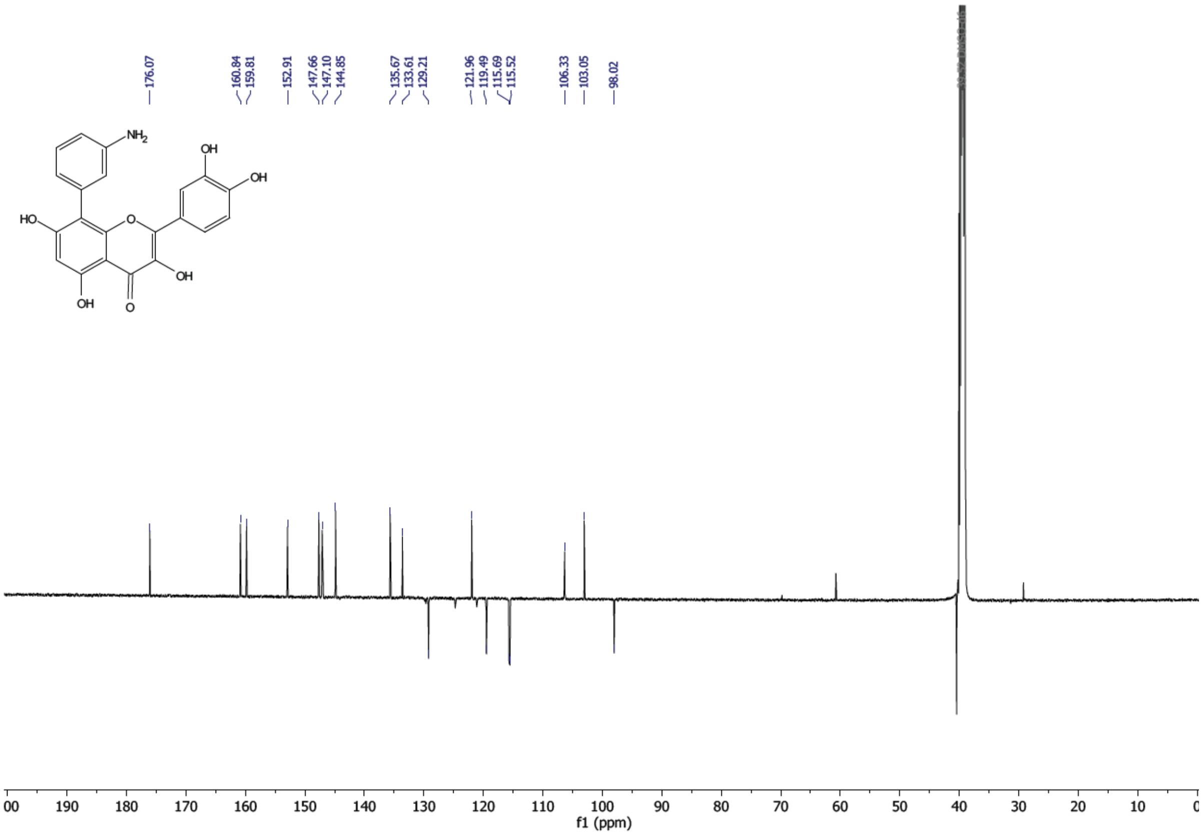

Figure S9: ^{13}C APT NMR (126 MHz, DMSO-d_6, 25 °C) spectrum of **22**.

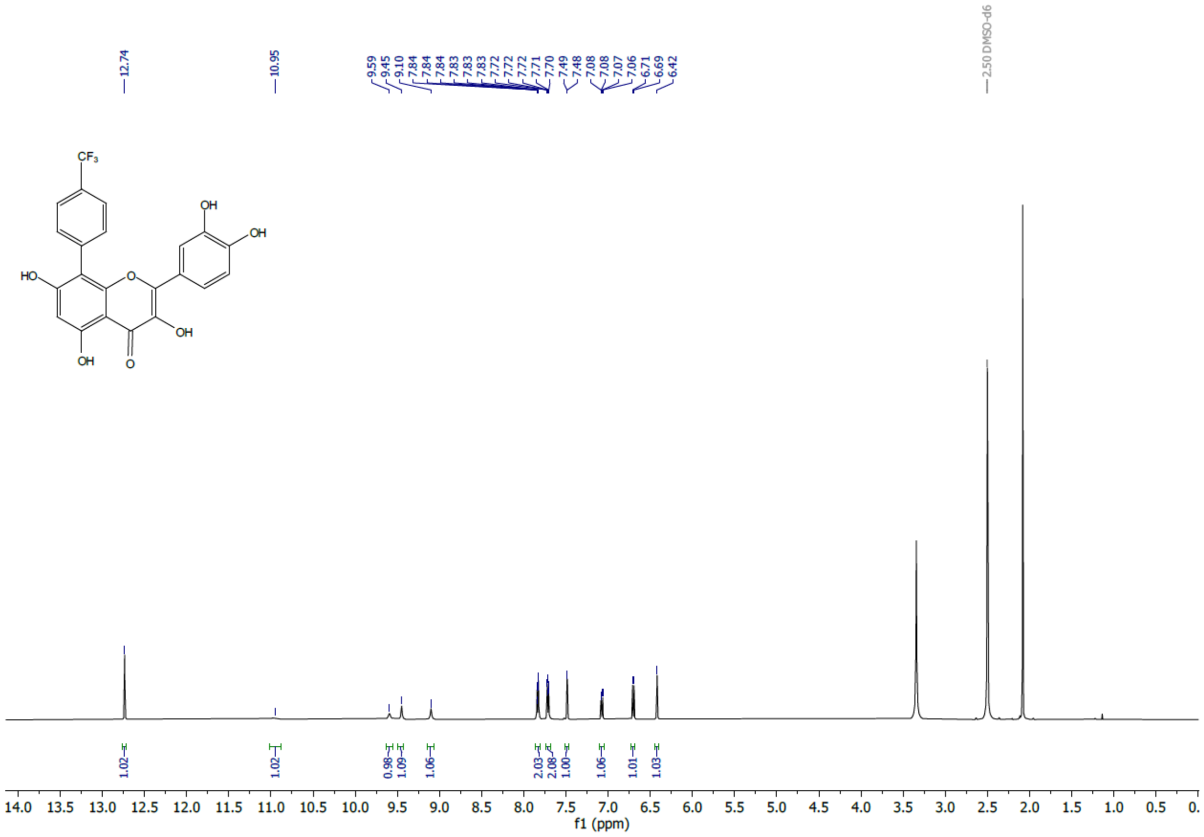

Figure S10: ^{1}H NMR (500 MHz, DMSO-d_6, 25 °C) spectrum of **23**.

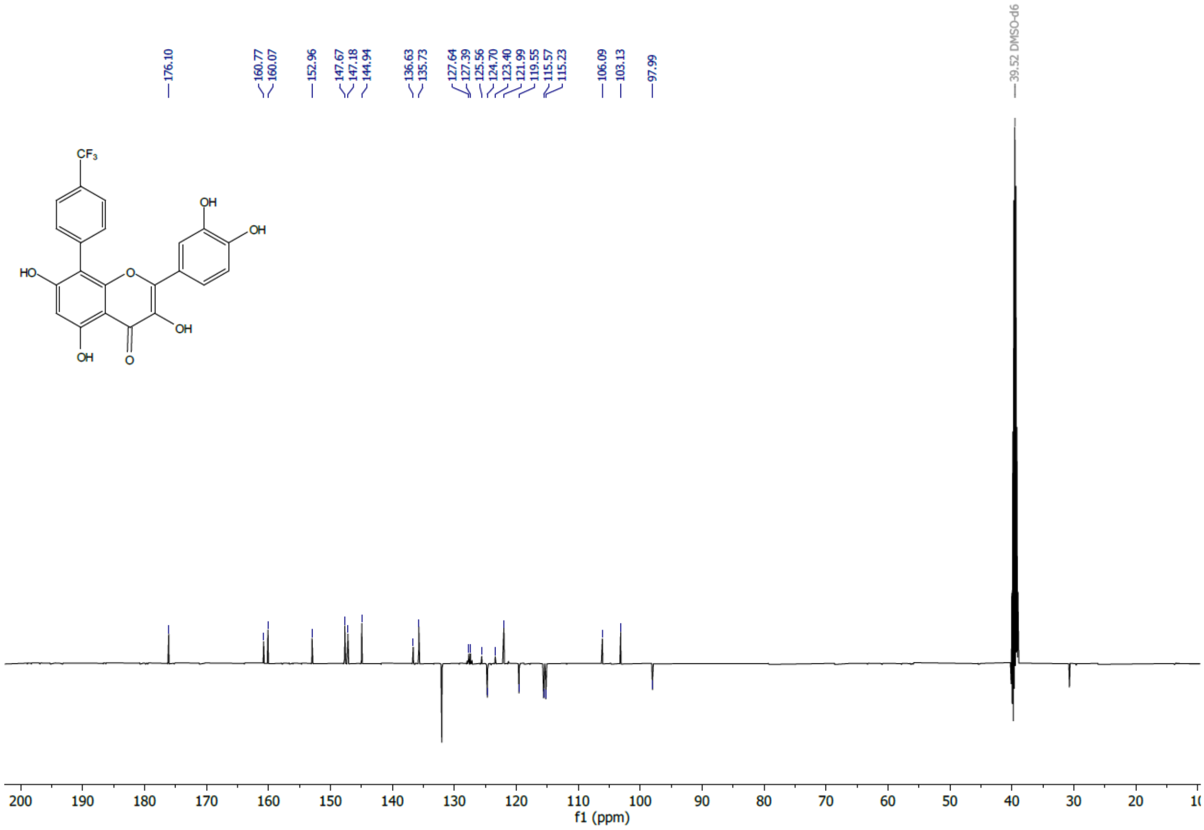

Figure S11: ^{13}C APT NMR (126 MHz, DMSO-d_6, 25 °C) spectrum of **23**.

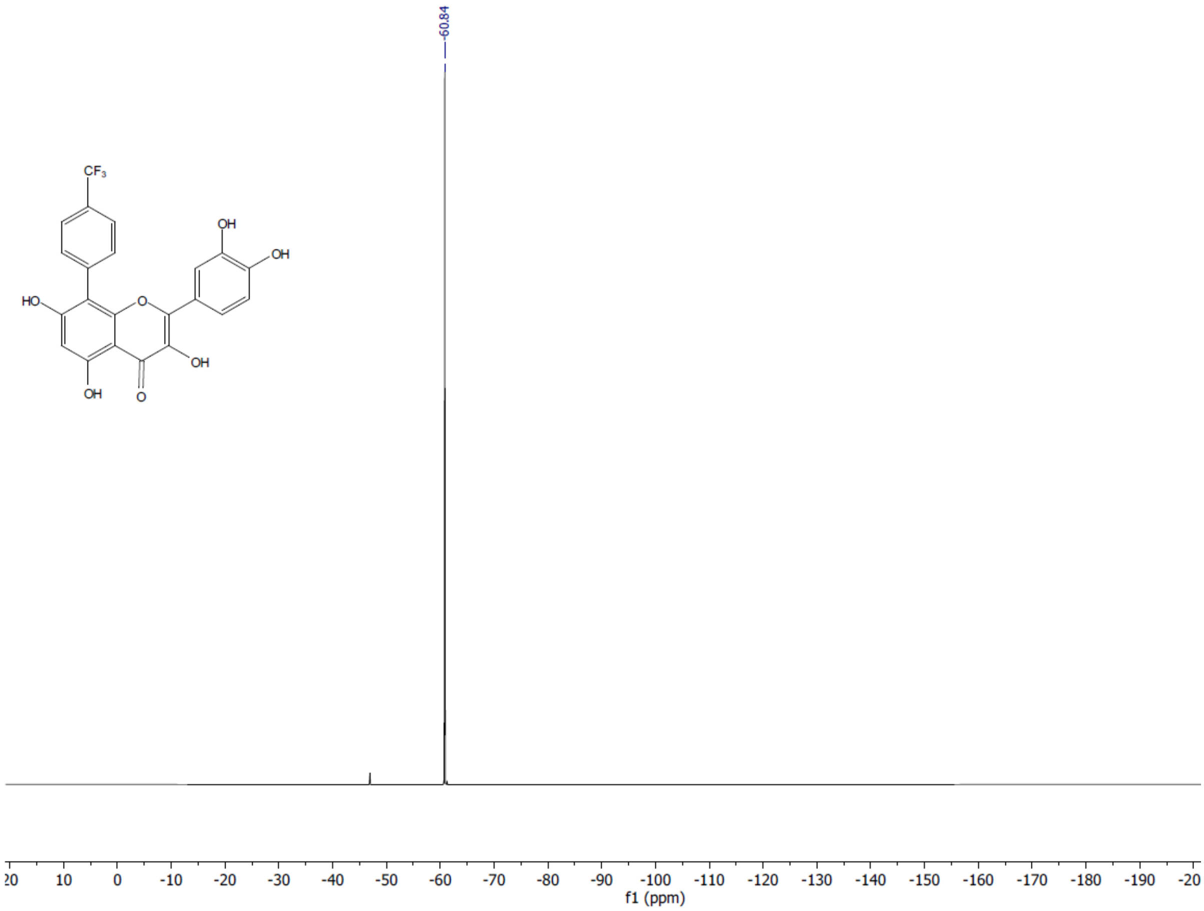

Figure S12: ^{19}F NMR (470 MHz, DMSO-*d*6, 25 °C) spectrum of **23**.

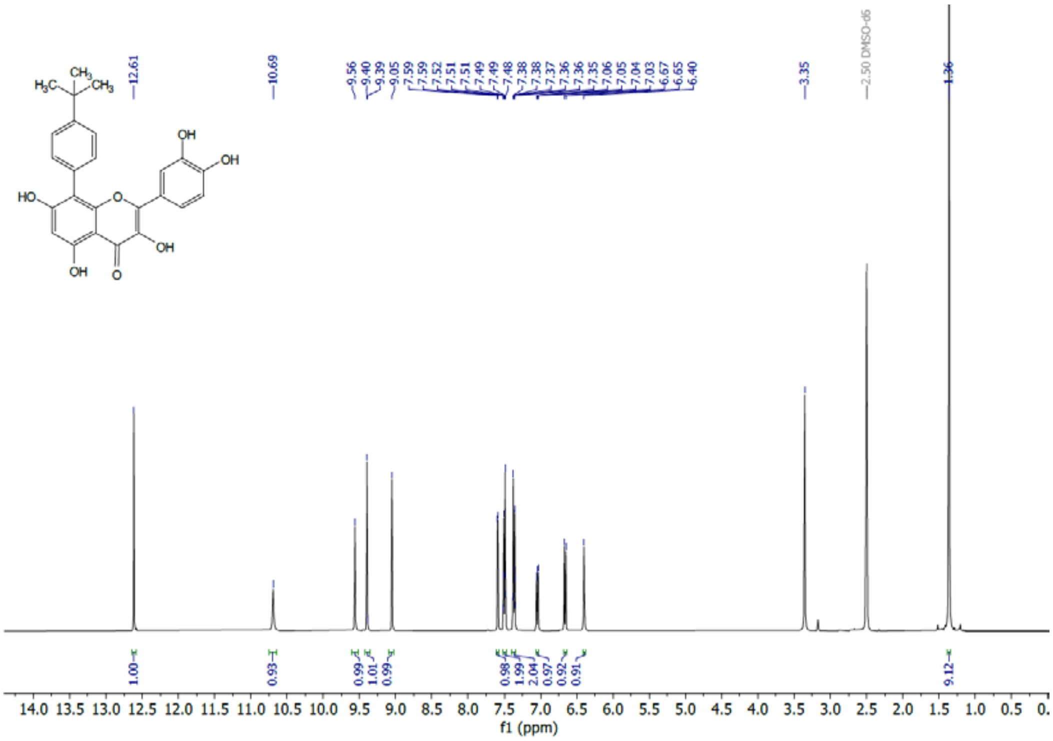

Figure S13: ^{1}H NMR (400 MHz, DMSO-*d*6, 25 °C) spectrum of **24**.

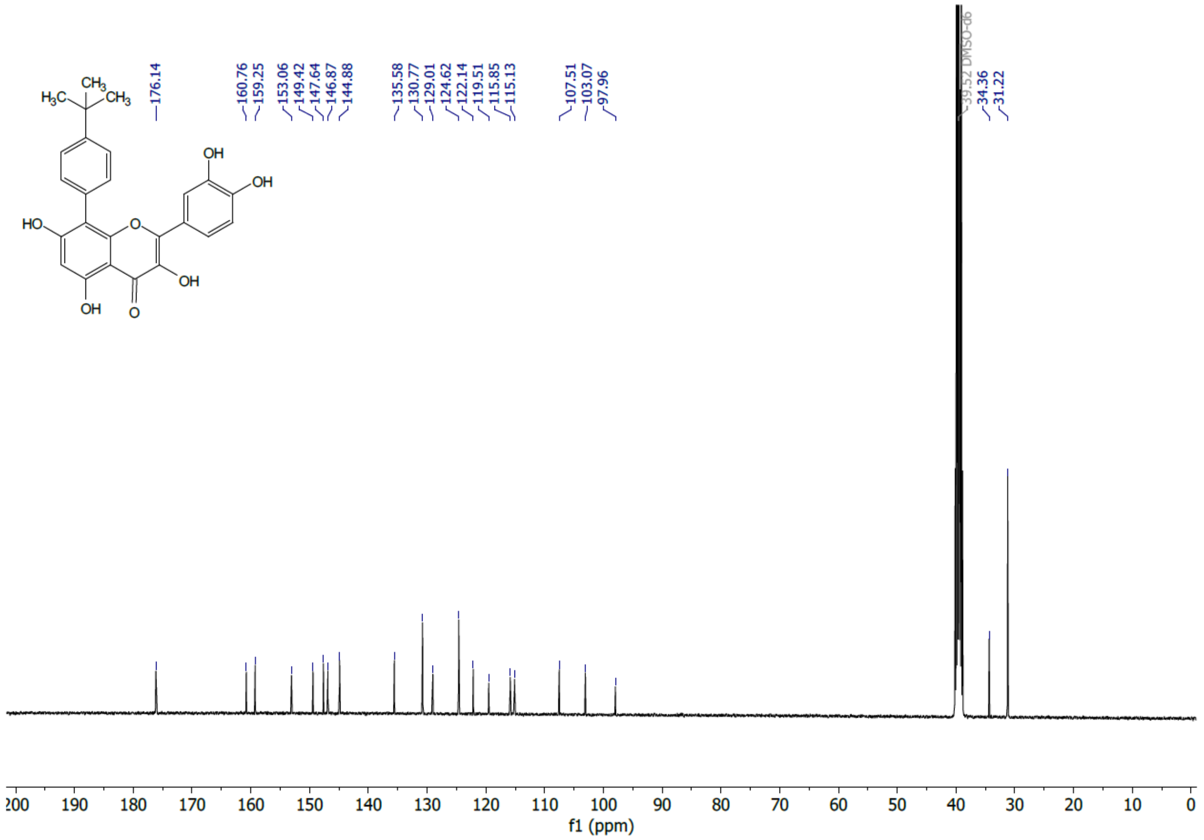

Figure S14: ^{13}C NMR (101 MHz, DMSO-*d*6, 25 °C) spectrum of **24**.

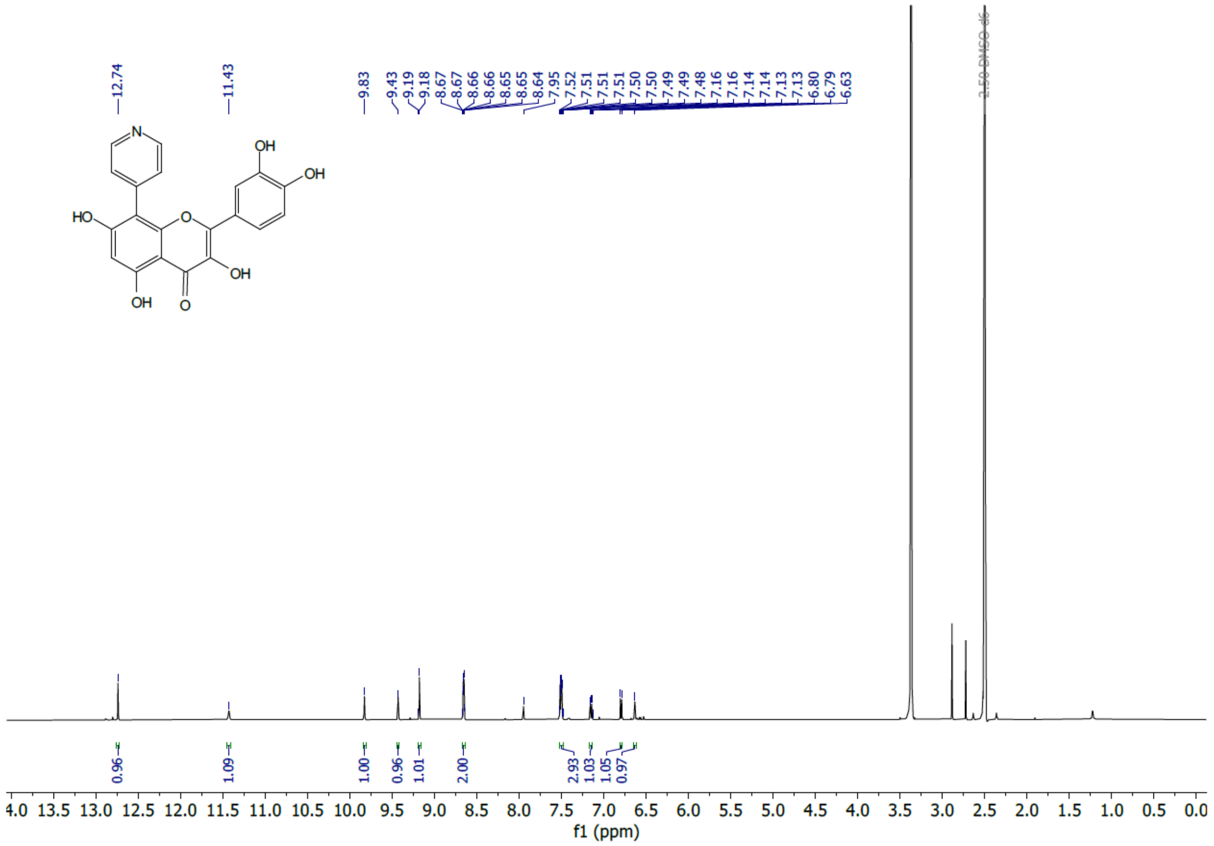

Figure S15: ^{1}H NMR (500 MHz, DMSO-*d*6, 25 °C) spectrum of **25**.

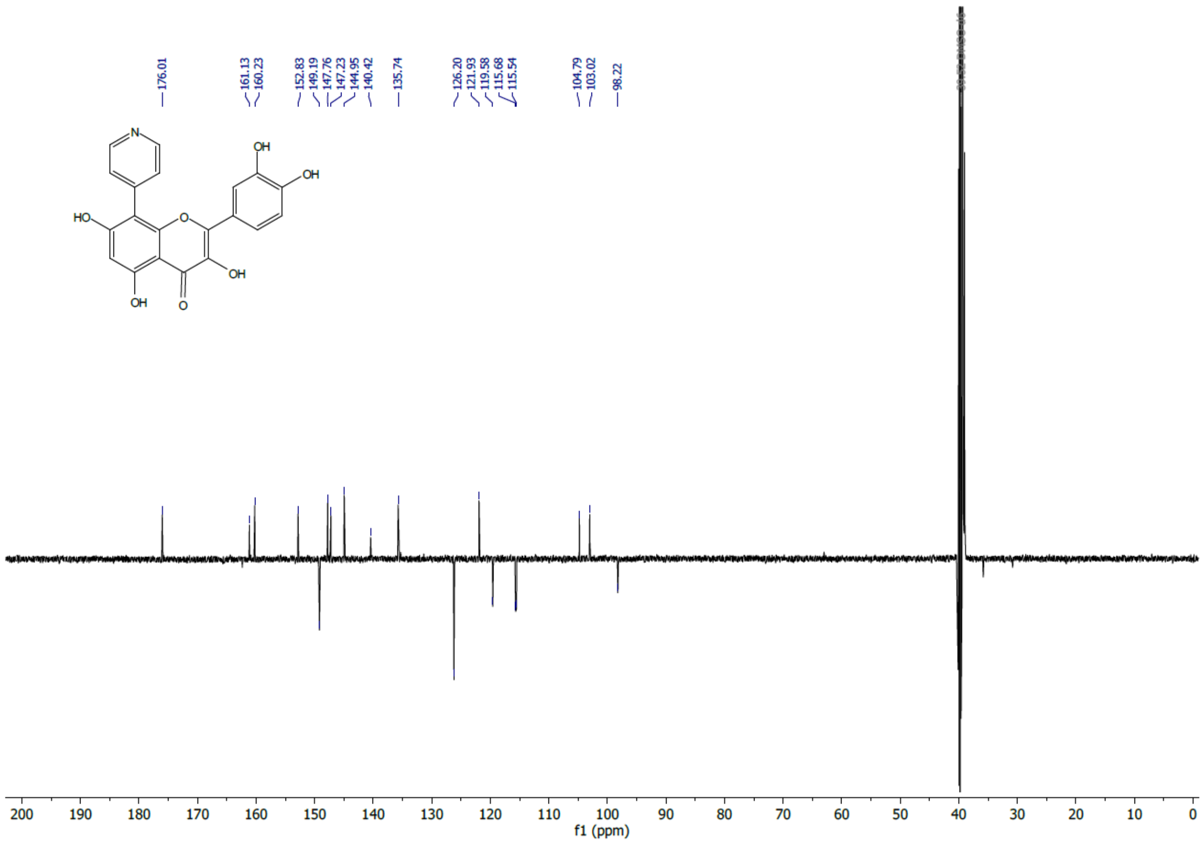

Figure S16: ^{13}C APT NMR (126 MHz, DMSO-d_6, 25 °C) spectrum of **25**.

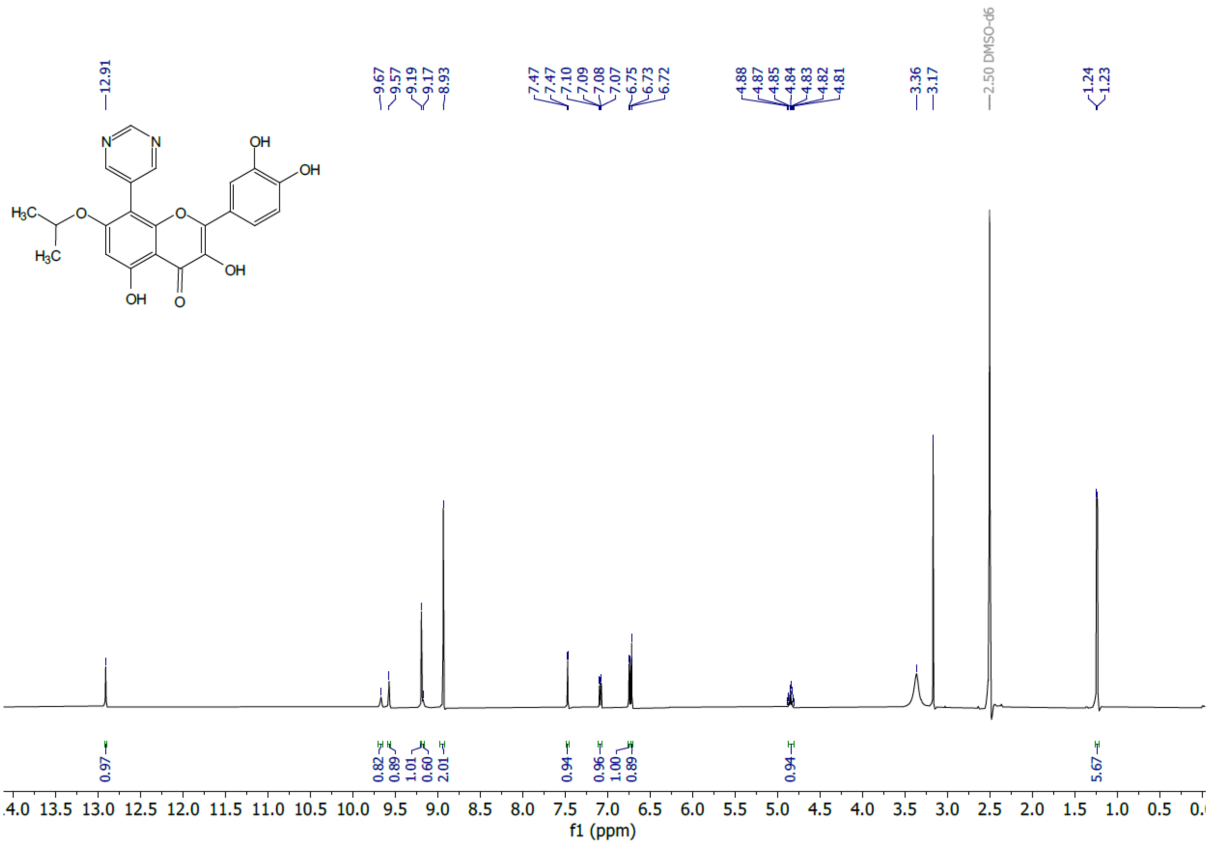

Figure S17: ^{1}H NMR (500 MHz, DMSO-d_6, 25 °C) spectrum of **26**.

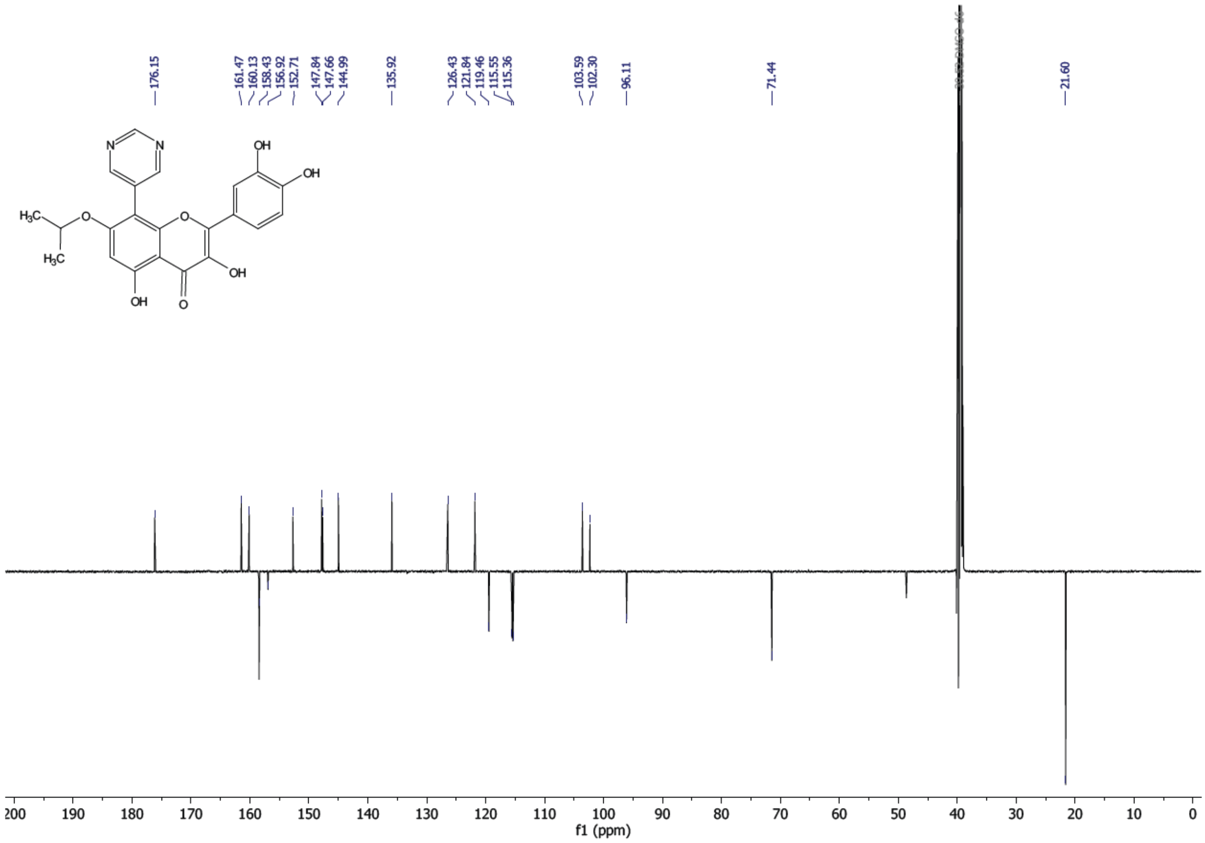

Figure S18: ^{13}C APT NMR (126 MHz, DMSO-*d*$_6$, 25 °C) spectrum of **26**.

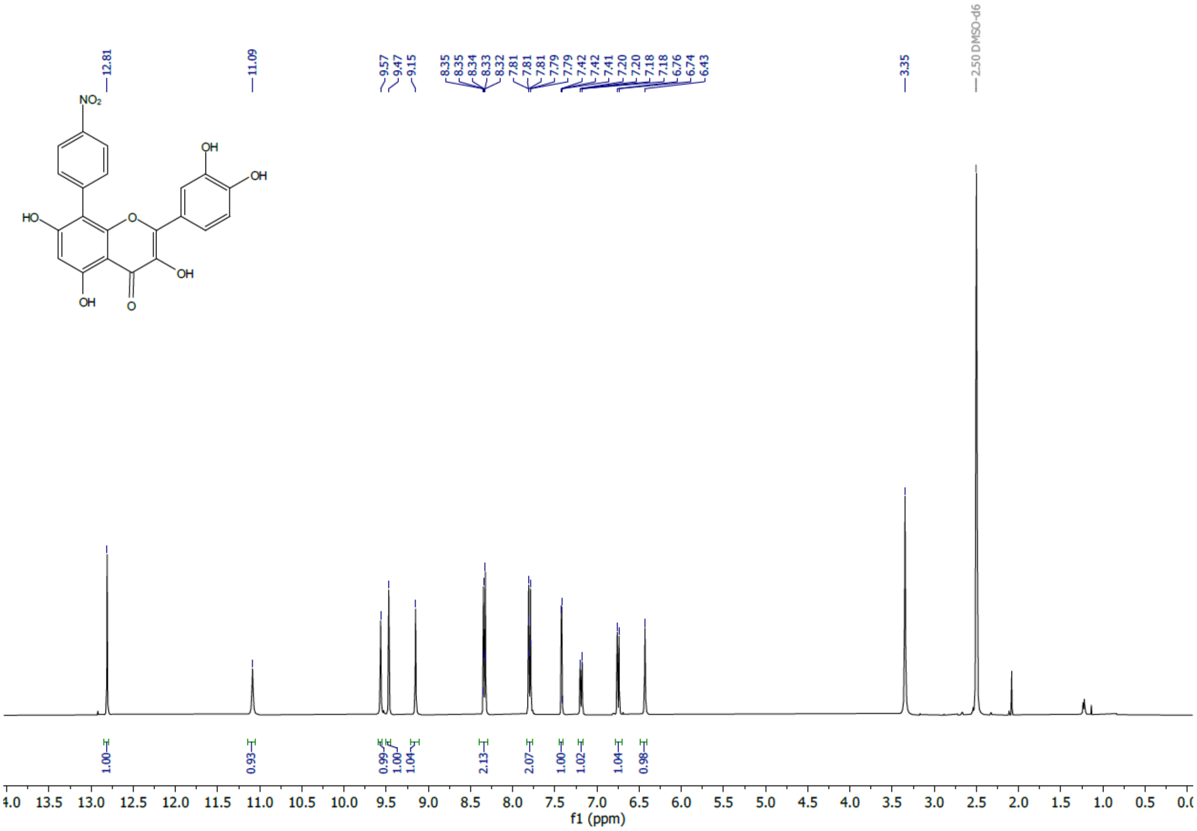

Figure S19: ^{1}H NMR (500 MHz, DMSO-*d*$_6$, 25 °C) spectrum of **27**.

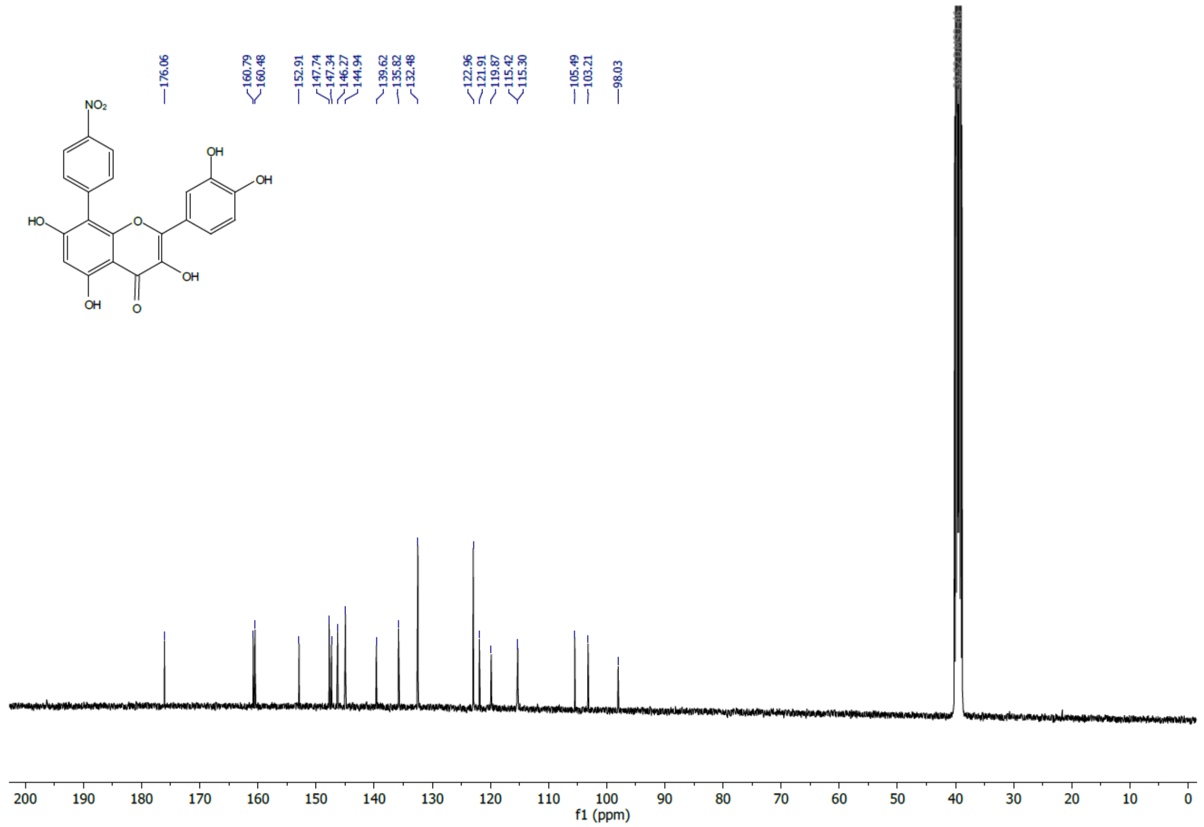

Figure S20: ^{13}C NMR (101 MHz, DMSO-d_6, 25 °C) spectrum of **27**.

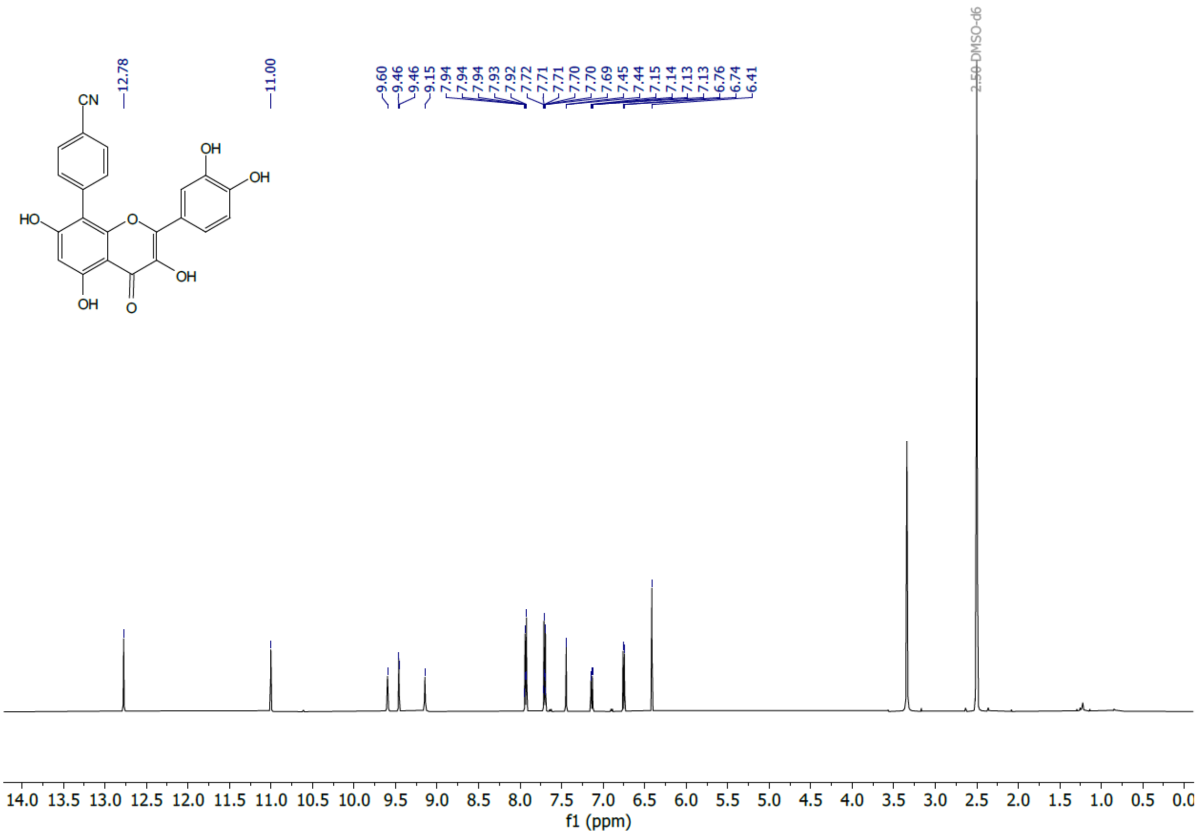

Figure S21: ^{1}H NMR (500 MHz, DMSO-d_6, 25 °C) spectrum of **28**.

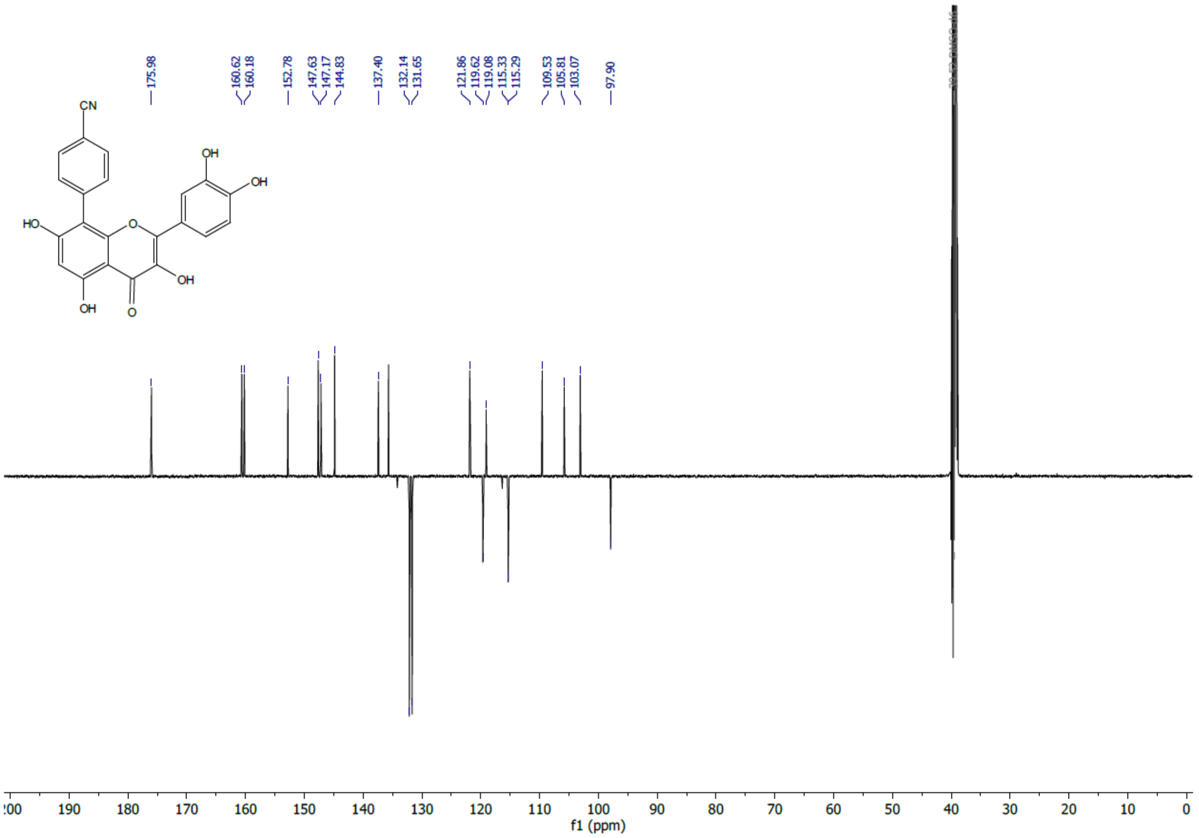

Figure S22: ^{13}C APT NMR (126 MHz, DMSO-d_6, 25 °C) spectrum of **28**.

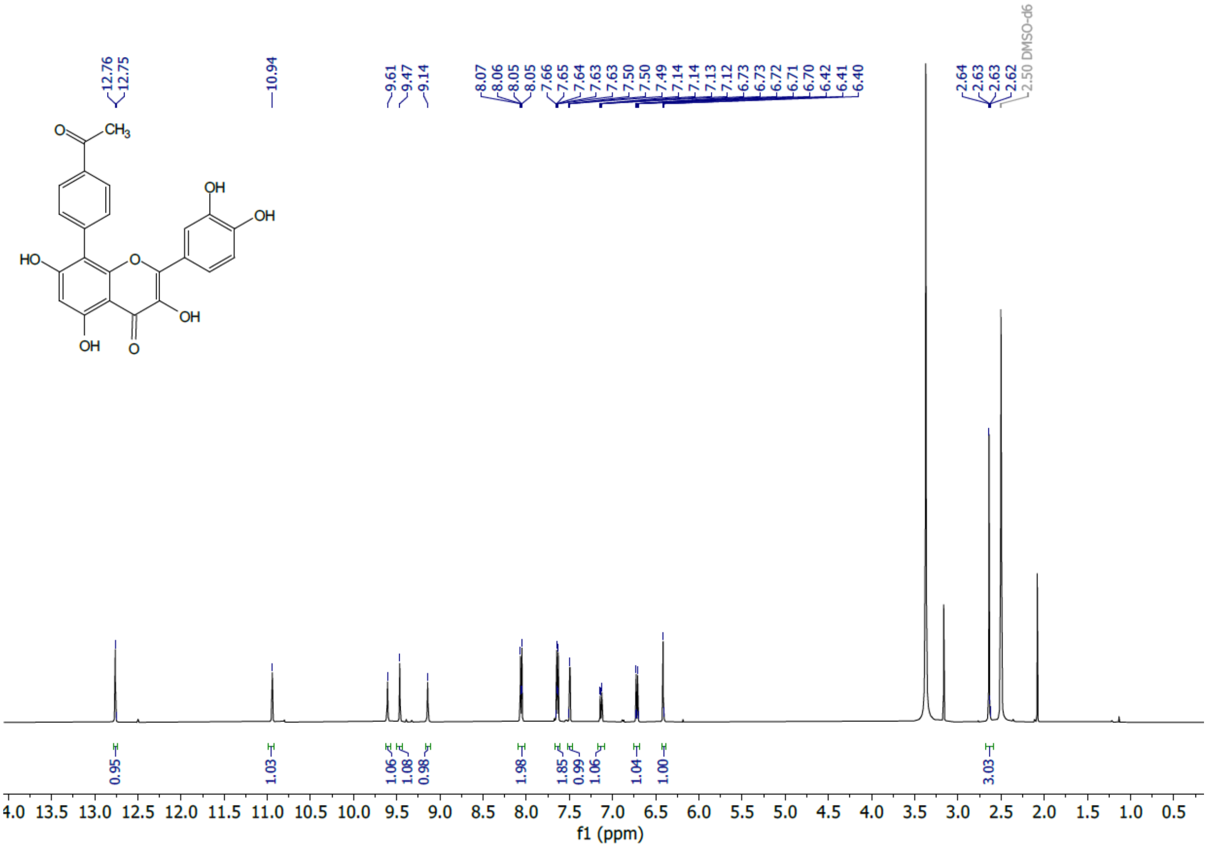

Figure S23: ^{1}H NMR (500 MHz, DMSO-d_6, 25 °C) spectrum of **29**.

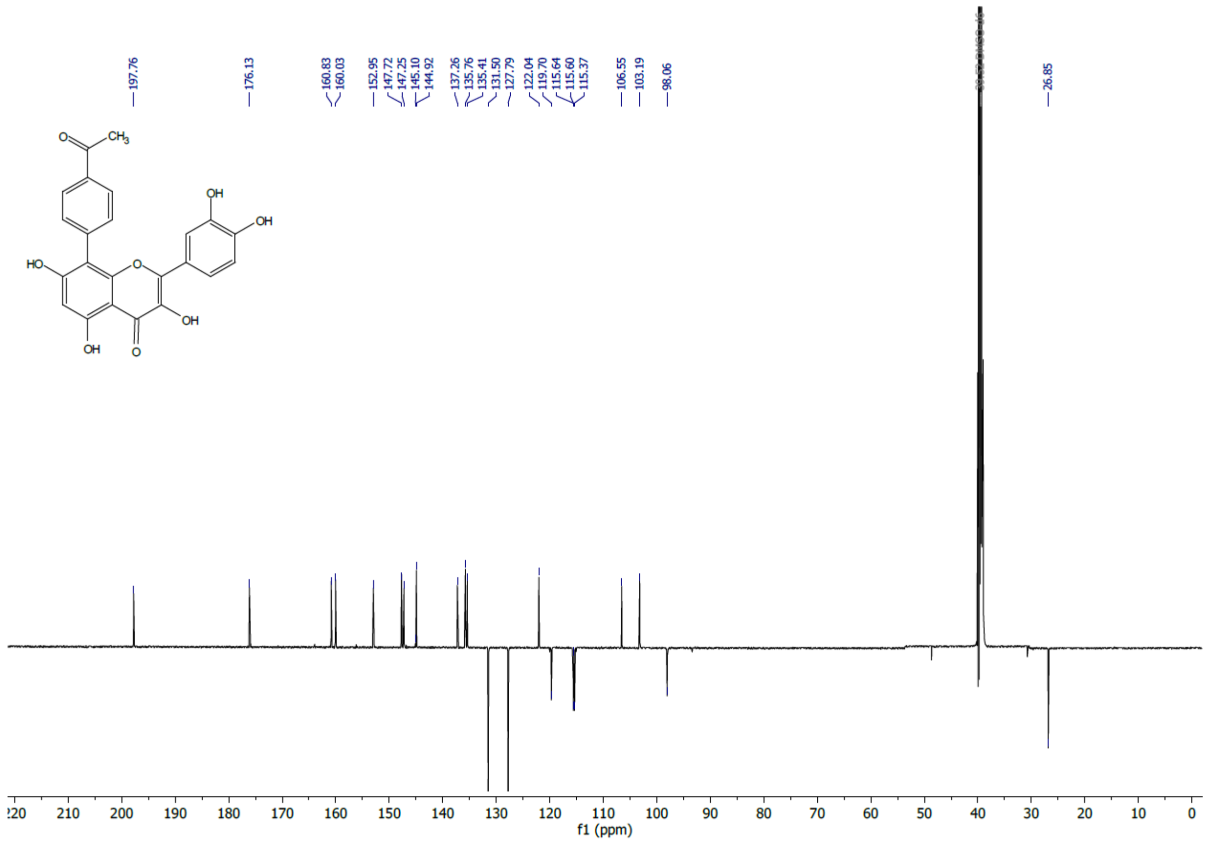

Figure S24: ^{13}C APT NMR (126 MHz, DMSO-*d*6, 25 °C) spectrum of **29**.

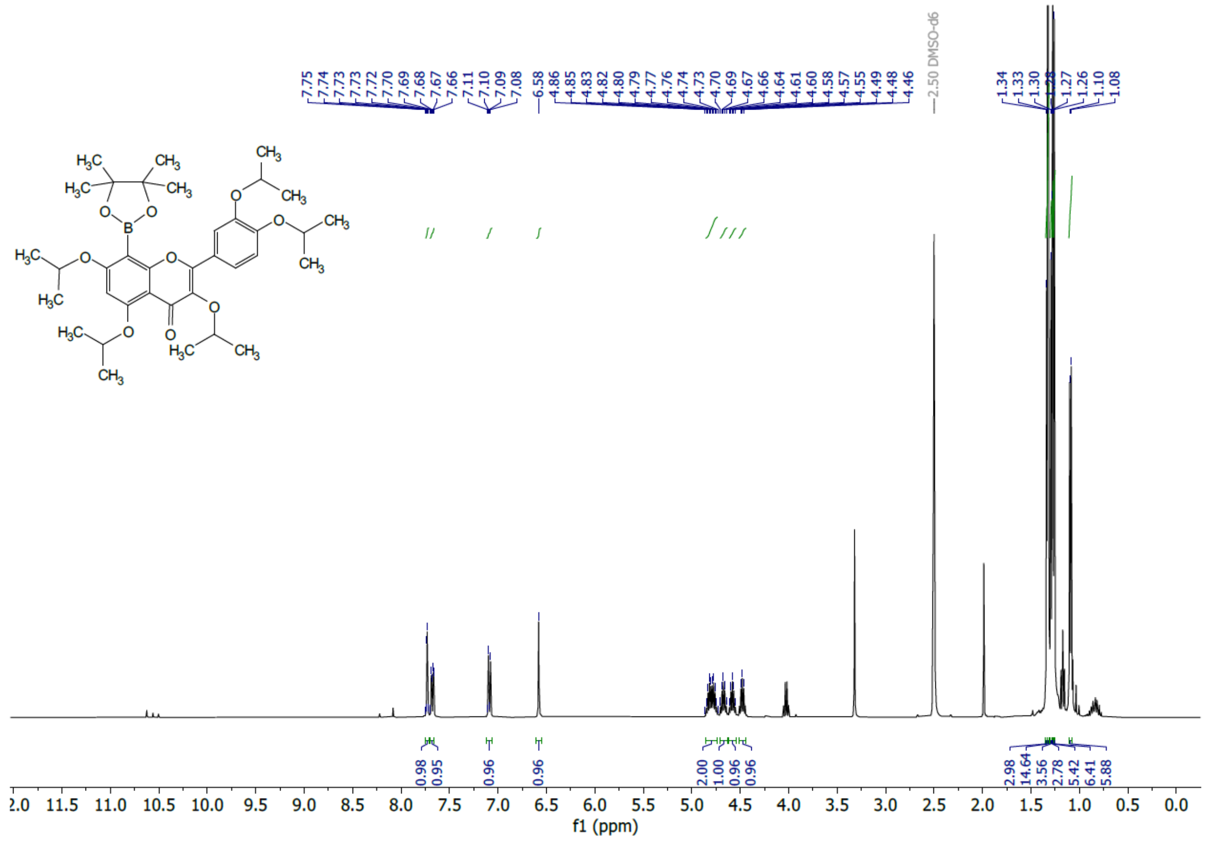

Figure S25: ^{1}H NMR (400 MHz, DMSO-*d*6, 25 °C) spectrum of **7**.

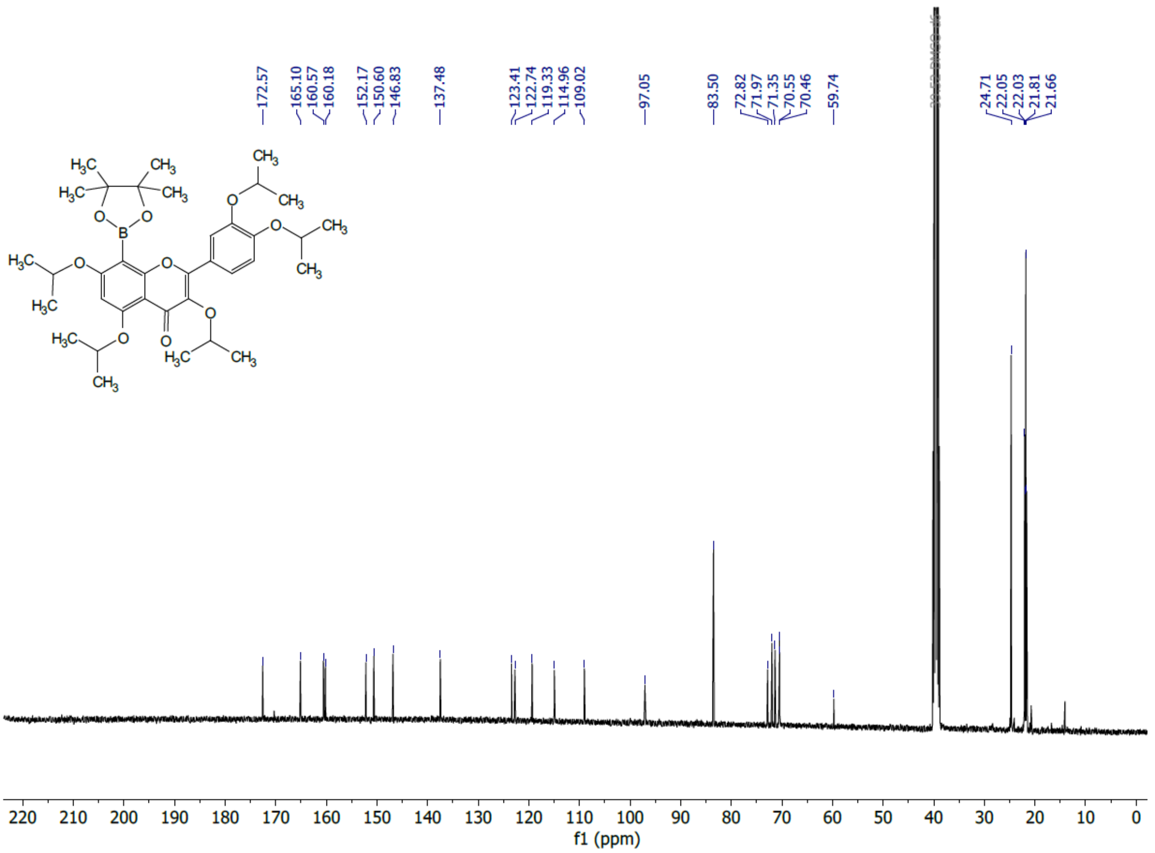

Figure S26: ^{13}C NMR (101 MHz, DMSO-*d*6, 25 °C) spectrum of **7**.

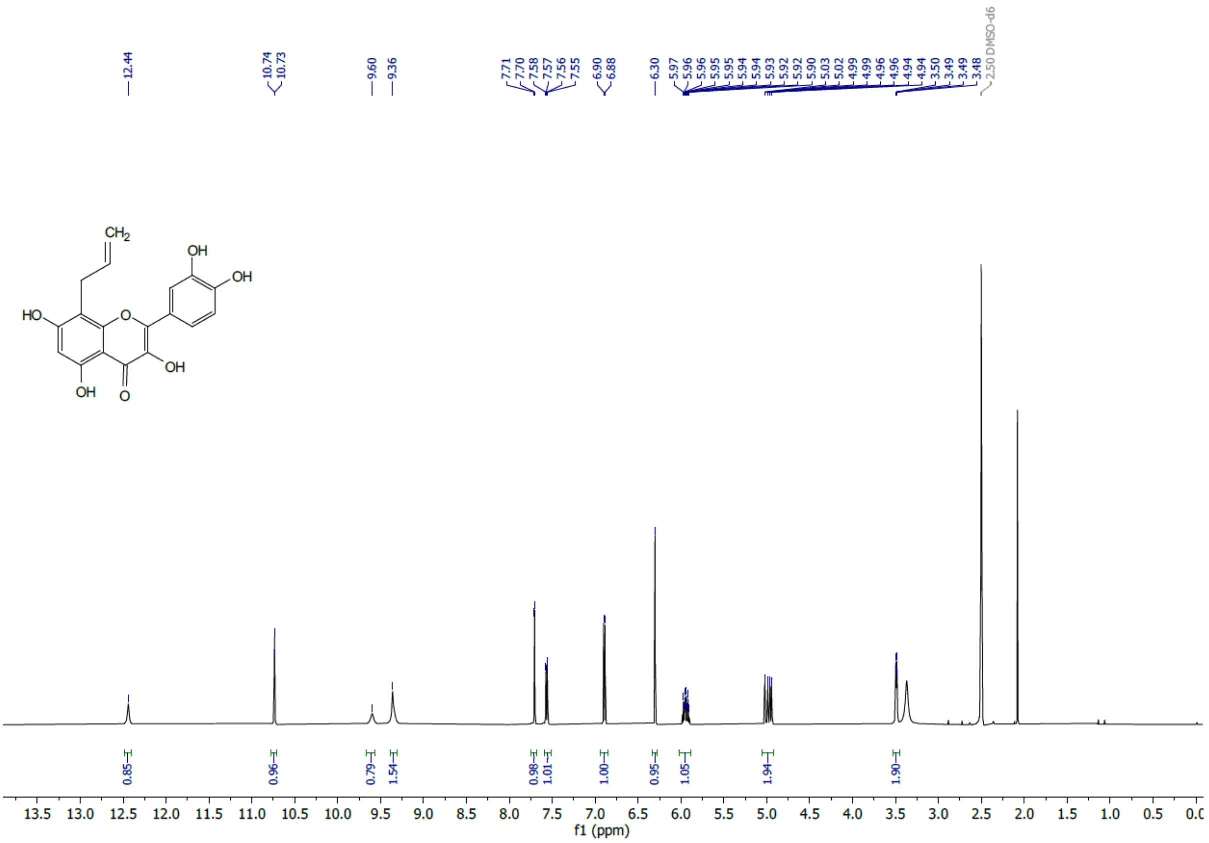

Figure S27: ^{1}H NMR (500 MHz, DMSO-*d*6, 25 °C) spectrum of **9**.

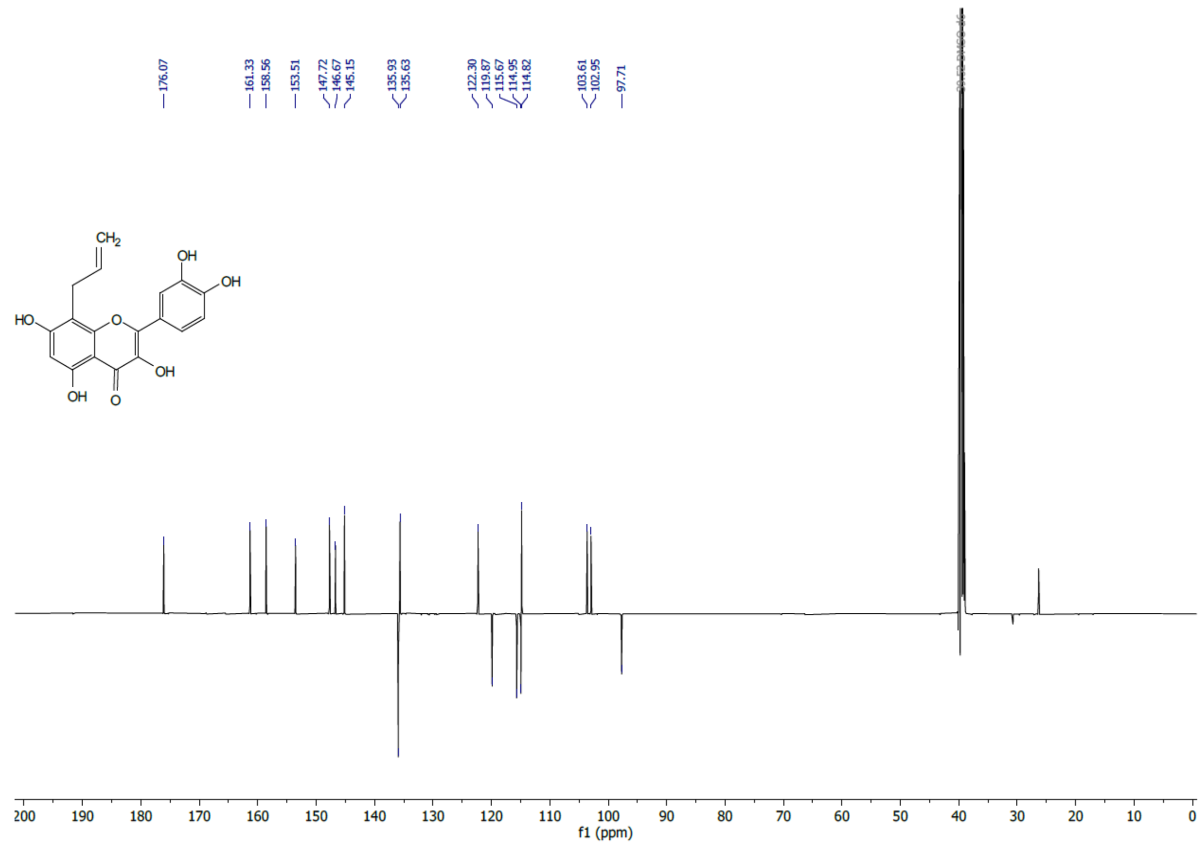

Figure S28: ^{13}C APT NMR (126 MHz, DMSO-d_6, 25 °C) spectrum of **9**.

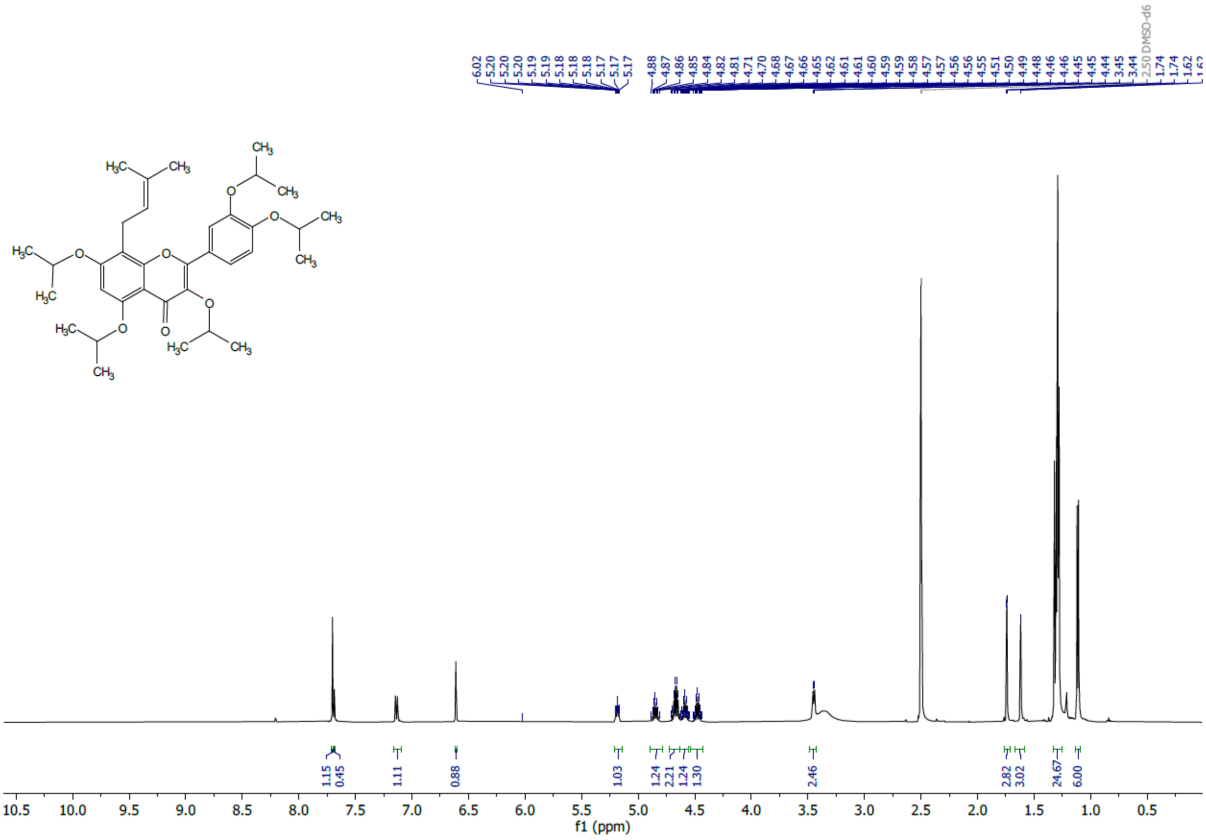

Figure S29: ^{1}H NMR (500 MHz, DMSO-d_6, 25 °C) spectrum of **10**.

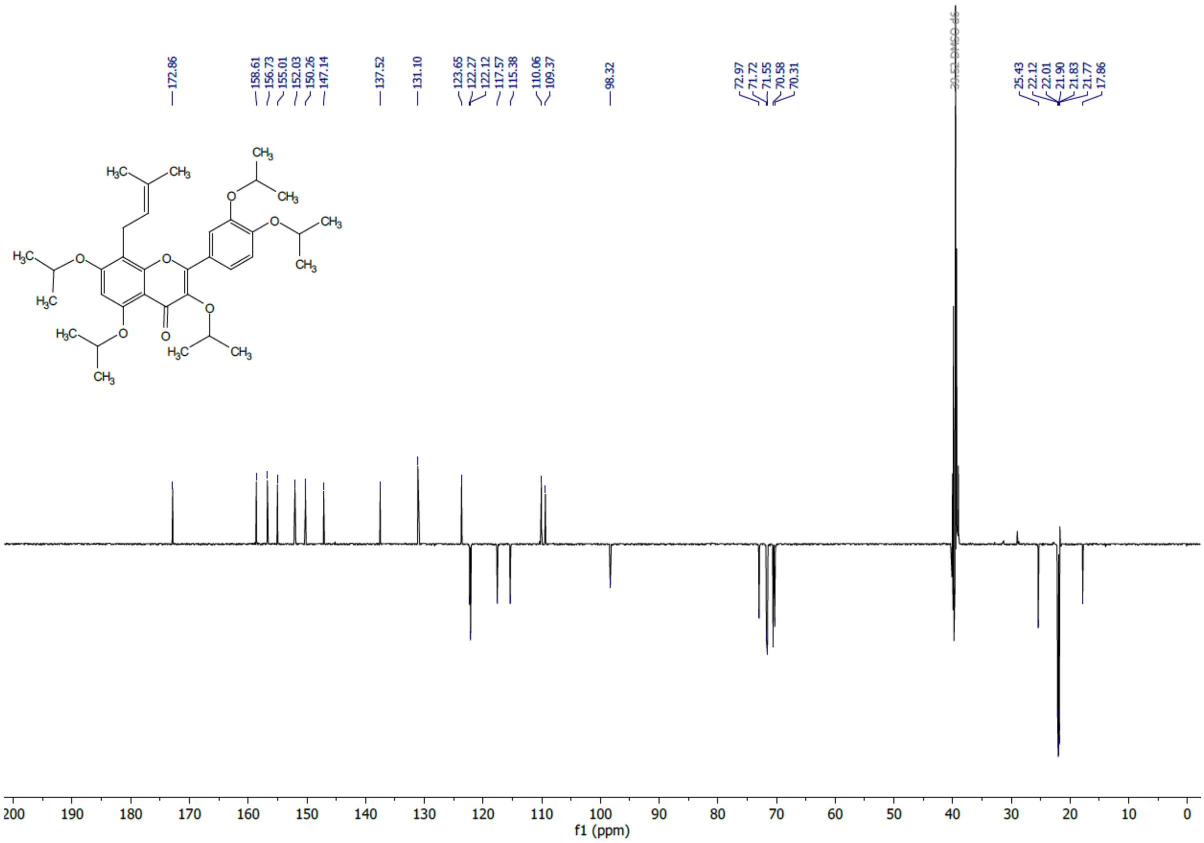

Figure S30: ^{13}C APT NMR (126 MHz, DMSO-d_6, 25 °C) spectrum of **10**.

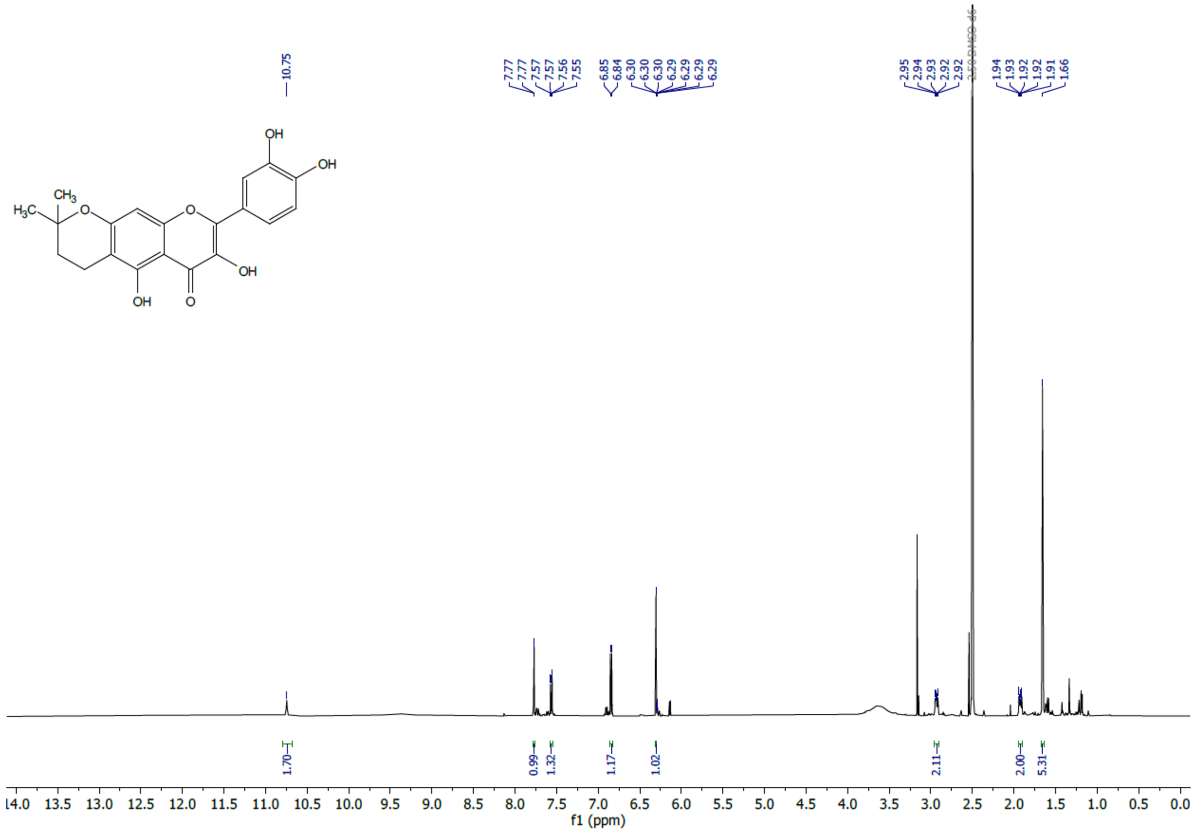

Figure S31: ^{1}H NMR (500 MHz, DMSO-d_6, 25 °C) spectrum of **11b**.

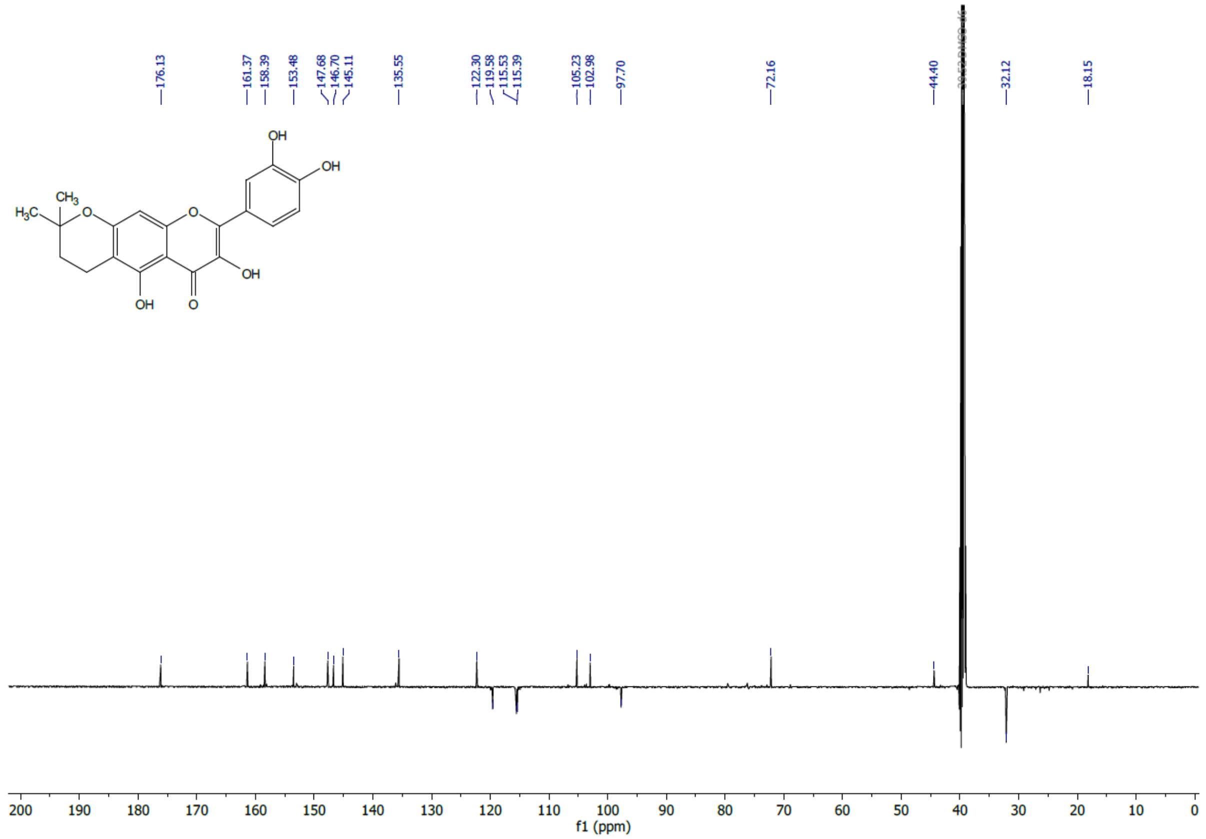

Figure S32: ^{13}C NMR (126 MHz, DMSO-*d*6, 25 °C) spectrum of **11b**.

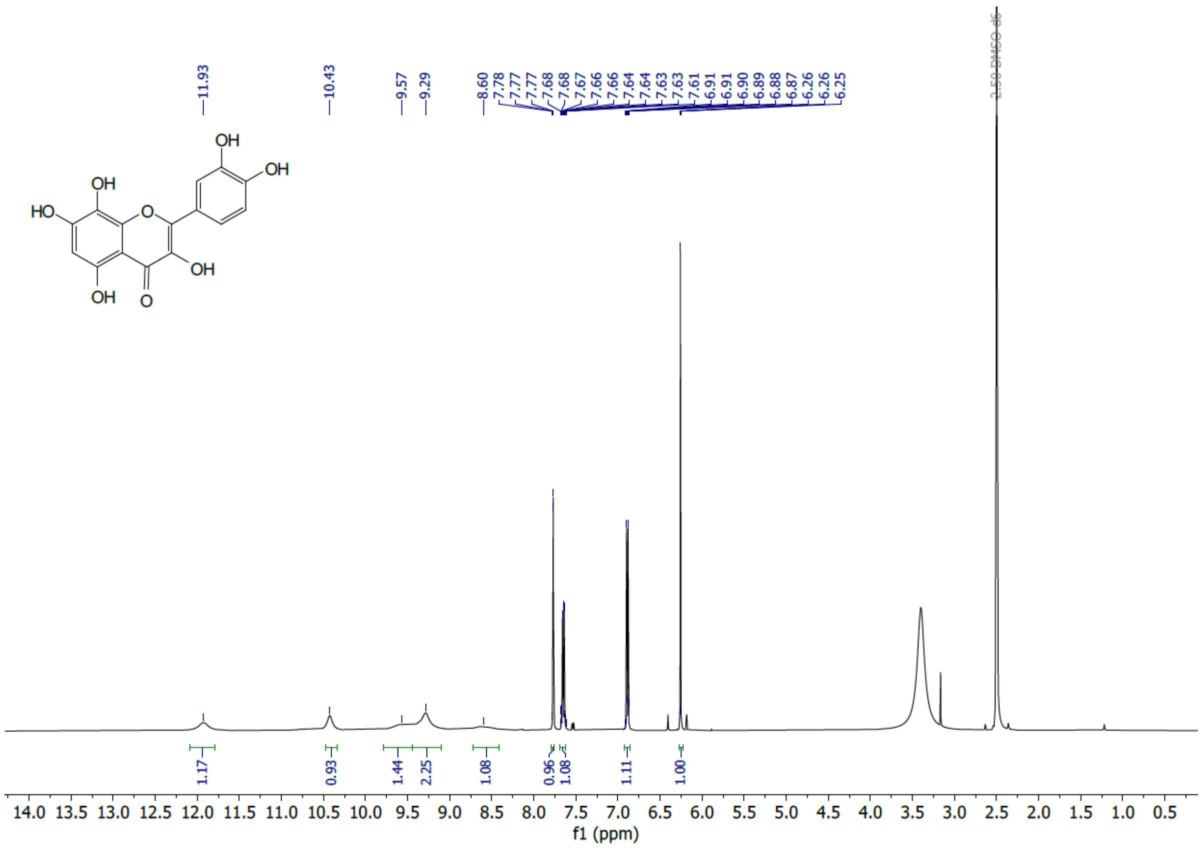

Figure S33: ^{1}H NMR (500 MHz, DMSO-*d*6, 25 °C) spectrum of **30**.

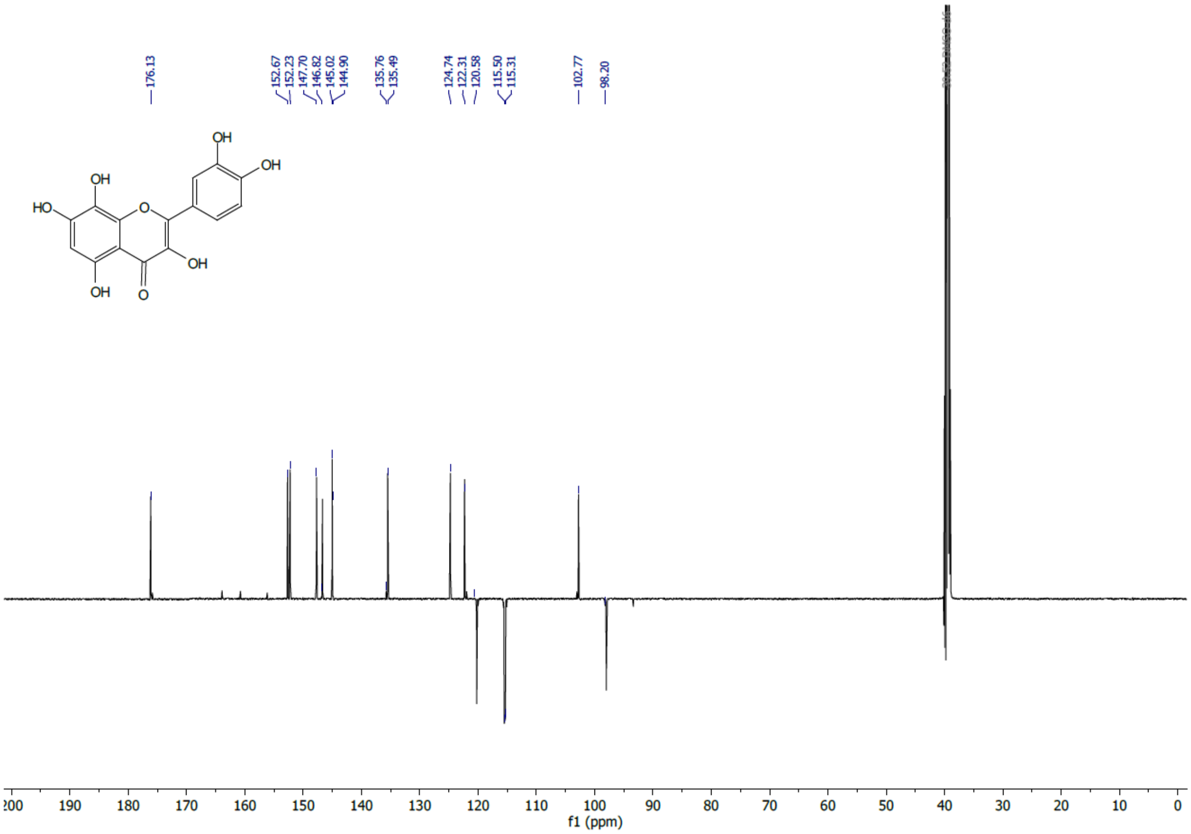

Figure S34: ^{13}C APT NMR (126 MHz, DMSO-*d*6, 25 °C) spectrum of **30**.

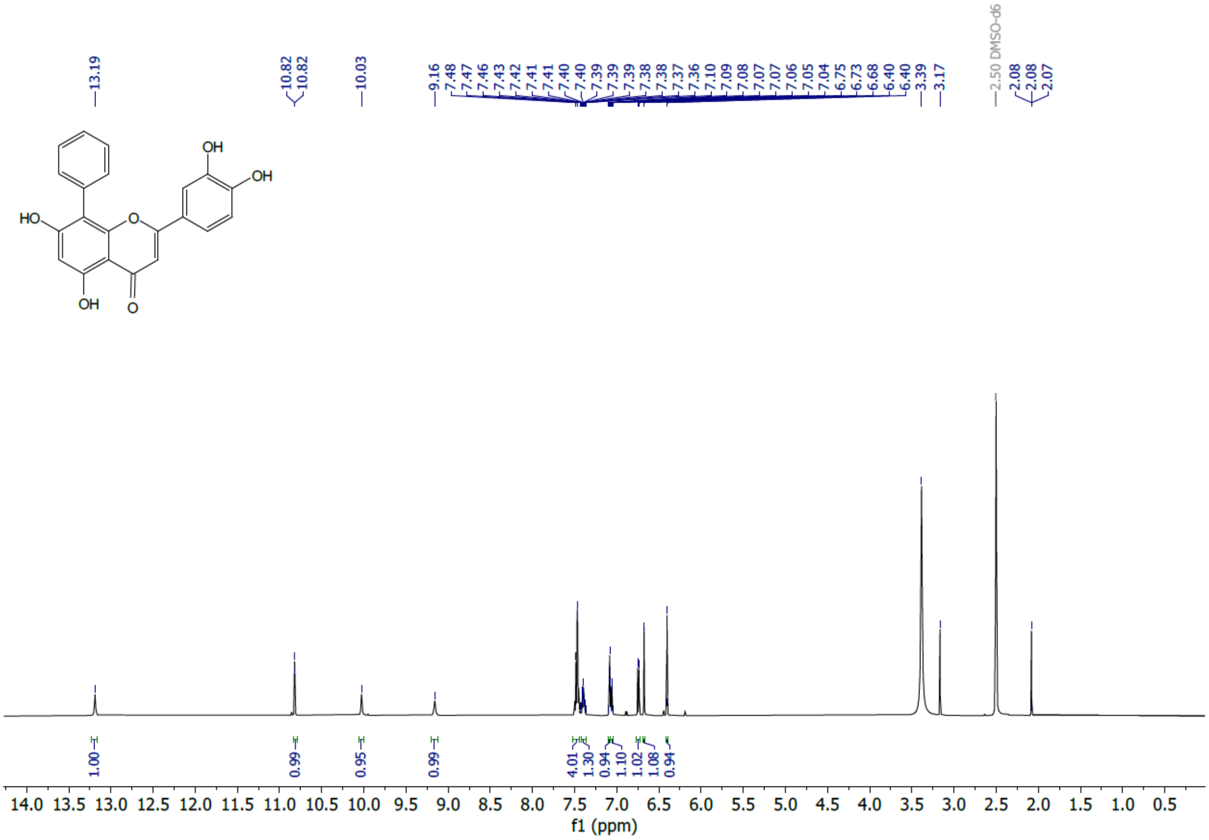

Figure S35: ^{1}H NMR (500 MHz, DMSO-*d*6, 25 °C) spectrum of **31**.

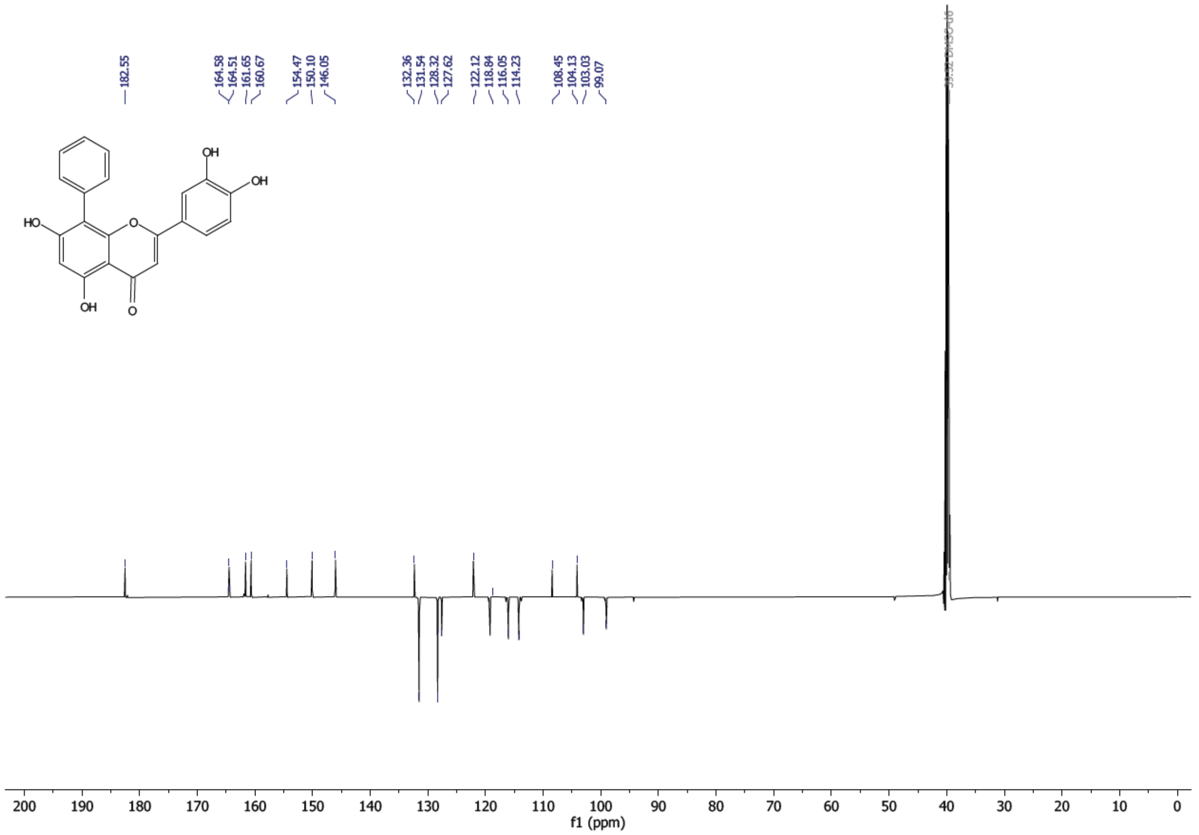

Figure S36: ^{13}C APT NMR (126 MHz, DMSO-d_6, 25 °C) spectrum of **31**.

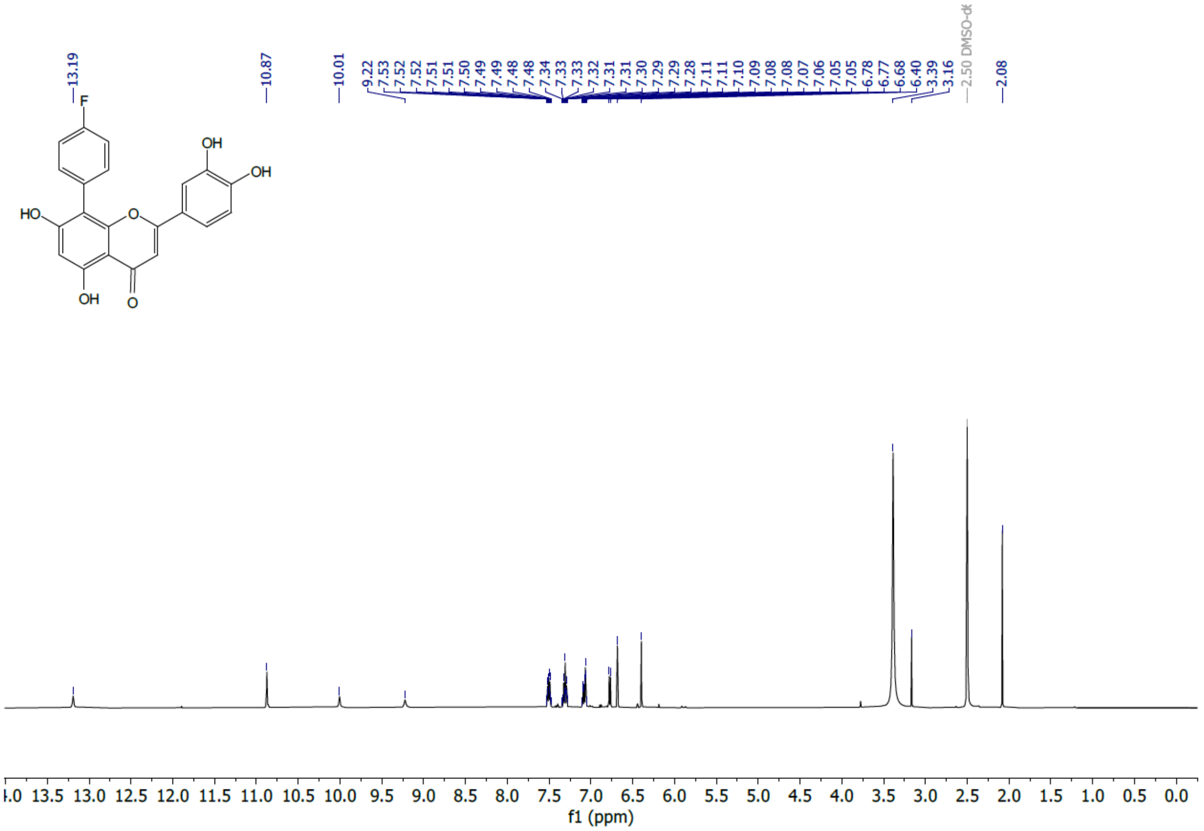

Figure S37: ^{1}H NMR (500 MHz, DMSO-d_6, 25 °C) spectrum of **32**.

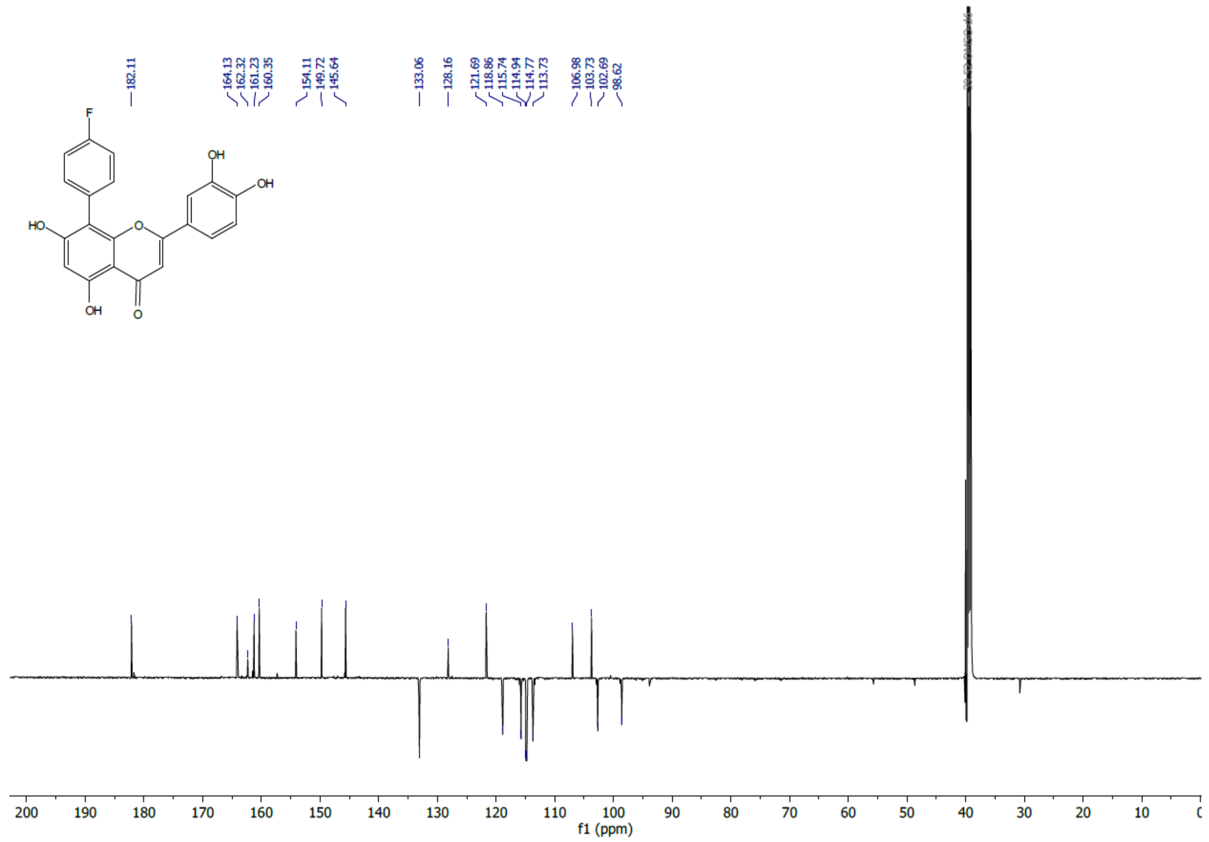

Figure S38: ^{13}C APT NMR (126 MHz, DMSO-*d*6, 25 °C) spectrum of **32**.

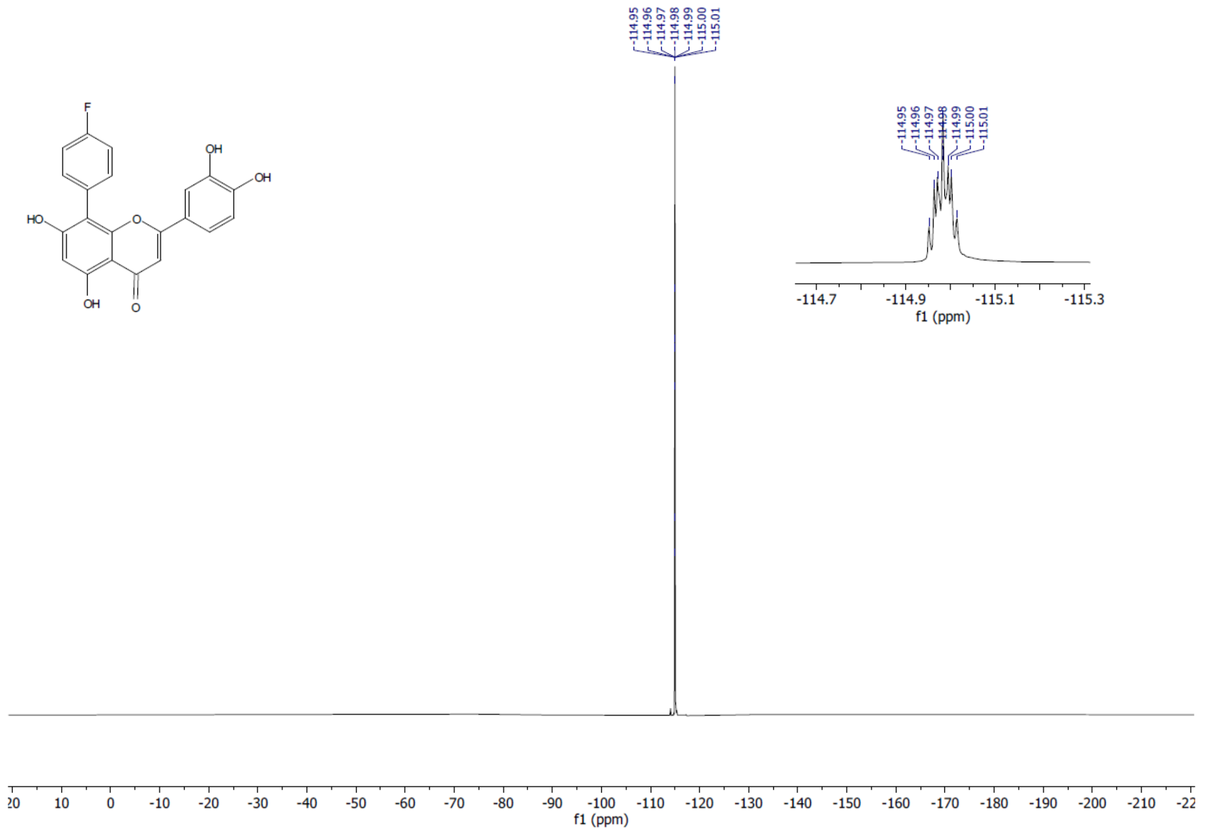

Figure S39: ^{19}F NMR (470 MHz, DMSO-*d*6, 25 °C) spectrum of **32**.

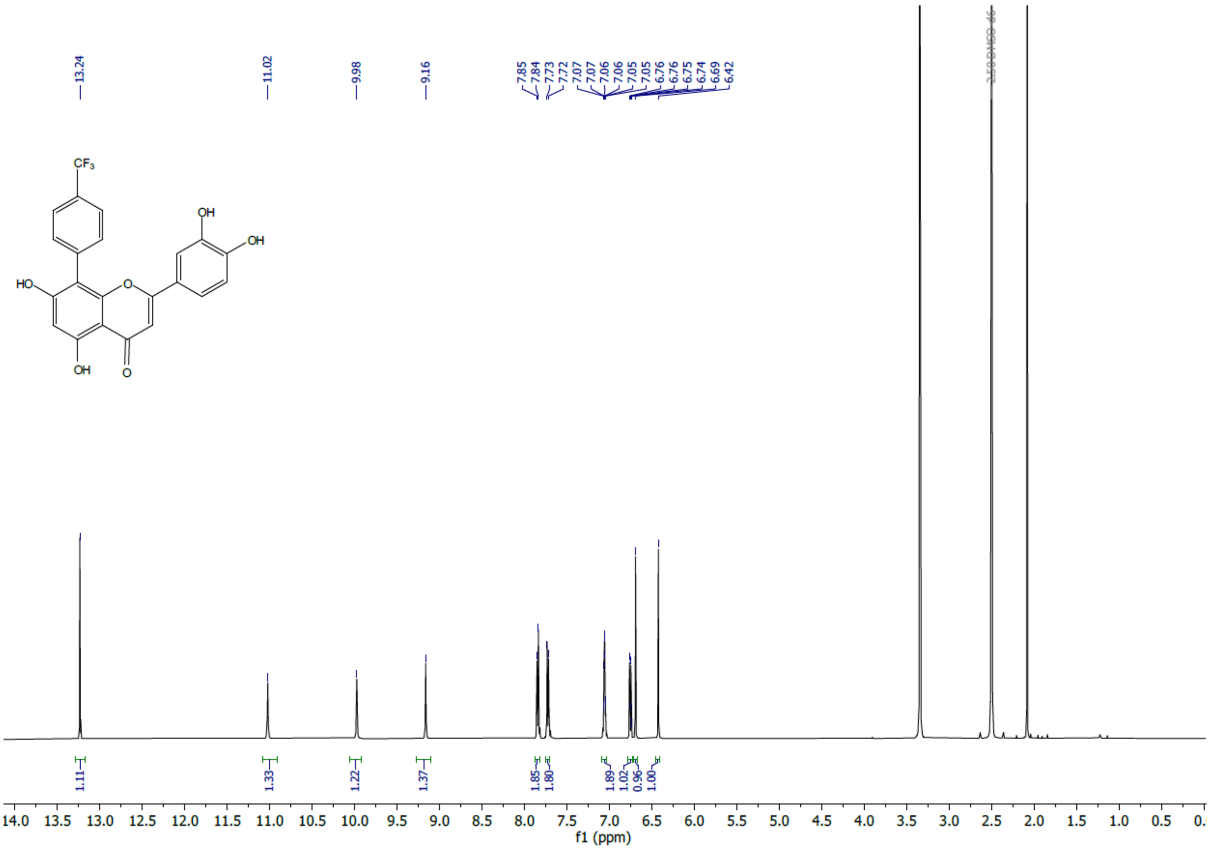

Figure S40: ^{1}H NMR (500 MHz, DMSO-d_6, 25 °C) spectrum of **33**.

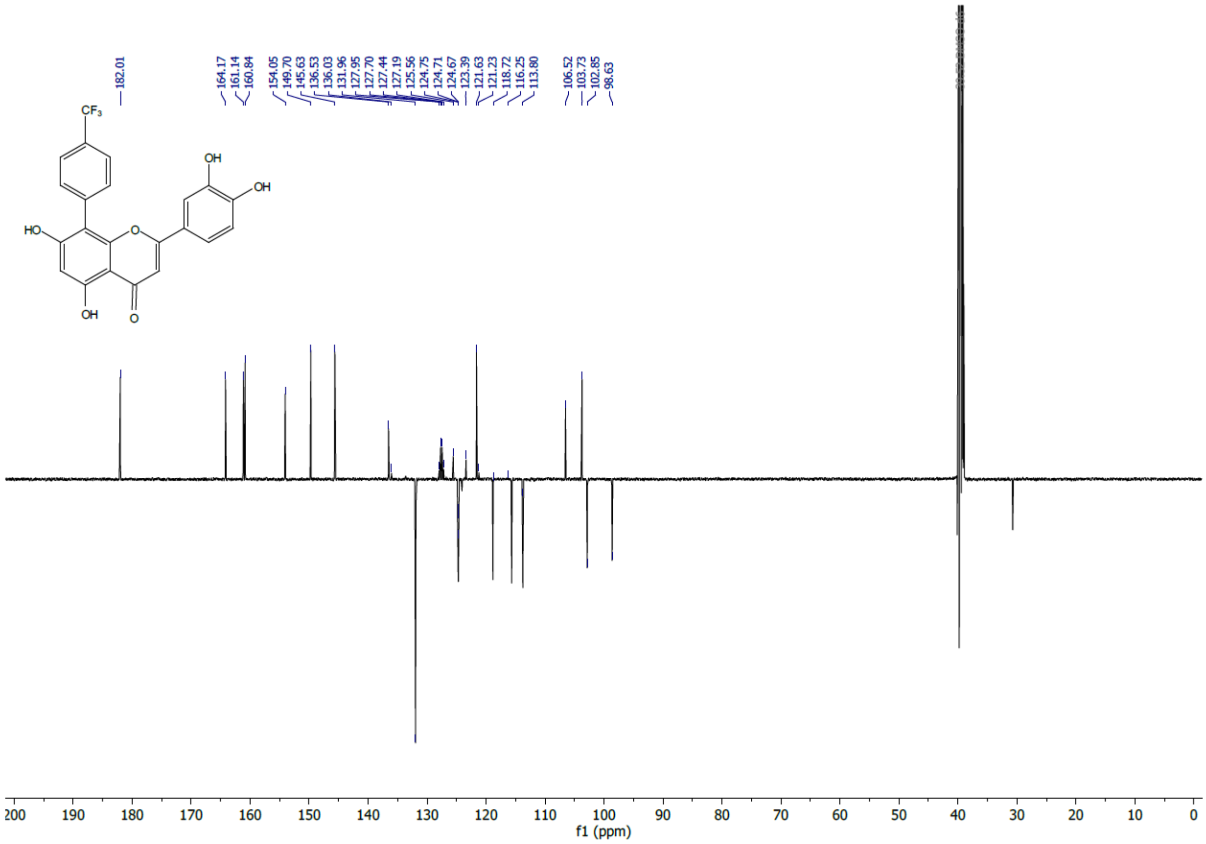

Figure S41: ^{13}C APT NMR (126 MHz, DMSO-d_6, 25 °C) spectrum of **33**.

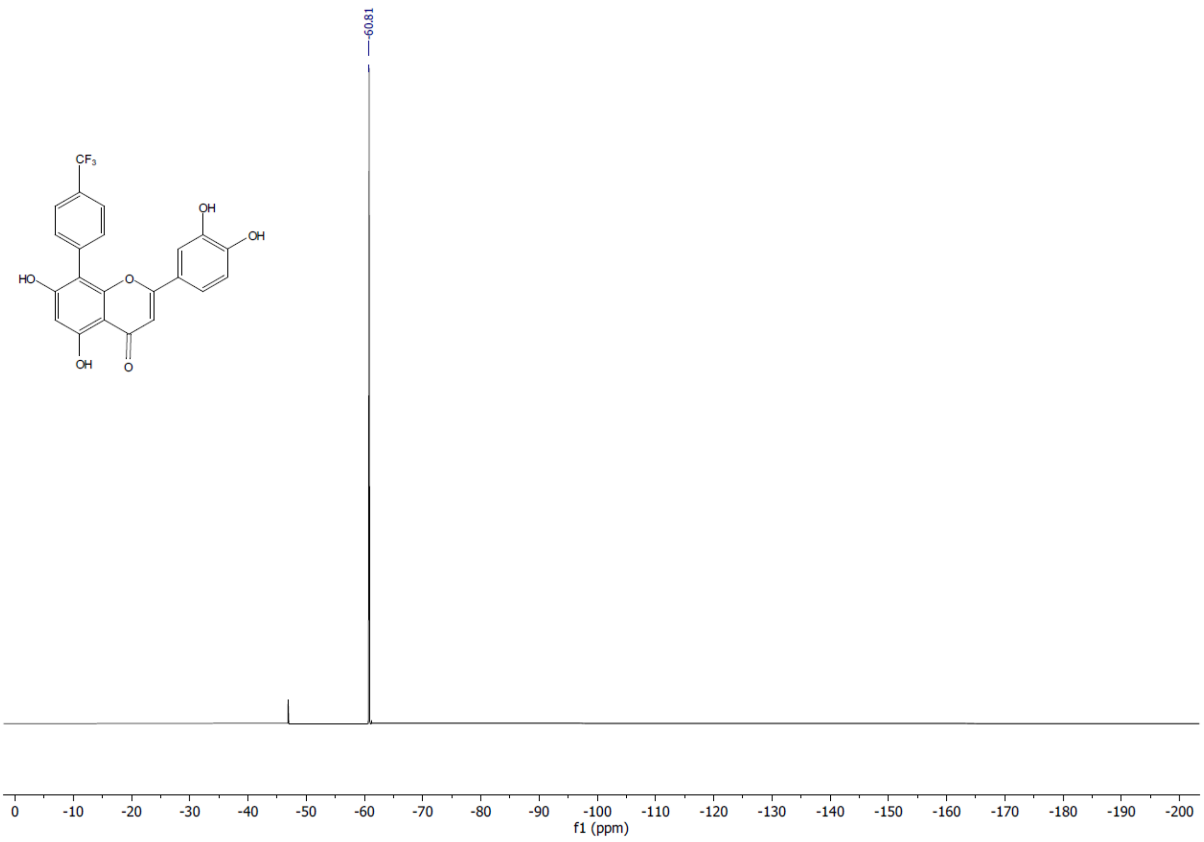

Figure S42: ^{19}F NMR (470 MHz, DMSO-*d*6, 25 °C) spectrum of **33**.

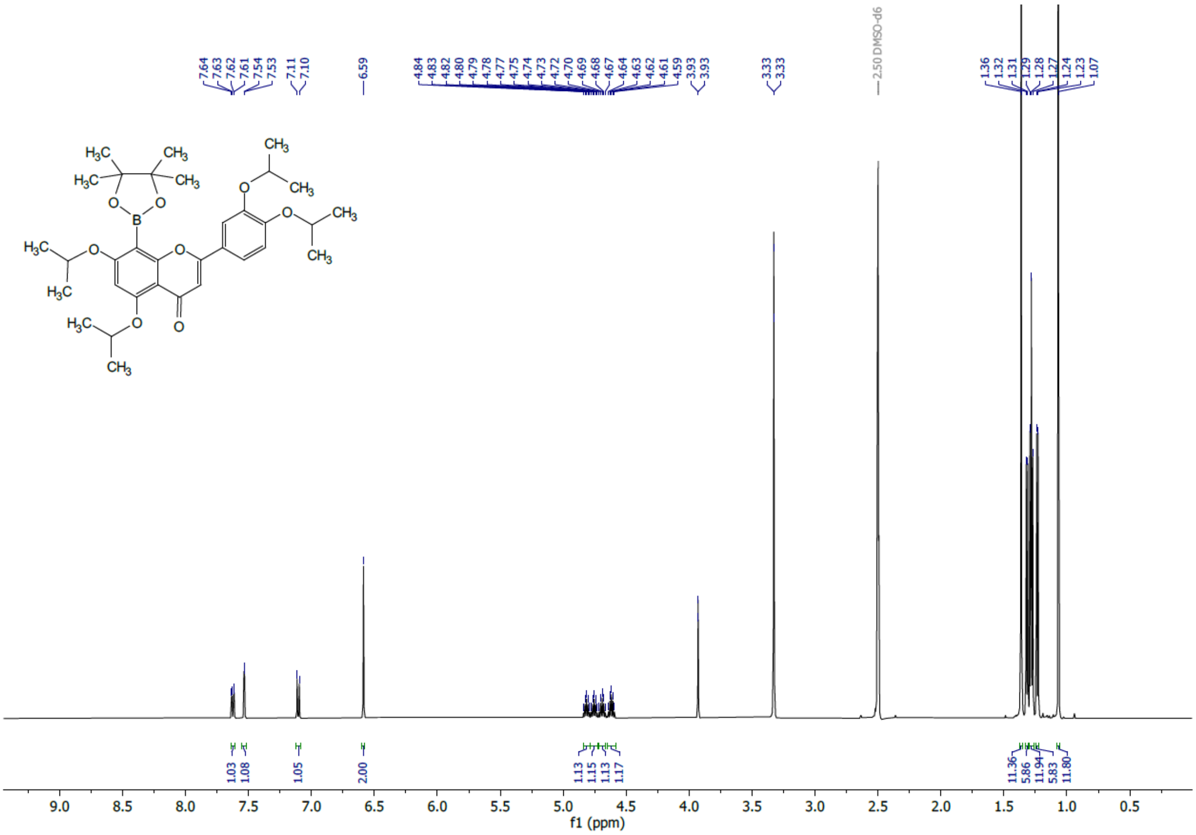

Figure S43: ^{1}H NMR (500 MHz, DMSO-*d*6, 25 °C) spectrum of **12**.

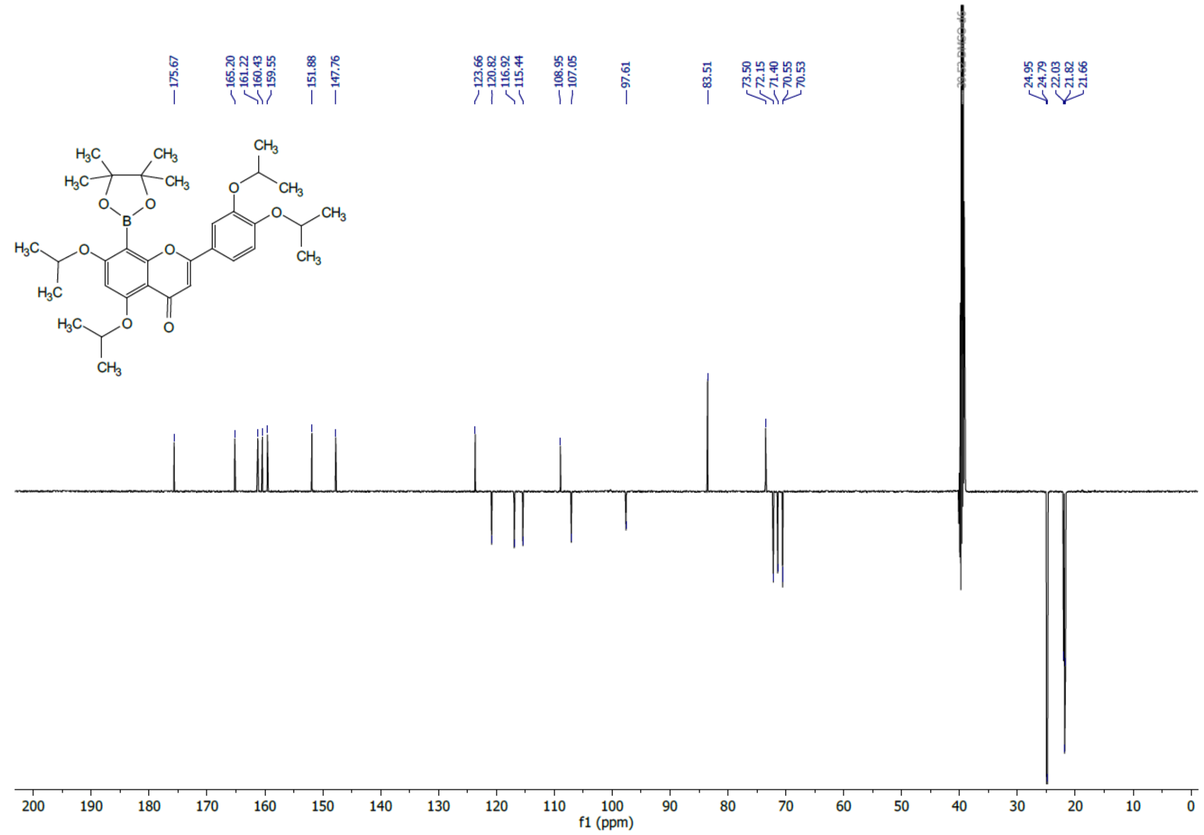

Figure S44: ^{13}C APT NMR (126 MHz, DMSO-d_6, 25 °C) spectrum of **12**.

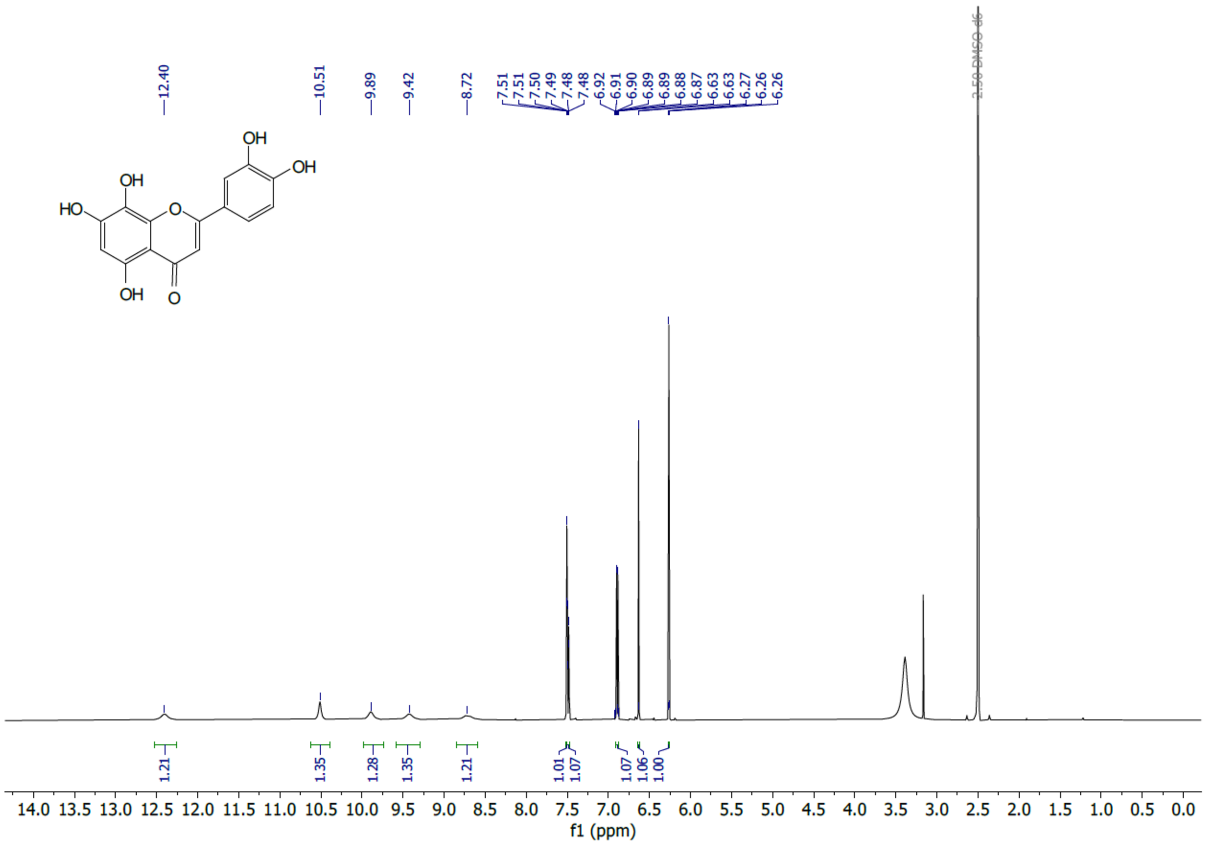

Figure S45: ^{1}H NMR (500 MHz, DMSO-d_6, 25 °C) spectrum of **34**.

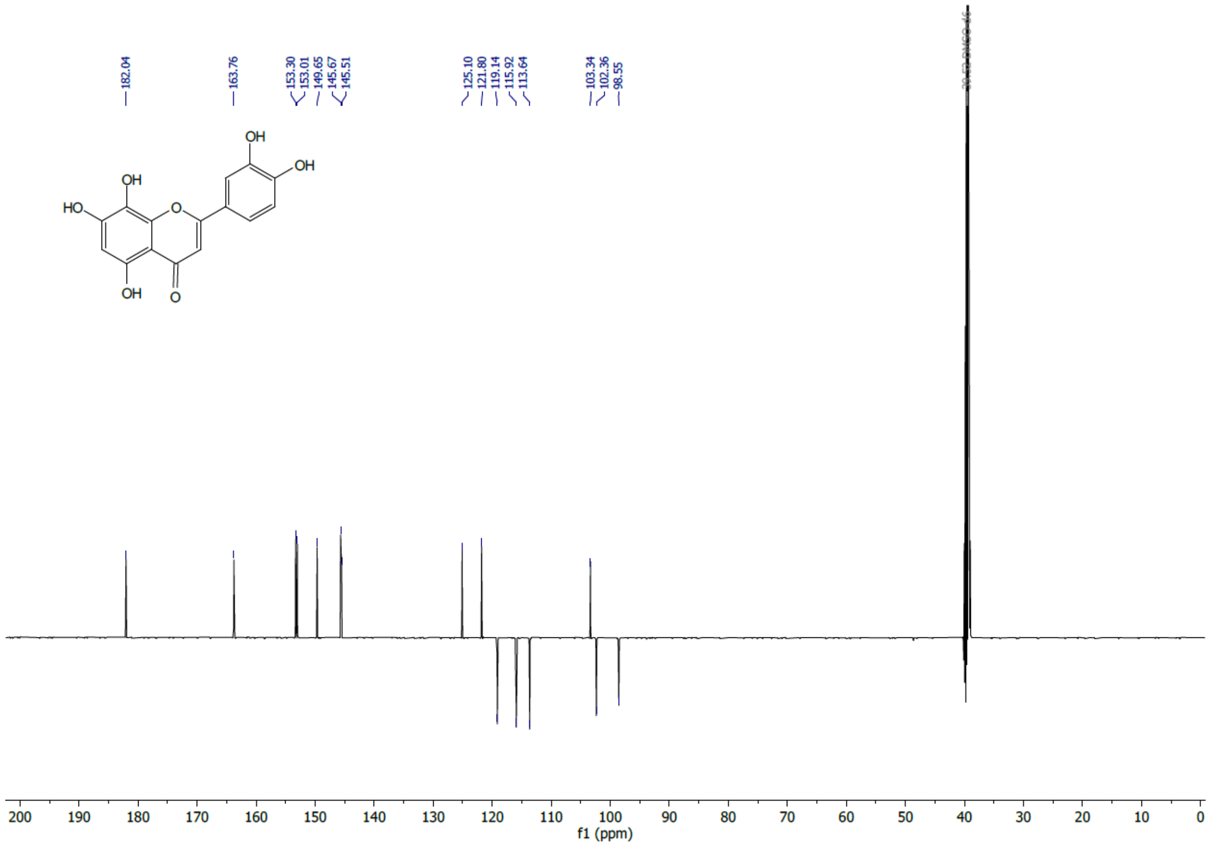

Figure S46: ^{13}C APT NMR (126 MHz, DMSO-d_6, 25 °C) spectrum of **34**.

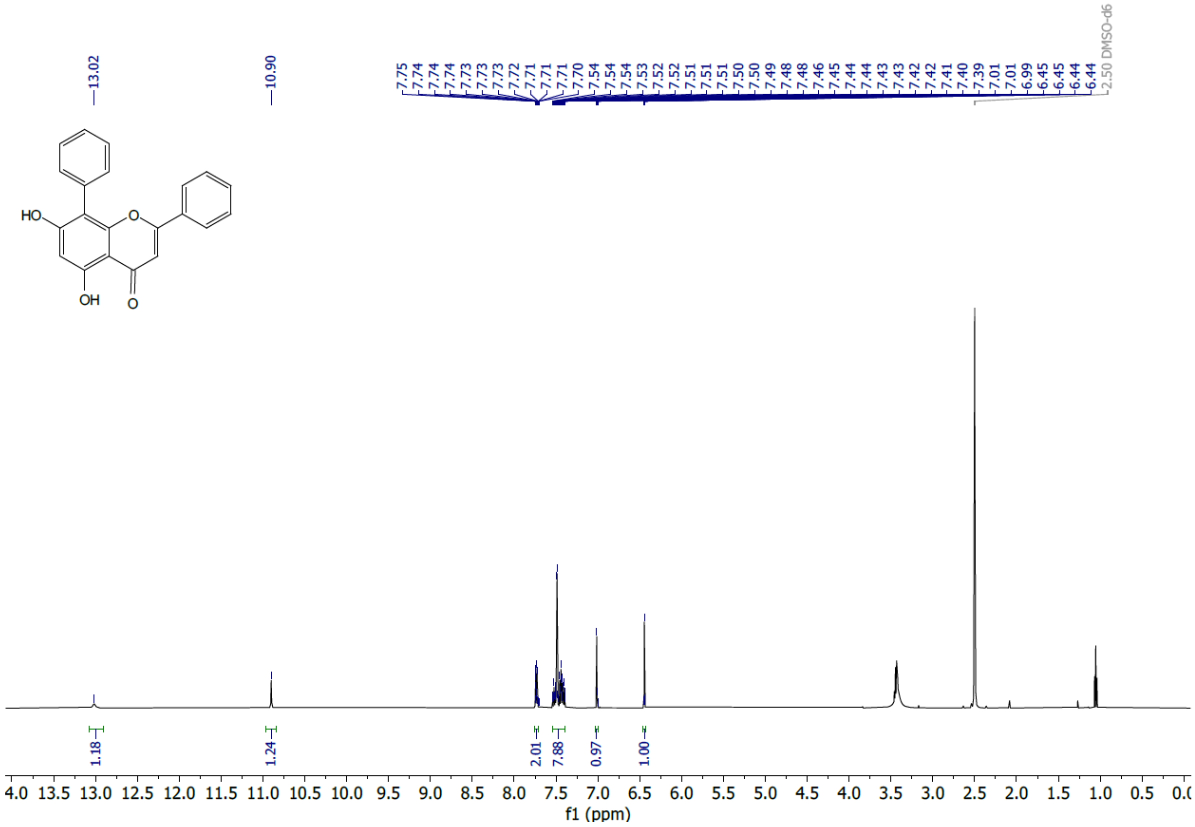

Figure S47: ^{1}H NMR (500 MHz, DMSO-d_6, 25 °C) spectrum of **35**.

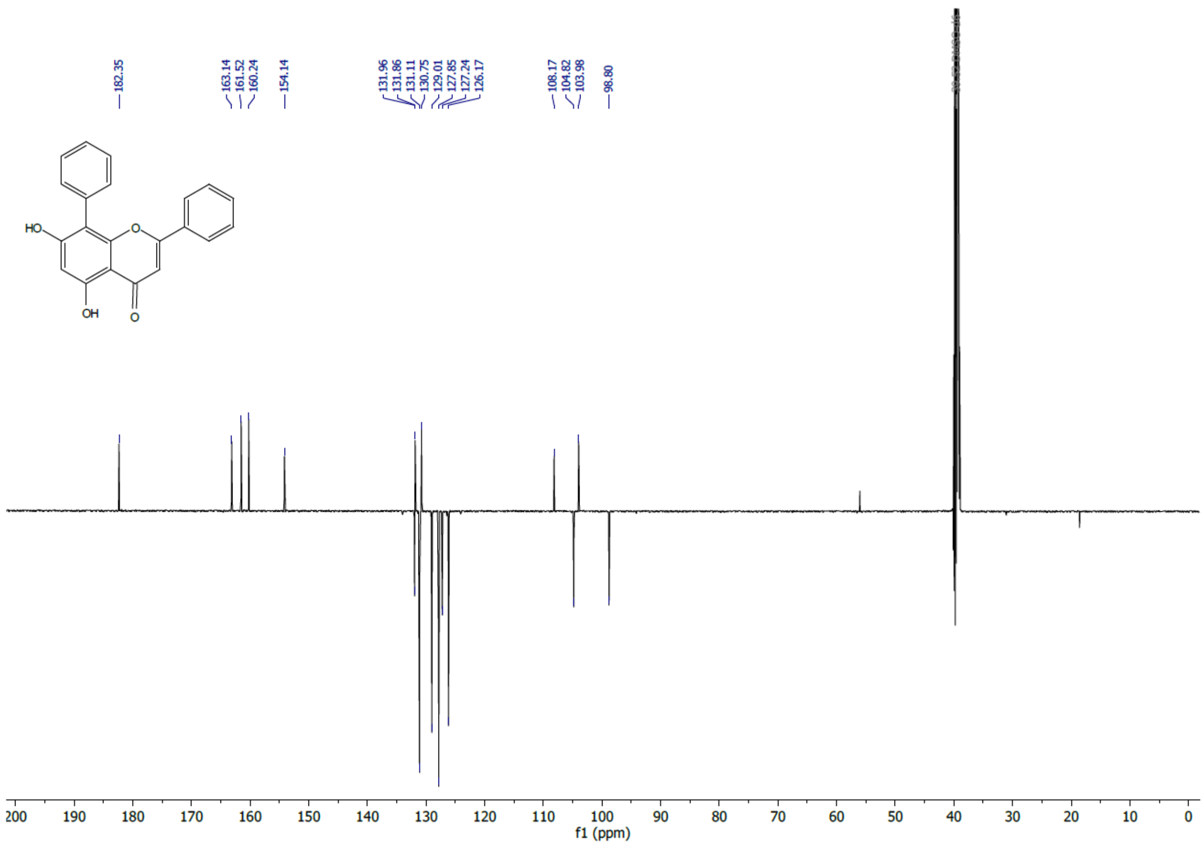

Figure S48: ^{13}C APT NMR (126 MHz, DMSO-*d*6, 25 °C) spectrum of **35**.

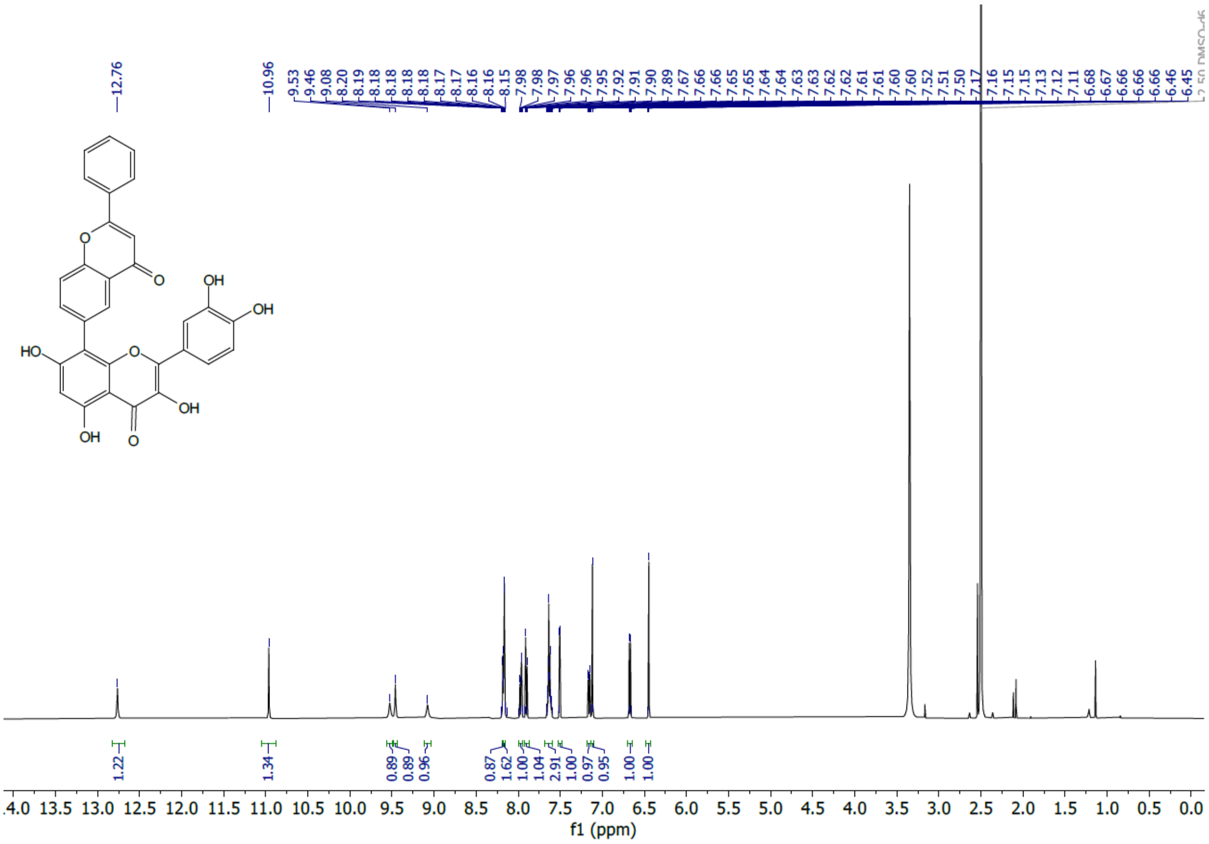

Figure S49: ^{1}H NMR (500 MHz, DMSO-*d*6, 25 °C) spectrum of **18**.

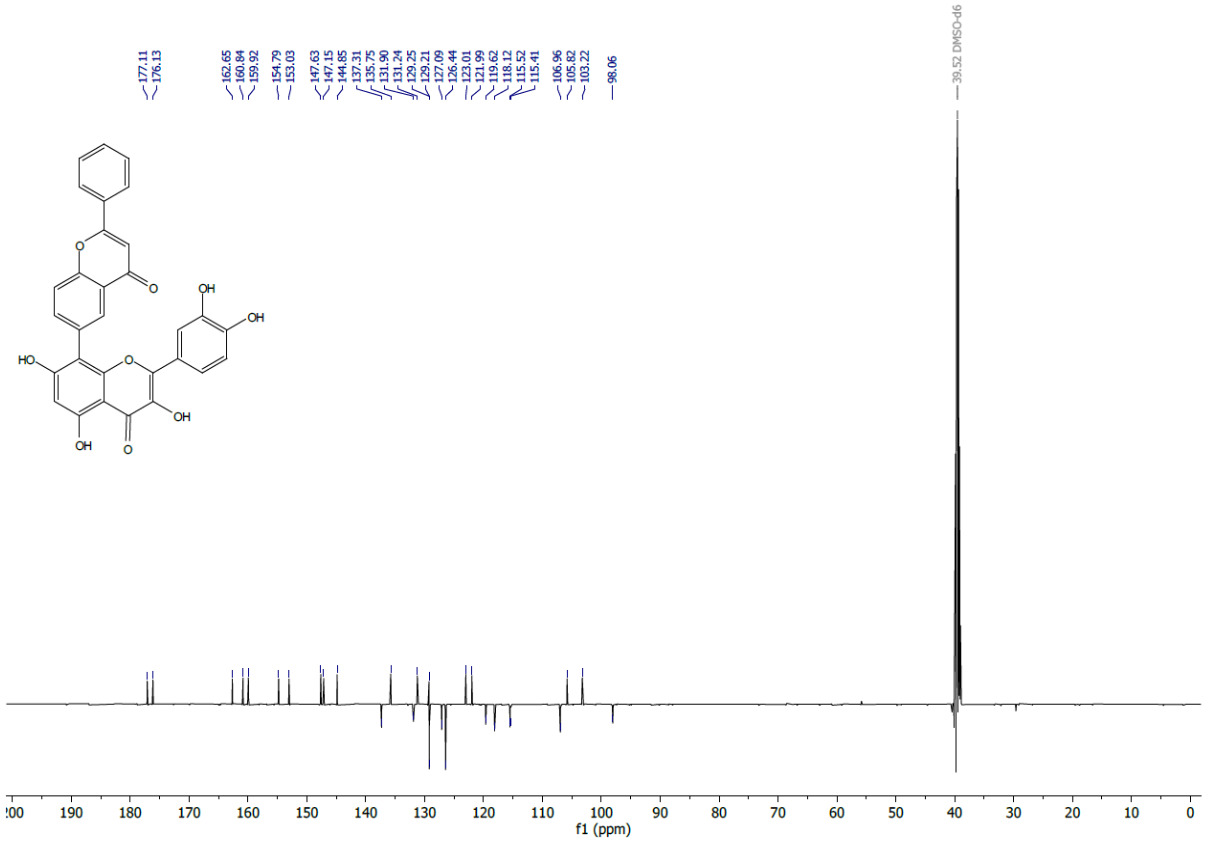

Figure S50: ^{13}C APT NMR (126 MHz, DMSO-*d*$_6$, 25 °C) spectrum of **18**.

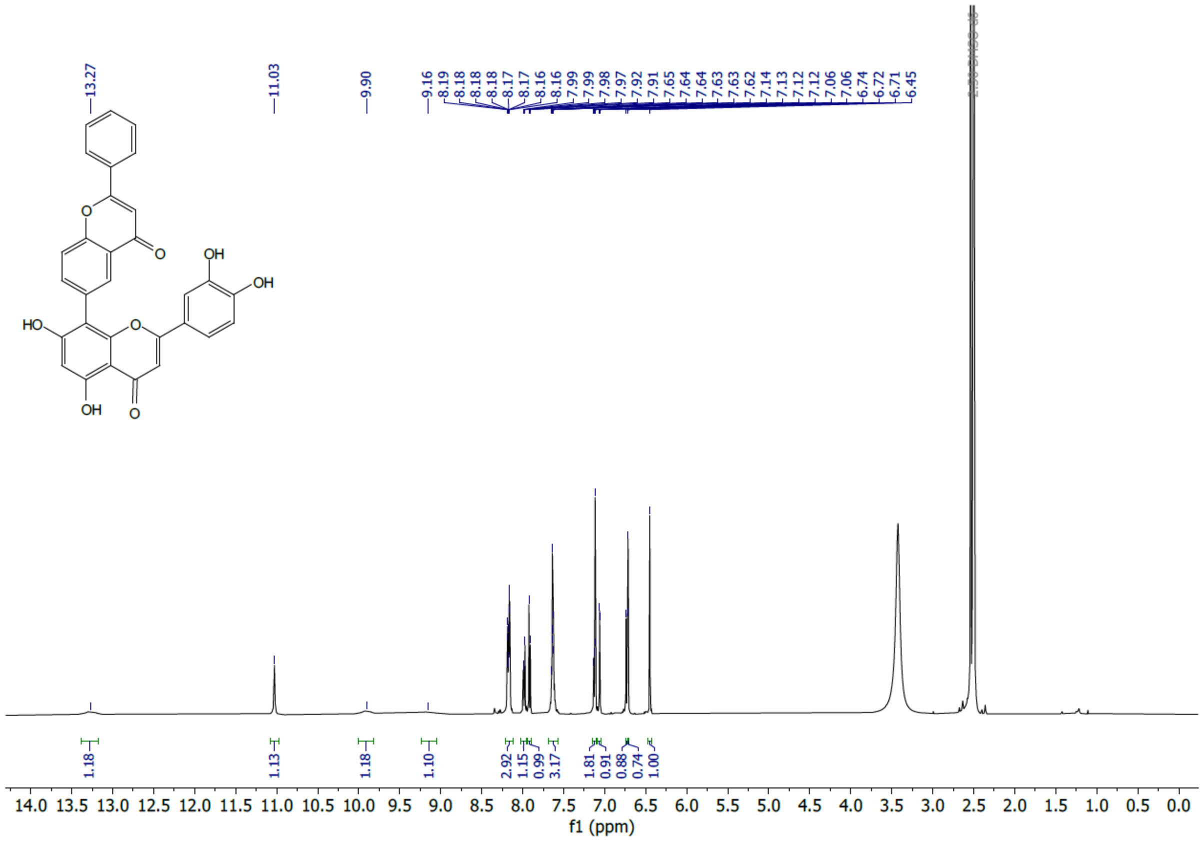

Figure S51: ^{1}H NMR (500 MHz, DMSO-*d*$_6$, 25 °C) spectrum of **19**.

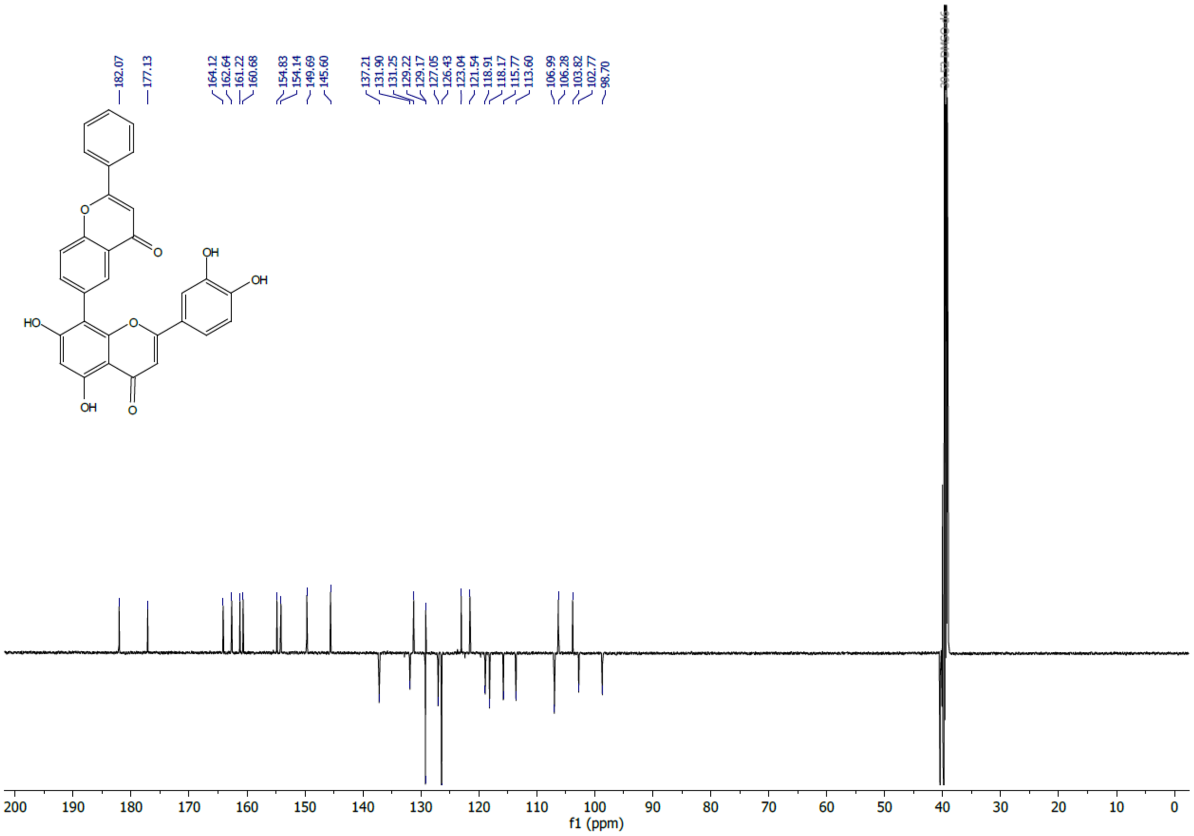

Figure S52: ^{13}C APT NMR (126 MHz, DMSO-d_6, 25 °C) spectrum of **19**.

IR Spectra

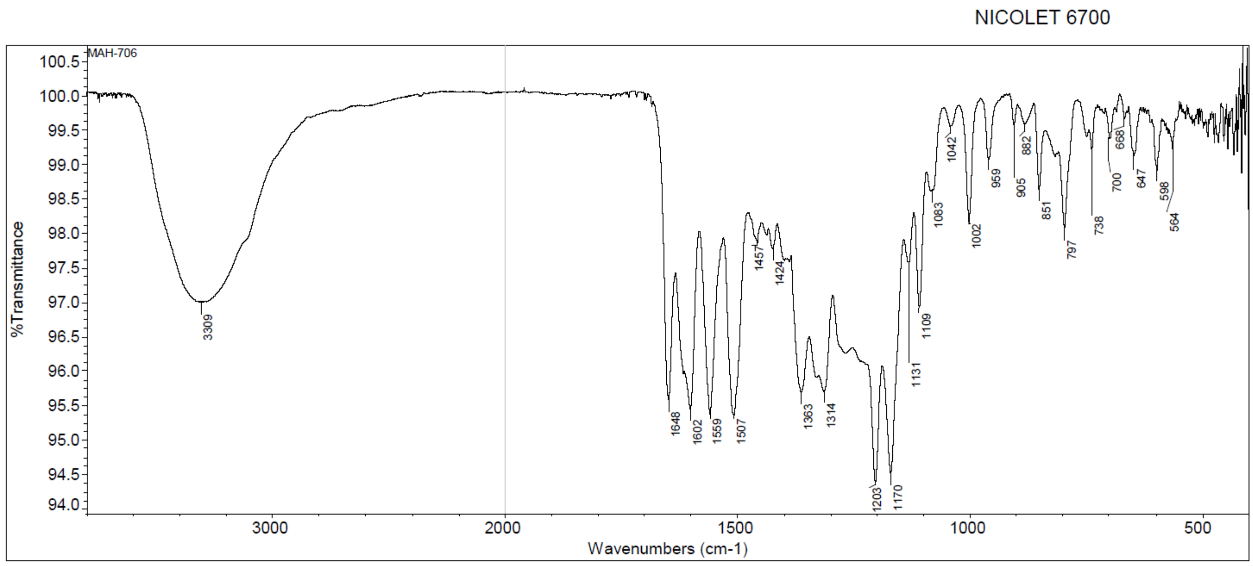

Figure S53: IR (MeOH film) spectrum of **6**.

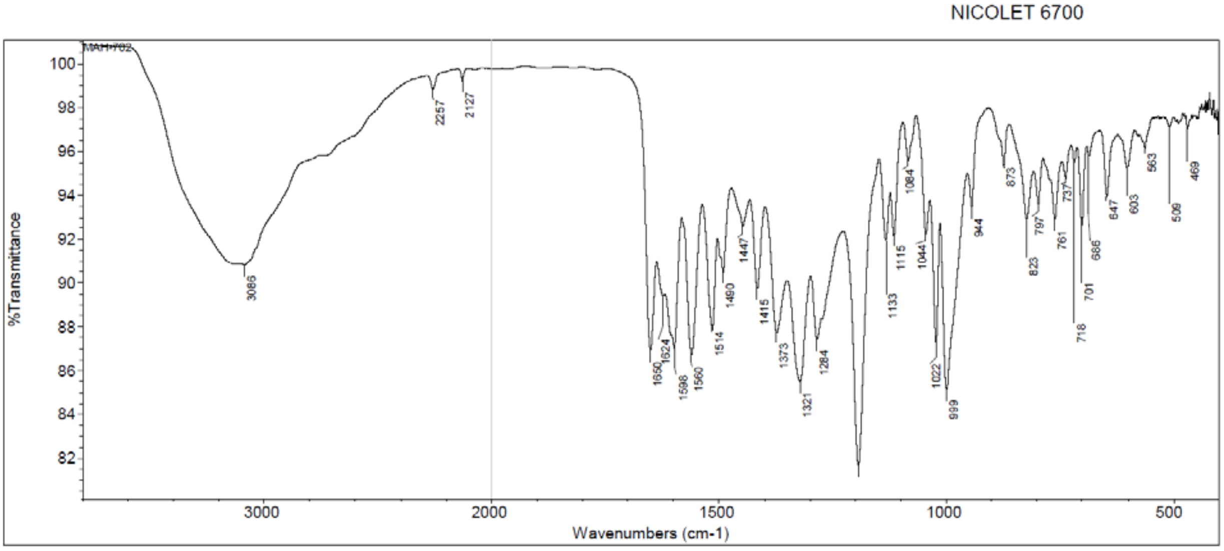

Figure S54: IR (MeOH film) spectrum of **20**.

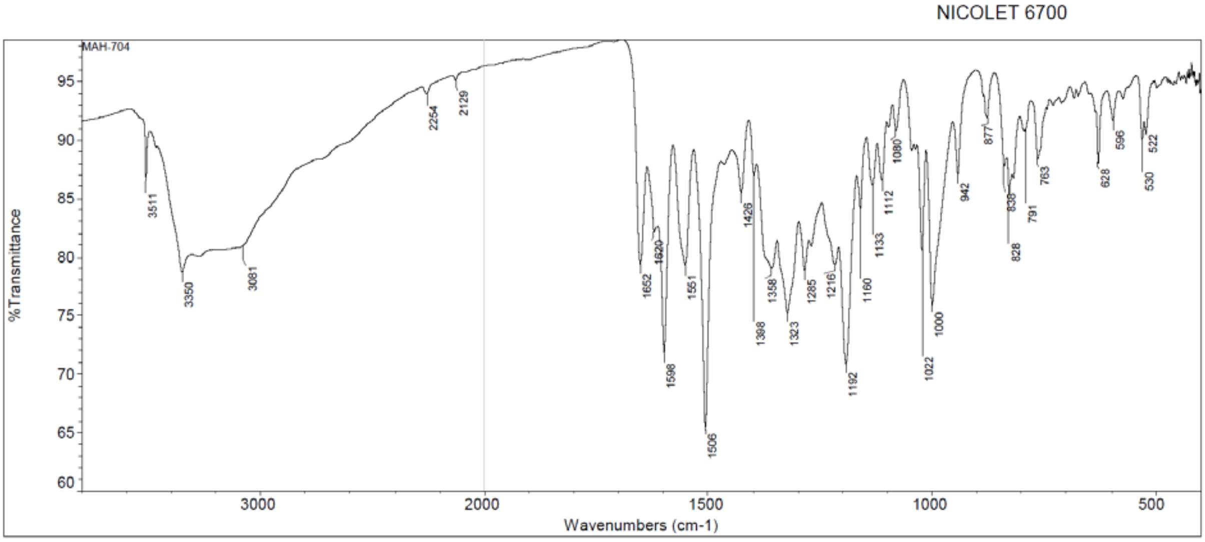

Figure S55: IR (MeOH film) spectrum of **21**.

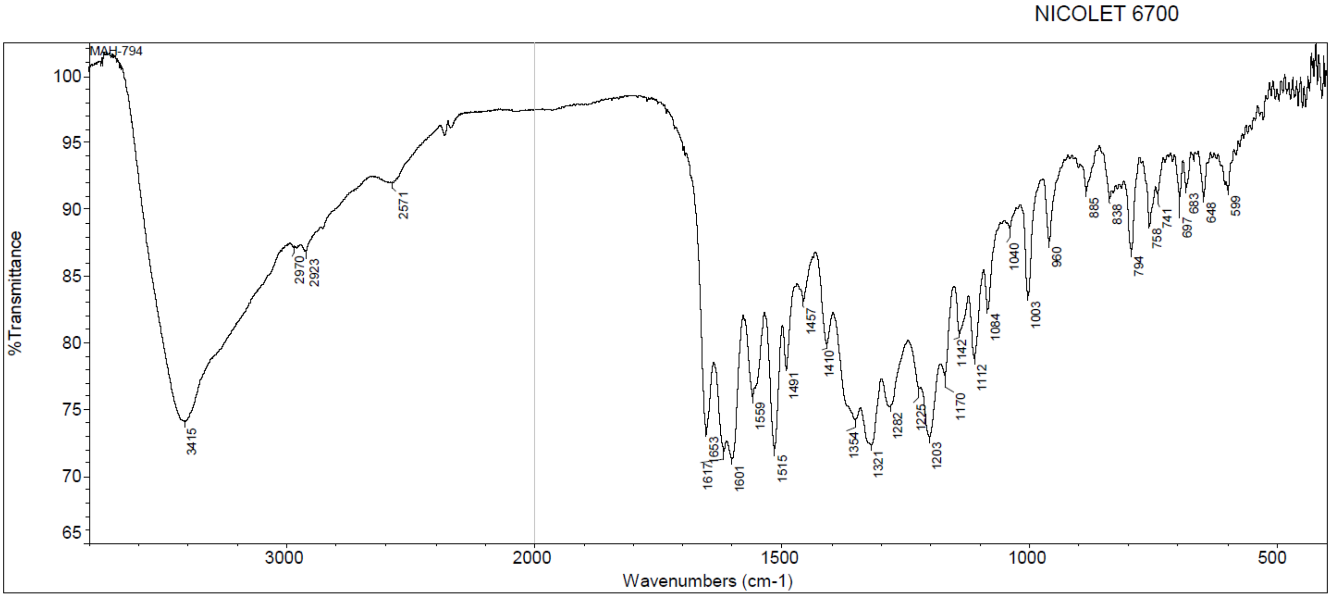

Figure S56: IR (KBr pellet) spectrum of **22**.

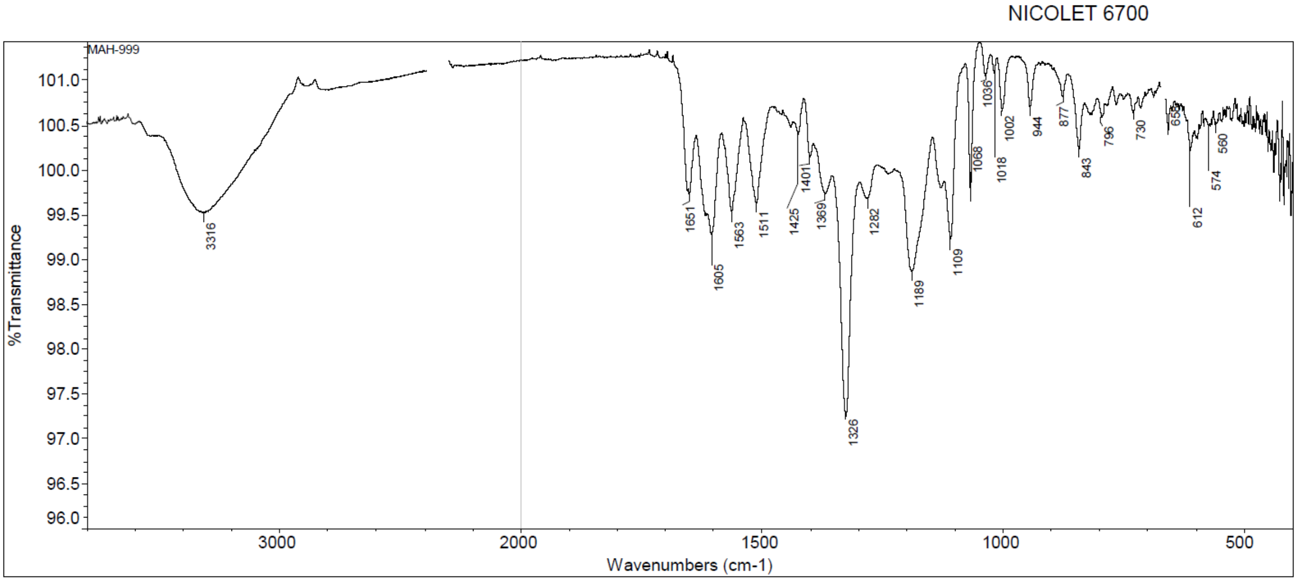

Figure S57: IR (MeOH film) spectrum of **23**.

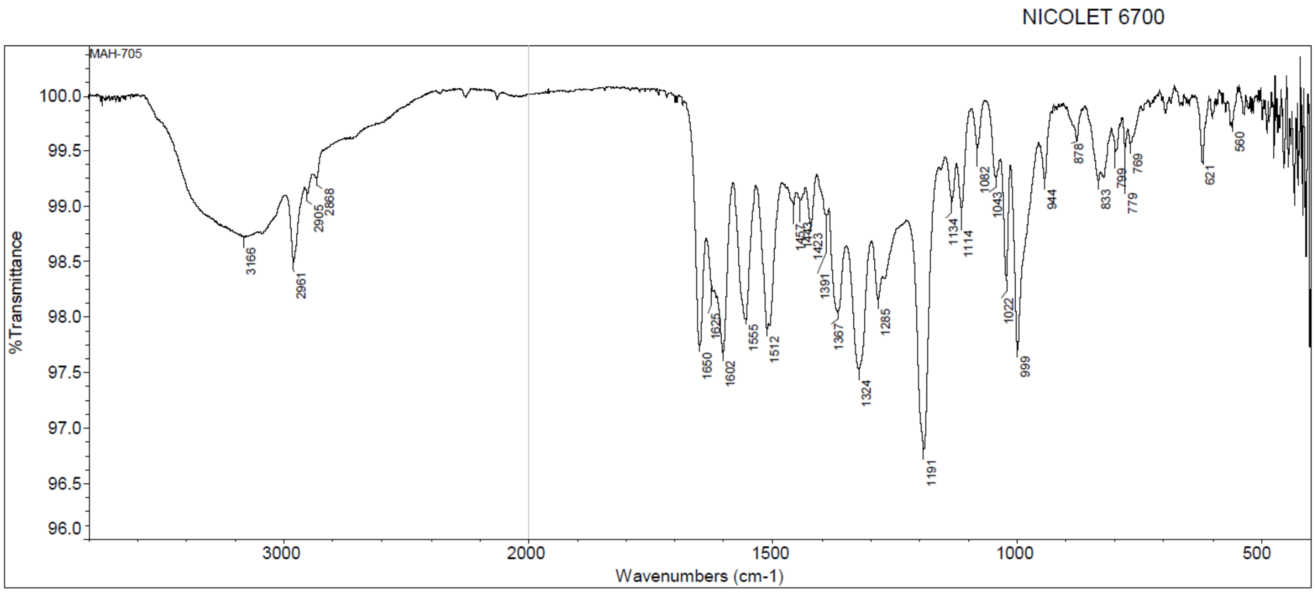

Figure S58: IR (MeOH film) spectrum of **24**.

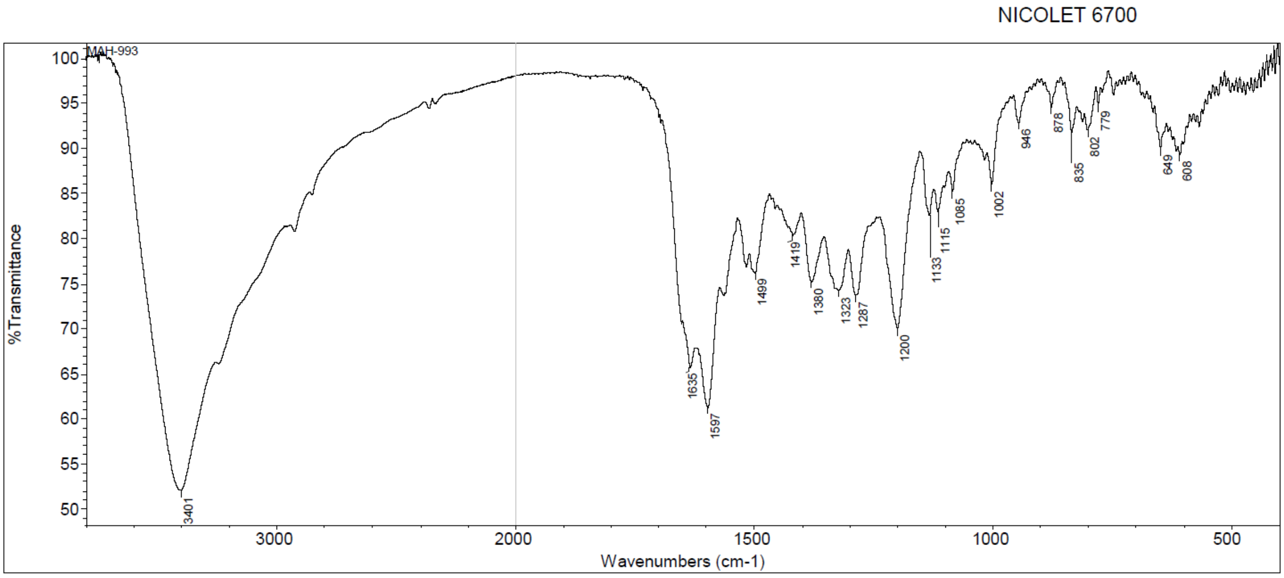

Figure S59: IR (KBr pellet) spectrum of **25**.

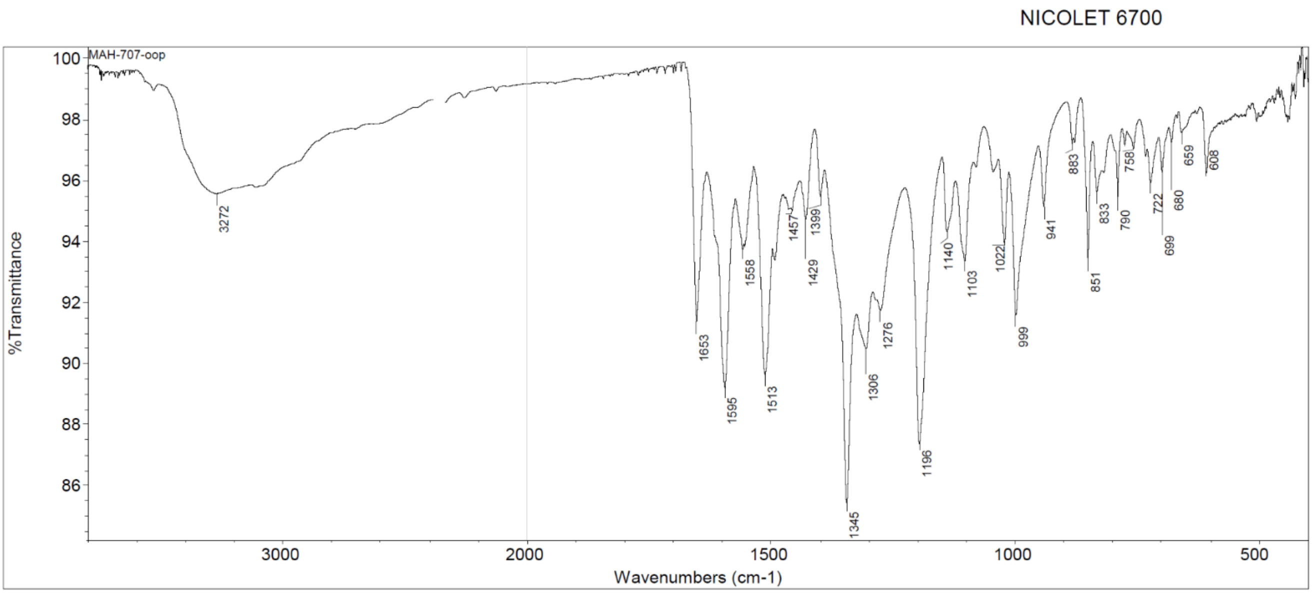

Figure S60: IR (CHCl3 film) spectrum of **27**.

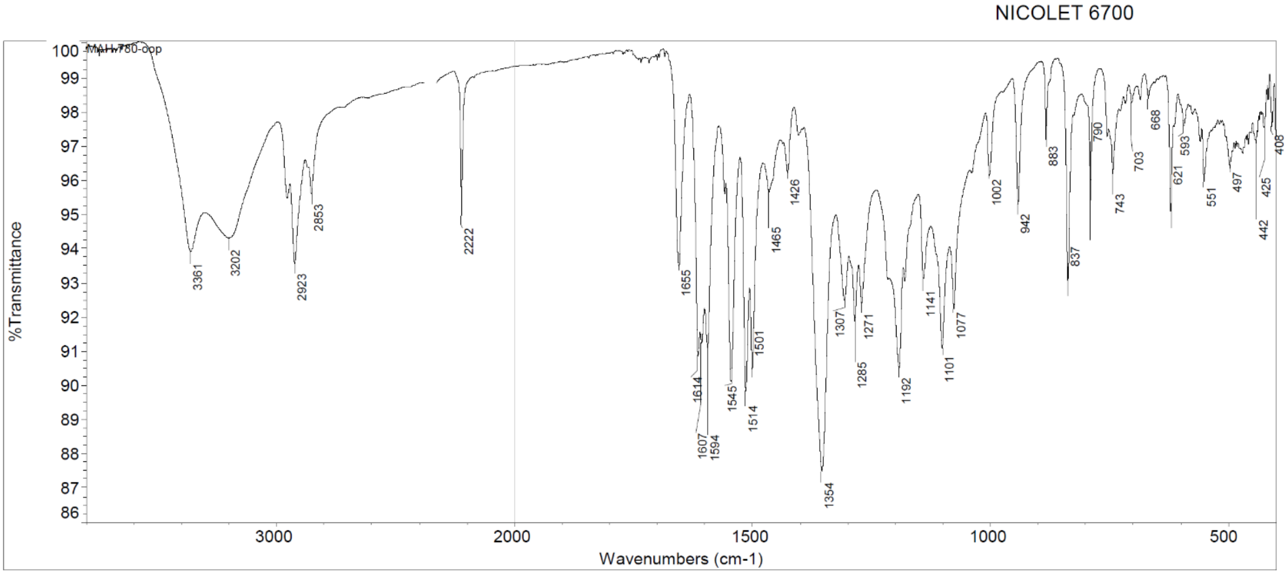

Figure S61: IR (CHCl3 film) spectrum of **28**.

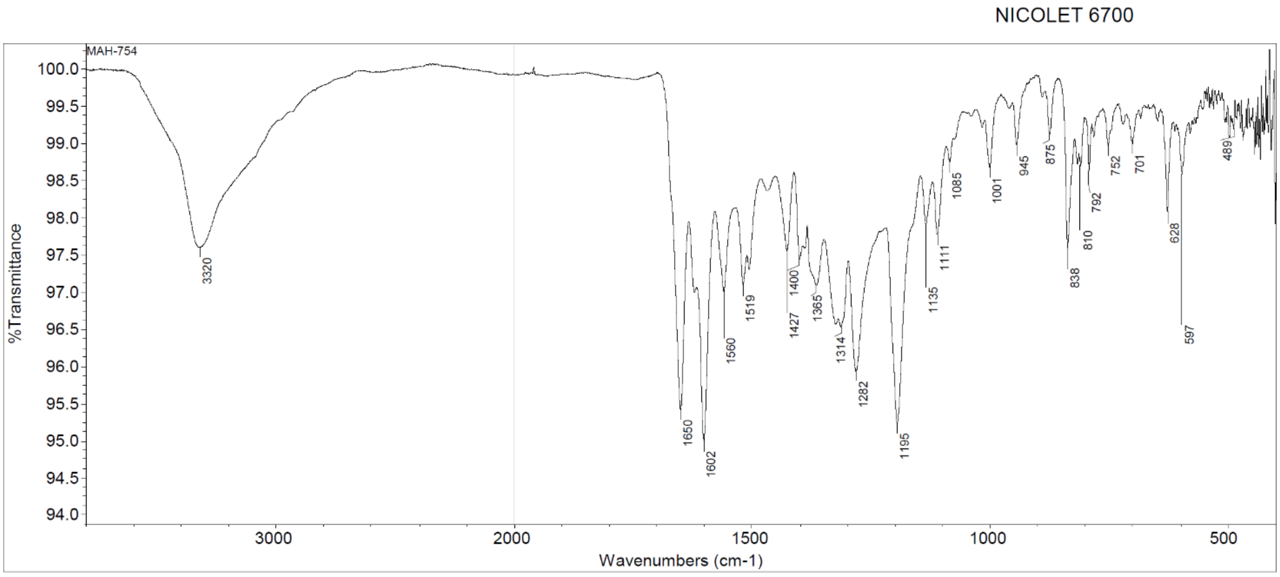

Figure S62: IR (MeOH film) spectrum of **29**.

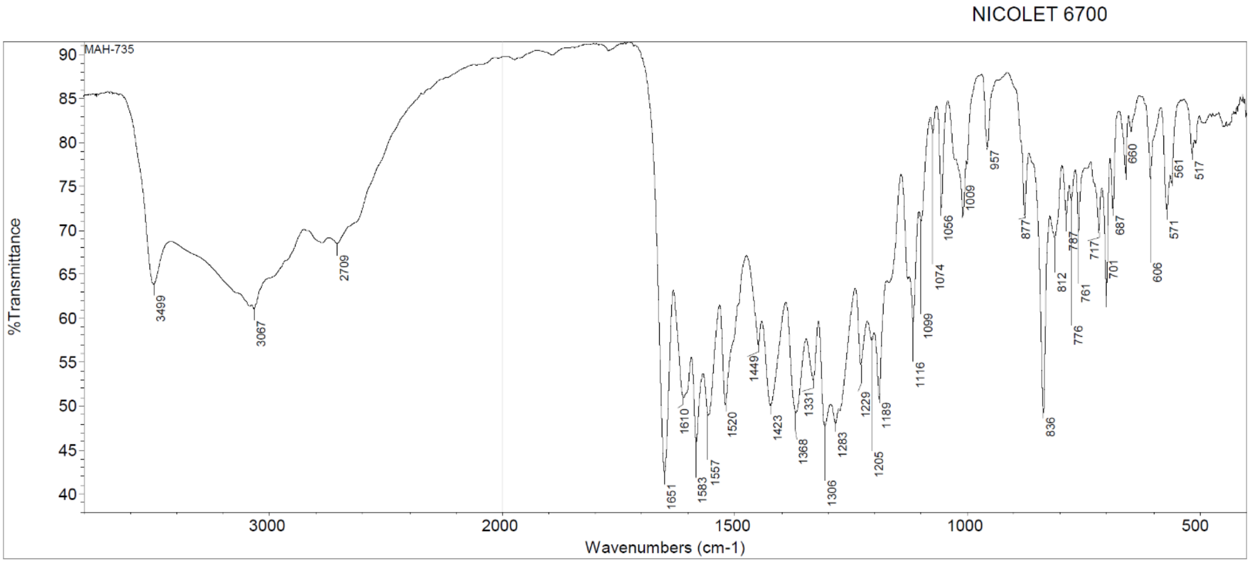

Figure S63: IR (MeOH film) spectrum of **31**.

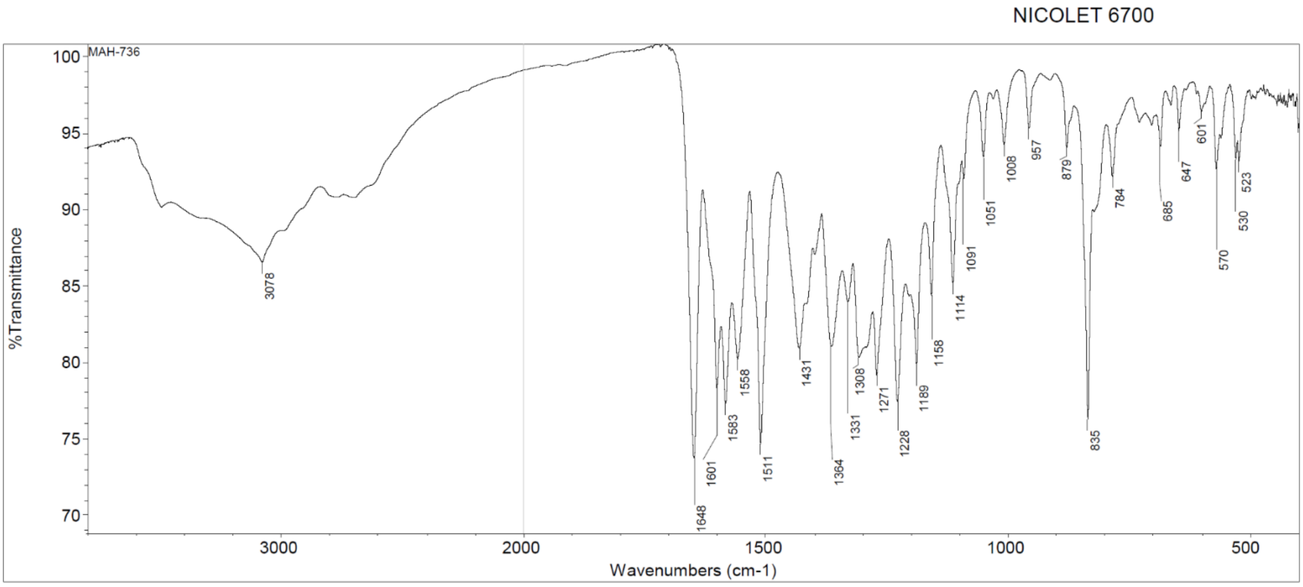

Figure S64: IR (MeOH film) spectrum of **32**.

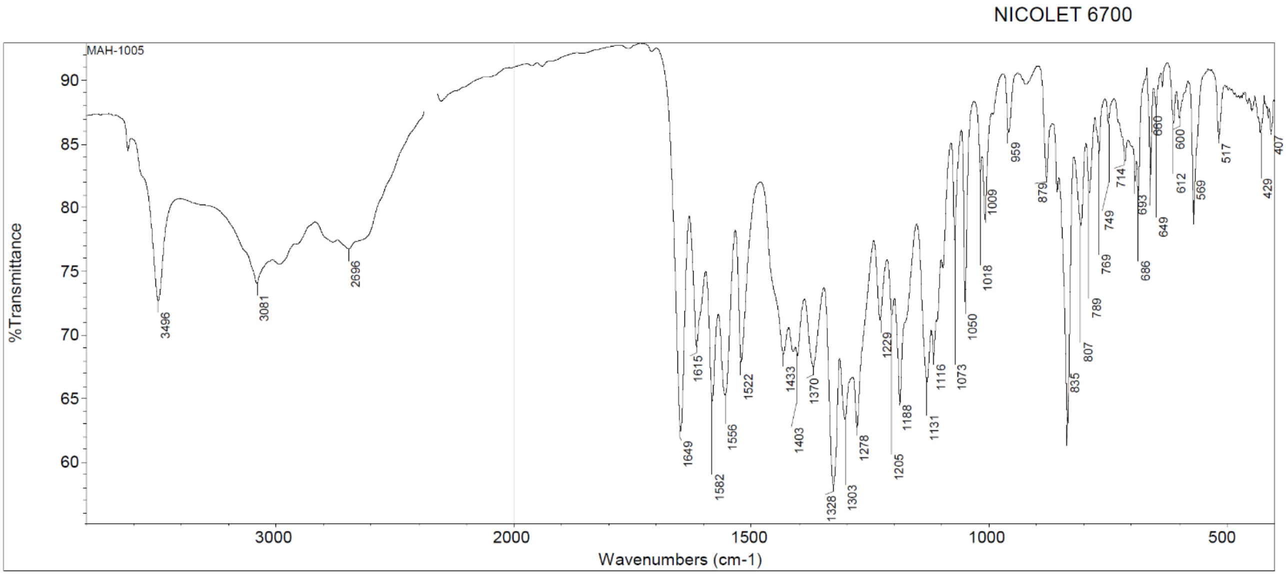

Figure S65: IR (MeOH film) spectrum of **33**.

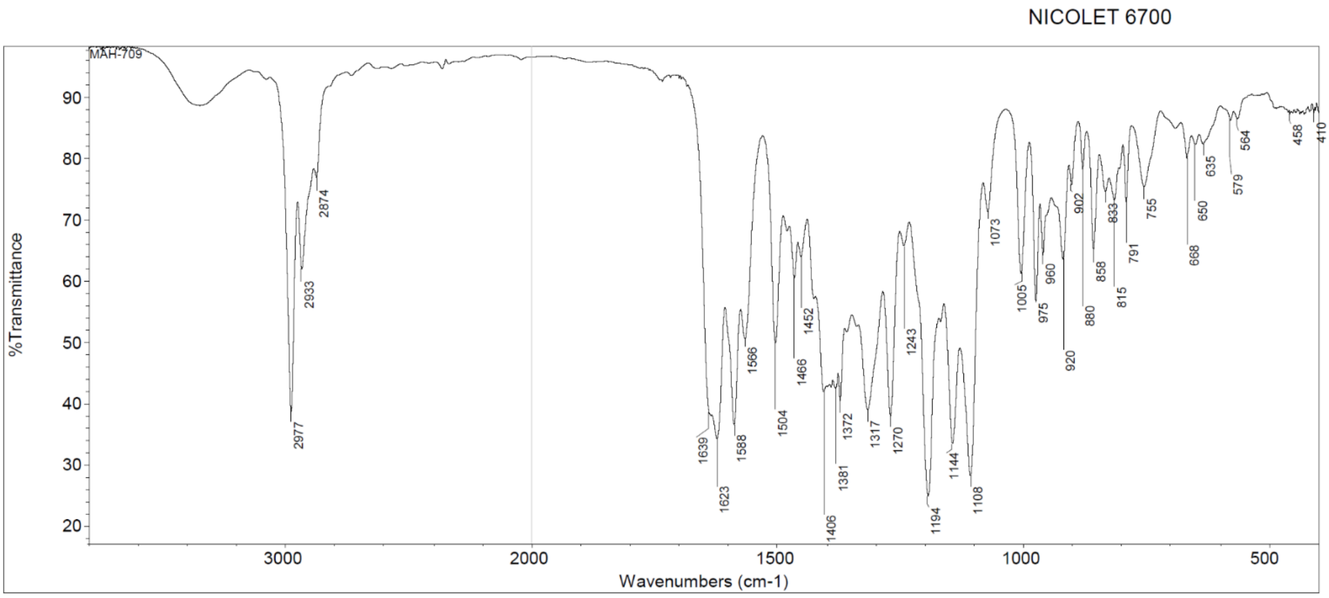

Figure S66: IR ($CHCl_3$ film) spectrum of **7**.

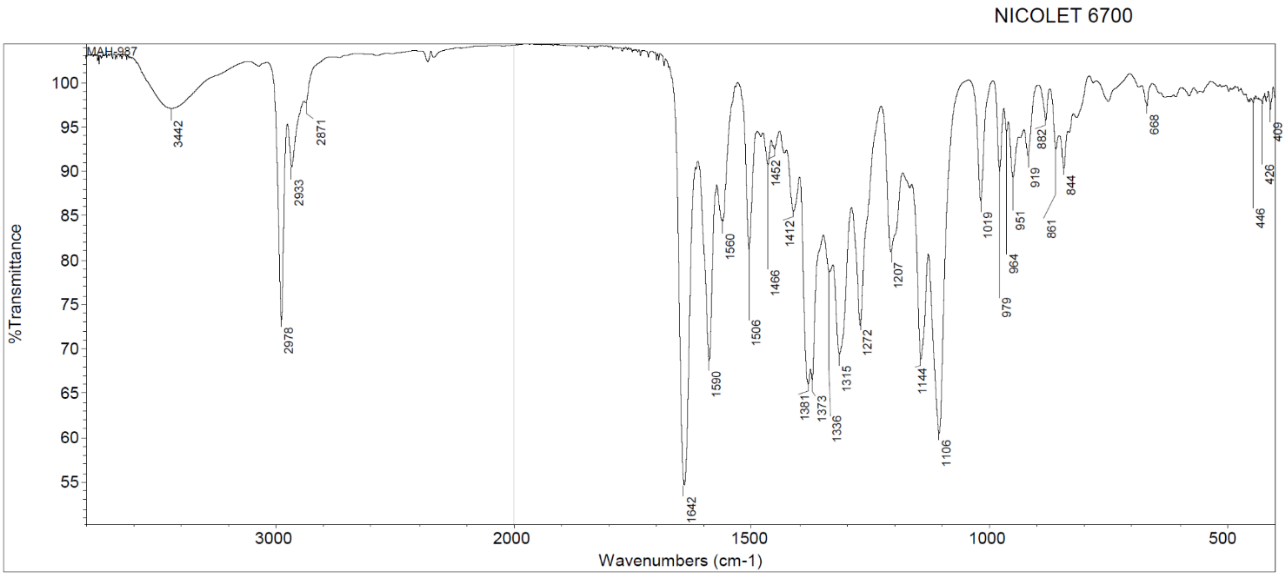

Figure S67: IR ($CHCl_3$ film) spectrum of **12**.

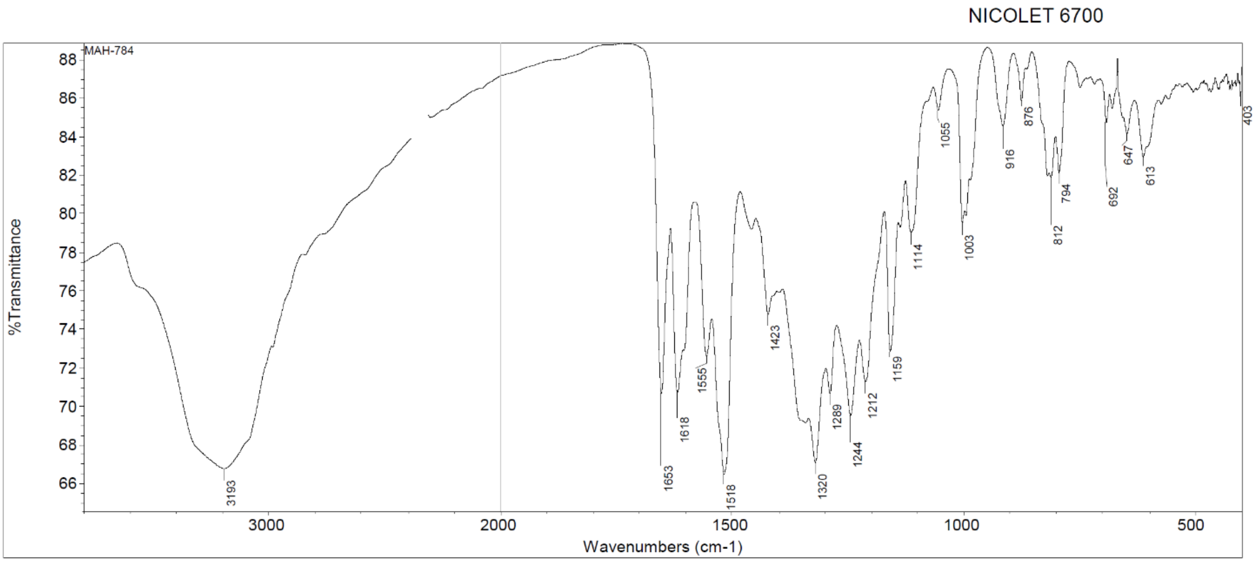

Figure S68: IR (MeOH film) spectrum of **9**.

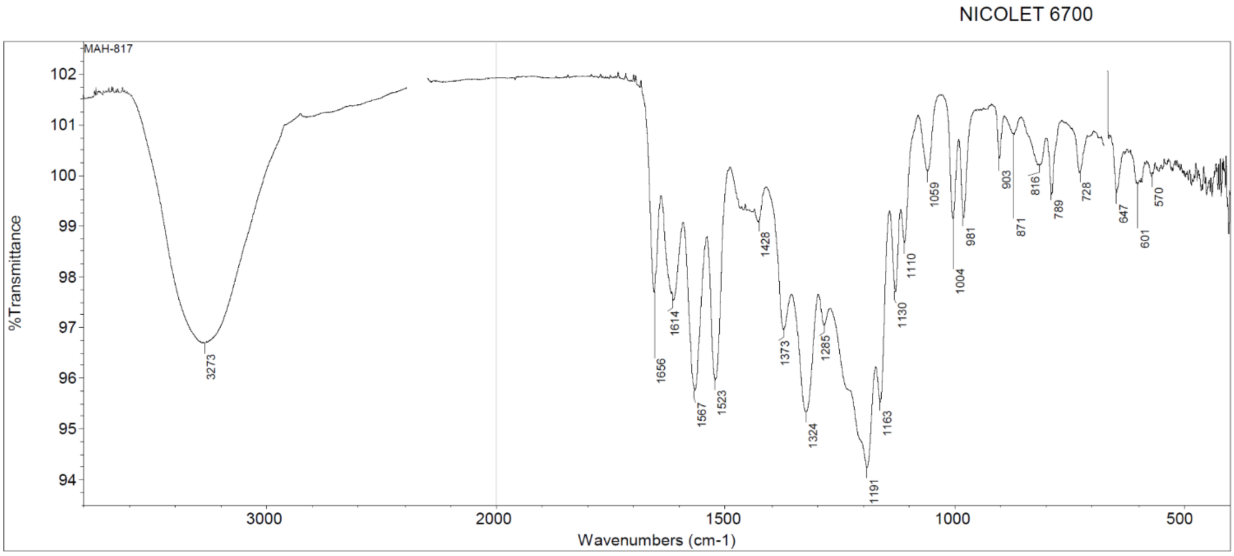

Figure S69: IR (MeOH film) spectrum of **30**.

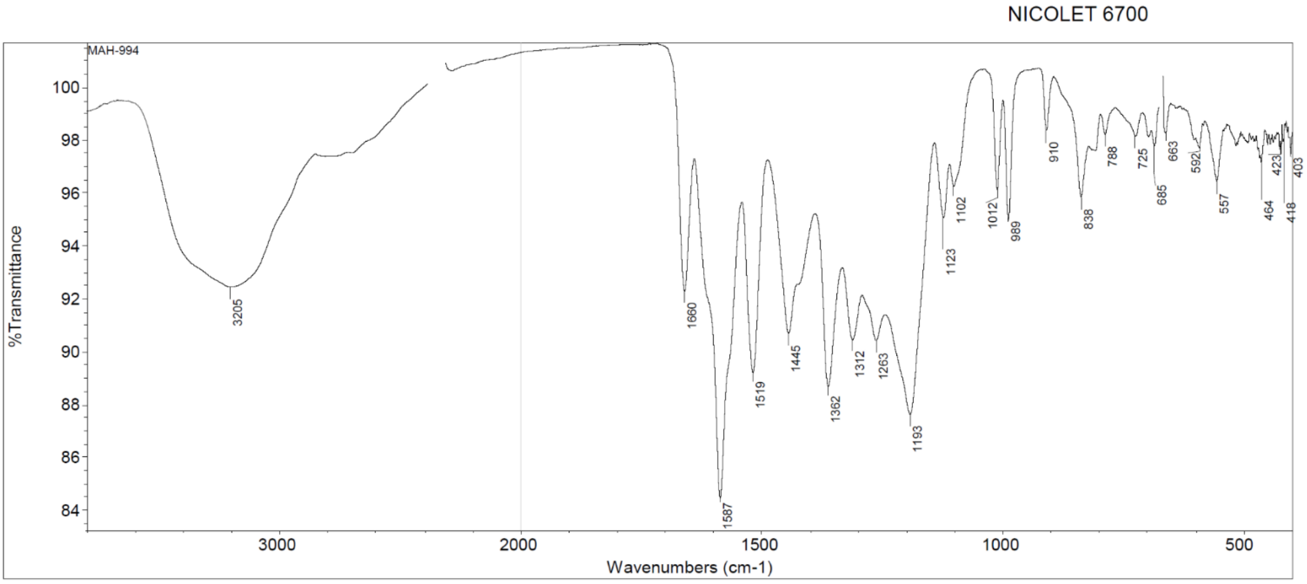

Figure S70: IR (MeOH film) spectrum of **34**.

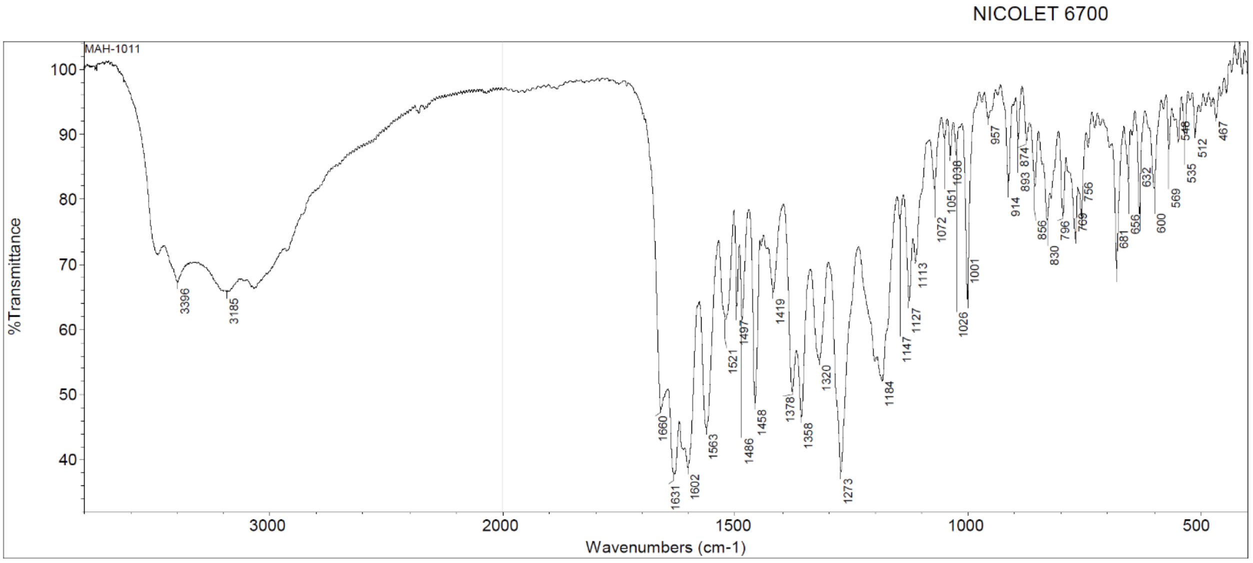

Figure S71: IR (MeOH film) spectrum of **18**.

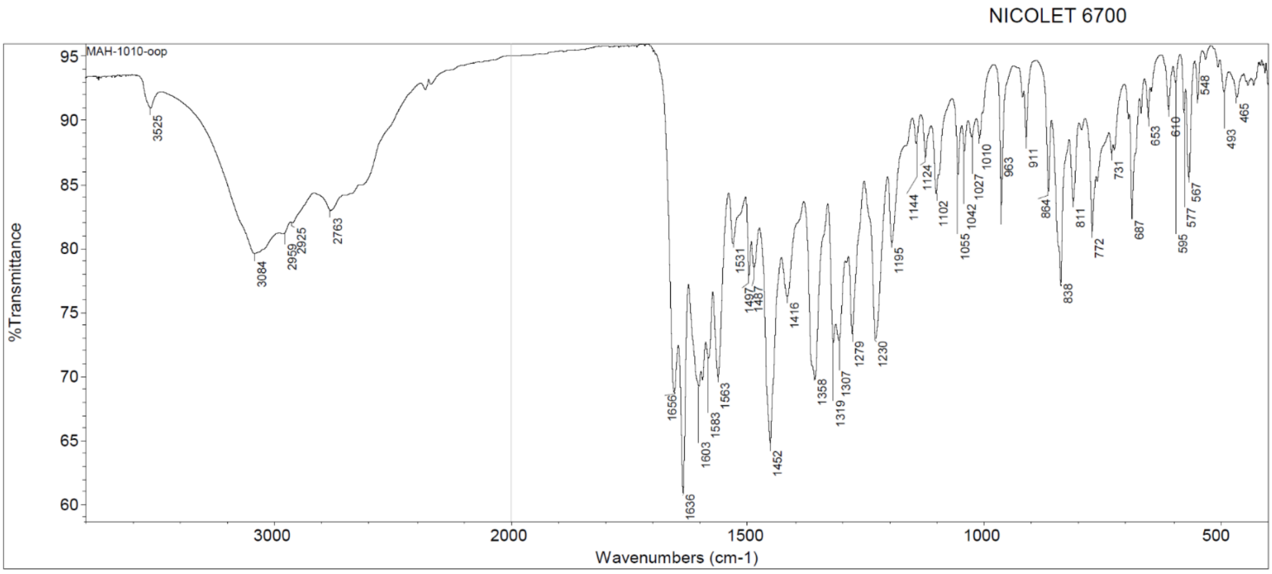

Figure S72: IR (MeOH film) spectrum of **19**.
